# Associations between wearable‐device‐measured daytime and nighttime light exposures and dementia risk: A prospective cohort study

**DOI:** 10.1002/gps3.70039

**Published:** 2026-06-24

**Authors:** Nana Zheng, Wei Wang, Biao Li, Xionge Mei, Yue Liu, Jing Du, Ngan Yin Chan, Joey W. Y. Chan, Xiaoman Xing, Xiao Tan, Christian Benedict, Yun Kwok Wing, Jihui Zhang, Hongliang Feng

**Affiliations:** ^1^ Institute of Neuropsychiatry, The Affiliated Brain Hospital, Guangzhou Medical University Guangzhou Guangdong China; ^2^ Key Laboratory of Sleep and Biological Rhythms of Guangdong Province, Center for Sleep and Circadian Medicine The Affiliated Brain Hospital, Guangzhou Medical University Guangzhou Guangdong China; ^3^ Guangdong Engineering Technology Research Center for Translational Medicine of Mental Disorders Guangzhou Guangdong China; ^4^ School of Basic Medical Sciences Guangzhou Medical University Guangzhou Guangdong China; ^5^ Centre for Preventive Neurology Wolfson Institute of Population Health, Queen Mary University of London London UK; ^6^ Li Chiu Kong Family Sleep Assessment Unit, Department of Psychiatry, Faculty of Medicine The Chinese University of Hong Kong Shatin Hong Kong China; ^7^ Li Ka Shing Institute of Health Sciences, Faculty of Medicine The Chinese University of Hong Kong Shatin Hong Kong China; ^8^ Suzhou Institute of Biomedical Engineering and Technology, Chinese Academy of Sciences Suzhou Jiangsu China; ^9^ Department of Big Data in Health Science Zhejiang University School of Public Health and Sir Run Run Shaw Hospital, Zhejiang University School of Medicine Hangzhou Zhejiang China; ^10^ Molecular Neuropharmacology, Department of Pharmaceutical Biosciences Uppsala University Uppsala Sweden; ^11^ State Key Laboratory of Respiratory Disease, Guangdong Basic Research Center of Excellence for Respiratory Medicine Guangzhou Medical University Guangzhou Guangdong China

**Keywords:** brain structures, circadian rhythm, cohort, daytime light, dementia, nighttime light, UK biobank

## Abstract

**Background:**

Identifying reliable predictors for dementia remains a critical unmet need. Light exposure plays a crucial role in regulating circadian rhythms, which influence cognitive function. However, the association between light exposure and dementia risk remains unclear.

**Aims:**

This study examined the associations of daytime and nighttime light exposure with dementia risk.

**Methods:**

A total of 87 577 dementia‐free participants (mean age: 62.36 years; 56.98% female) were included. Daytime and nighttime light exposures were measured using 7‐day free‐living wrist‐worn accelerometry. Incident dementia was identified from primary care, hospital inpatient admissions and death registry data. Cox proportional hazards models assessed associations, and mediation analyses evaluated circadian rest–activity rhythms (CRARs), brain structures and vitamin D as potential mediators.

**Results:**

Over a median follow‐up of 8.1 years, 741 participants developed dementia. Daytime light exposure above 1000 lux was associated with reduced dementia risk (hazard ratio [HR] 0.84, 95% confidence interval [CI] 0.71–0.99, *p* = 0.039). Longer exposure to brighter light (e.g., ≥ 0.70 h at ≥ 5000 lux; HR 0.83, *p* = 0.036) was associated with a further reduction in risk. In exploratory analyses, CRARs and brain structures mediated up to 33% of the association. Protective associations were stronger in those with high levels of nighttime light exposure, an evening chronotype or *apolipoprotein E (APOE)* ε4 carrier status, with a risk reduction of up to 41%. Furthermore, < 0.70 h per day of bright daytime light (≥ 5000 lux) outperformed six established dementia predictors (e.g., obesity, alcohol consumption, traumatic brain injury and so on). Nighttime light showed no significant association with dementia risk.

**Conclusions:**

High levels of daytime light exposure were significantly associated with lower dementia risk. Further research should explore its role in dementia screening and inform the development of light‐based interventions.

## Abstract (highlights)

**WHAT IS ALREADY KNOWN ON THIS TOPIC**

- Reliable, modifiable predictors for dementia are urgently needed to inform prevention strategies. Light exposure is a primary cue for the circadian system, which regulates cognitive function; however, its association with the risk of incident dementia remains unclear.

**WHAT THIS STUDY ADDS**

- Higher levels of daytime light exposure are significantly associated with a reduced risk of dementia, with an effect size larger than that of many established predictors. The study identifies specific brain structures (e.g., fusiform cortex) as statistically significant mediators and suggests that rest–activity rhythms and other brain structures may act as exploratory mediators. The protective association is strongest in individuals with higher nighttime light exposure, an evening chronotype or *APOE* ε4 carrier status.

**HOW THIS STUDY MIGHT AFFECT RESEARCH, PRACTICE OR POLICY**

- These findings position daytime light exposure as a novel, robust and measurable indicator of dementia risk. They could inform future research into light‐based interventions and public health guidelines that recommend increased daytime light exposure as a low‐cost strategy to support brain health, particularly in high‐risk populations.

## INTRODUCTION

Dementia, the most common neurodegenerative disease worldwide, is characterised by progressive cognitive decline and impairments in daily functioning.^1^, ^2^ As the global population ages, the rapidly increasing prevalence of dementia, coupled with limited effective treatments, presents a growing health and socioeconomic challenge.^3^, ^4^ Therefore, there is an urgent need to identify protective factors and establish effective prevention strategies.^5^

The natural light–dark cycle, characterised by darkness at night and bright light during the day, is a fundamental environmental cue that entrains endogenous circadian rhythms.^6^ This circadian entrainment regulates physiology, behaviour and cognition.^6^, ^7^, ^8^ Conversely, circadian disruptions are commonly found in persons with dementia and are associated with a higher risk of dementia in the general population.^2^, ^9^, ^10^, ^11^ In addition, bright light therapy (BLT), typically exceeding 3000 lux, is a promising nonpharmacological intervention that can improve both circadian disruptions and cognitive symptoms in patients with dementia.^12^, ^13^ Nevertheless, modern lifestyles limit daytime exposure to natural bright light, as individuals spend most of their time indoors under insufficient ambient lighting,^14^ whereas excessive nighttime light exposure, as detected by satellite data, affects nearly 80% of the population.^15^ Given these concerns, it is crucial to examine the associations between daytime and nighttime light exposures and dementia risk.

In this cohort study, we used objective light data collected under free‐living conditions from a large sample (*n* = 87 577) over a median follow‐up of 8.1 years. The primary purpose was to investigate the associations between personal daytime and nighttime light exposures, measured by wrist‐worn actigraphy devices with built‐in light sensors, and future dementia risk. If significant associations were observed, we planned to rank the predictive value of light exposures against 15 established predictors for dementia^1^, ^16^, ^17^ and conduct mediation analyses to explore potential mediators.

## METHODS

### Study design and participants

This community‐based cohort study was based on the UK Biobank, a large population‐based cohort that enrolled over 500 000 participants aged 40–73 years between 2006 and 2010.^18^

Participants attended 1 of 22 assessment centres across England, Scotland or Wales and underwent detailed baseline assessments, including sociodemographic, lifestyle, health and physical measures. Details of the rationale, design and measurements for the UK Biobank are available online (www.ukbiobank.ac.uk). A total of 236 519 UK Biobank participants were invited to participate in a 7‐day physical activity and light monitoring study. Among them, 106 053 (44.84%) participants agreed to participate and were provided with a wrist‐worn accelerometer (Axivity AX3, Newcastle upon Tyne, UK) with an in‐built light sensor (APDS9007 silicon photodiode sensor).^19^ After data processing and exclusion of invalid recordings, 103 653 participants with valid accelerometer and light data were included. The baseline demographic and health‐related characteristics of the participants who agreed to the measurement were similar to those of the participants who declined.^20^

The sensor was set to activate at 10 a.m., two working days after postal dispatch, to avoid recording during delivery and capture light data over 7 days at 100 Hz, with a peak sensitivity at 560 nm. The devices had an approximately linear response to illuminance between 0 and 5500 lux, as reported in a previous study.^21^ Participants were instructed to wear the device on their dominant wrist while continuing with their usual activities. Participants were asked to mail the device in a prepaid envelope back to the coordinating centres after the monitoring period. The UK Biobank accelerometer expert working group processed the raw accelerometer data (Field ID 90001). Raw acceleration signals were calibrated to gravity. Nonwear time was defined as consecutive stationary episodes lasting at least 1 hour during which all three axes had a standard deviation of less than 13.0 milligravity.^19^ More details about the data processing and analysis have been published.^19^

**FIGURE 1:**
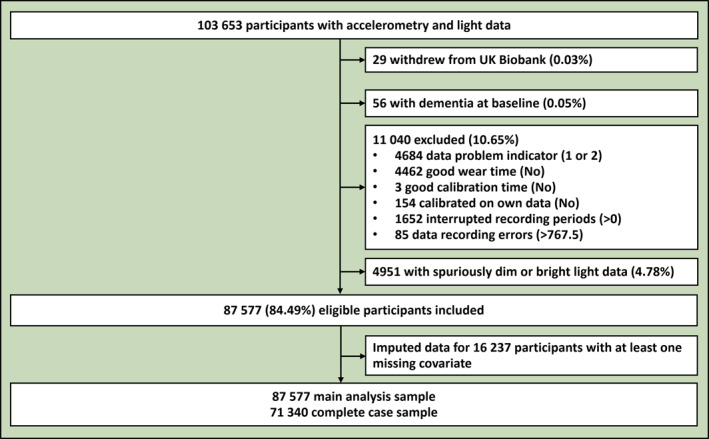
Flowchart of participant enrolment.

The exclusion criteria were as follows: (1) withdrawal from the UK Biobank, (2) dementia at baseline, (3) consistently dark (percentage of data below 10 lux > 80%) or consistently bright (percentage of data above 6000 lux > 75%) data (as this indicated device malfunction or occlusion)^21^ and (4) unreliable or invalid accelerometry data. The criteria for unreliable or invalid accelerometry data included (1) unexpectedly small or large size (Field ID: 90002); (2) recording duration of less than 72 h or failure to provide data for all 1‐h periods within a 24‐h cycle during the 7‐day data collection (Field ID: 90015); (3) poor calibration (Field ID: 90016); (4) recalibrated using the previous accelerometer record from the same device worn by a different participant (Field ID: 90017); (5) data with a nonzero count of interrupted recording periods (Field ID: 90180); and (6) data with more than 767.5 (Q3 + 1.5 × interquartile range) data recording errors (Field ID: 90182). In total, 16 076 participants were excluded. Finally, 87 577 participants (84.49%) with valid data were included in the main analysis using multiple imputation for missing data, whereas 71 340 participants with complete data were included in the sensitivity analysis (figure 1).

### Exposure

As previously reported,^21^ the light measurements showed very good reliability, with an intraclass correlation of 0.82 (95% confidence interval [CI] 0.81–0.83). Validation of the AX3 devices confirmed accurate recovery of true lux after calibration for both low (*r* ^2^ = 0.82) and high (*r* ^2^ = 0.81) light levels. Critically, the 24‐h light‐exposure profile in our sample closely aligned with the canonical pattern established in prior research (Supporting Information S1: figure 1). Our data clearly show two distinct periods of light exposure, and the predefined windows naturally fall into these high and low phases, confirming their appropriateness. This concordance justified our use of the daytime (7:30–20:30) and nighttime (0:30–6:00) light exposure factors that were previously derived from factor analysis.^21^

**FIGURE 2:**
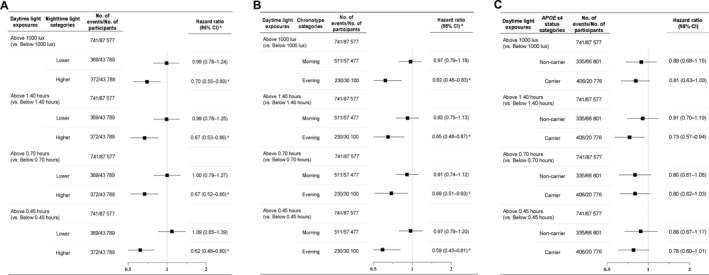
Subgroup analysis of the associations between daytime light exposures and dementia risk stratified by average nighttime light exposure, chronotype and *APOE* ε4 carrier status. (A) Stratified by average nighttime light exposure. *p* values remained significant after multiple testing with the FDR method. (B) Stratified by chronotype. *p* values remained significant after multiple testing with the FDR method. (C) Stratified by *APOE* ε4 carrier status. Error bars represent 95% confidence intervals. Cox proportional hazards regression adjusted for age, sex, ethnicity, Townsend deprivation index, recruitment centre, education level, season of accelerometer wear, photoperiod, PM2.5 absorbance, healthy diet score, vitamin D supplement use, smoking status, alcohol intake, total MVPA volumes, chronotype, social isolation, obesity, diabetes history, hypertension history, traumatic brain injury and hearing loss (Model 4). APOE, apolipoprotein E; CI, confidence interval; FDR, false discovery rate; HR, hazard ratio; MVPA, moderate‐to‐vigorous physical activity.

Average levels of daytime and nighttime light exposure, calculated by averaging light levels across the respective clock time ranges, showed excellent internal consistency (Cronbach's *α* = 0.98 for daytime and *α* = 0.93 for nighttime light).^21^ Based on widely used bright light intensity thresholds^22^, ^23^ and the distribution of daytime light (Supporting Information S1: figure 1), we calculated the daily duration of bright daytime light for each participant at thresholds of 3000, 5000 and 7000 lux. Furthermore, as suggested by the exploratory associations between continuous daytime and nighttime light variables and the risk of dementia (Supporting Information S1: figure 2), continuous nighttime light was converted into quartiles for subsequent analyses, whereas the four continuous daytime light exposure variables were converted into binary variables. The thresholds of 1000 lux, 1.40, 0.70 and 0.45 h used for daytime light conversion were derived from these exploratory associations (Supporting Information S1: figure 2). Details of data processing, quality control and definition of light exposure are presented in Supporting Information S1: Methods.

### Outcome

The outcome was incident dementia, ascertained using primary care, hospital admission and death registry data (Supporting Information S1: table 1). All instances of hospital admission or death were recorded with the International Classification of Diseases 10th Revision codes, and primary care data were recorded using Read Codes Version 2 (Read V2) and Version 3 (Read CTV3) for dementia. Incident dementia events were defined as diagnoses made after the accelerometer measurements. Follow‐up was censored at the date of death or the end of available incident dementia diagnosis (25 January 2024), whichever came first. Dementia identification in UK Biobank has good diagnostic accuracy, with positive predictive values of 86.80%, 87.30% and 80.00% for primary care, hospital admissions and mortality data, respectively, and 82.50% across all datasets.^24^

### Covariates

**FIGURE 3:**
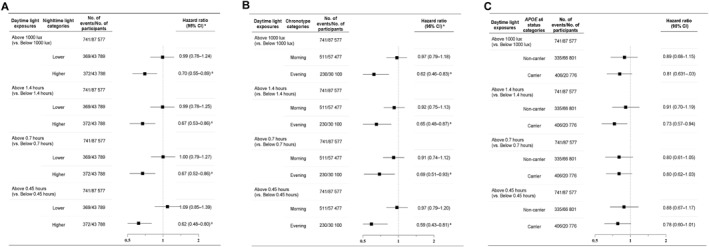
Ranked feature importance by SHAP values for 19 predictive features. Feature importance, calculated using SHAP on the test sets from the 10‐fold cross‐validation, is shown in a summary plot. SHAP values were derived from the XGBoost machine learning model for the prediction of dementia (*n* = 741) versus non‐dementia (*n* = 86 836). SHAP values > 0 indicate a positive impact on prediction while values < 0 indicate a negative impact (e.g., *APOE* ε4 status has a strong positive contribution to the prediction of dementia). The colour represents the scaled feature value (red corresponding to higher values, blue to lower). The position of the *x*‐axis represents the contribution of each normalised feature value to the positive prediction of dementia. APOE, apolipoprotein E; MVPA, moderate‐to‐vigorous physical activity; SHAP, SHapley Additive exPlanations.

Covariate data were obtained from self‐report questionnaires, accelerometers and registry records. Age (continuous) was calculated from the date of birth and the date of accelerometer wear. Sex (female/male), ethnicity (White/others) and recruitment centre (England/Wales/Scotland) were obtained from self‐report questionnaires. The Townsend deprivation index (continuous) was based on the postcode of residence using aggregated data on unemployment, car and home ownership and household overcrowding. Data on the season of accelerometry wear were also recorded (i.e., spring, March to May; summer, June to August; autumn, September to November; and winter, December to February; UK Meteorological Office definitions). Other covariate data, including educational attainment (degree or above/any other qualification/no qualification), smoking status (never/previous/current), frequency of alcohol intake (not current/less than three times a week/three or more times a week) and diet‐related factors, were obtained from touchscreen questions. The healthy diet score was calculated using intake information on vegetables, fruit, fish, unprocessed red meat and processed meat. Photoperiod was derived as the interval between sunrise and sunset on the date of light‐tracking at 53.4808°N, 2.2426°W (Manchester, UK), using ‘getSunlightTimes()’ in the ‘suncalc’ package in R. Vitamin D supplement use (yes or no) was collected from the touchscreen questionnaire. Moderate‐to‐vigorous physical activity (MVPA) was recorded by an accelerometer; details of MVPA data processing are provided in our previous work.^25^ Chronotype was derived from a touchscreen questionnaire response. Obesity status (body mass index ≥ 30 kg/m^2^) was obtained from touchscreen questions. PM2.5 absorbance reflects the concentration of elemental or black carbon in PM2.5. The social isolation index (least/moderate/most) was constructed based on living alone, social contact and participation in social activities, following the validated Berkman–Syme social network index. Histories of traumatic brain injury and hearing loss were ascertained from hospital records. Previous diagnoses of diabetes and hypertension were obtained from self‐report questionnaires, hospital records and death registries. Values for covariates with repeated measurements were obtained from questionnaires at the time point closest to accelerometry (Supporting Information S1: figure 3). Detailed information sources and codes are provided in Supporting Information S1: table 2.

### Potential moderators and mediators

Potential moderators included age, sex, timing of daytime light (defined as over 50% of total daytime light exposure occurring either before or after noon), hypertension, obesity, depression, diabetes, chronotype and *apolipoprotein E (APOE)* ε4 status. In particular, previous evidence suggests that light exposure history modifies the impact of nighttime light stimuli on circadian rhythm biomarkers.^26^ We also examined the interaction between daytime and nighttime light exposures. In addition, hypothesised mediators included circadian rest–activity rhythms (CRARs), brain structures and serum vitamin D concentration because of their associations with both cognition and light exposures.^16^, ^27^, ^28^, ^29^ Details are provided in Supporting Information S1: Methods.

### Statistical analyses

**FIGURE 4:**
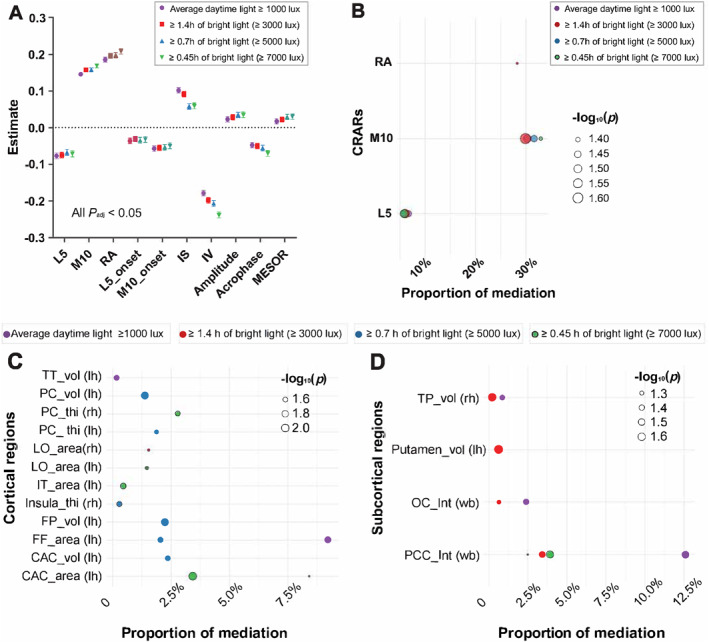
Mediation effects of CRARs and brain structures on the associations between binary daytime light exposures and dementia risk. (A) Associations between circadian rest–activity rhythms (CRARs) and daytime light exposures. (B) Mediation effects of CRARs on the association between daytime light exposures and dementia risk. (C) The proportion of mediation of cortical regions on the association between four daytime light exposures and dementia risk; (D) The proportion of mediation of subcortical regions on the association between four daytime light exposures and dementia risk. Each colour represents a daytime light exposure, with the size of the circles indicating the −log10(*p*) value. Analyses were adjusted for age, sex, ethnicity, Townsend deprivation index, recruitment centre, education level, season of accelerometer wear, photoperiod, PM2.5 absorbance, healthy diet score, vitamin D supplement use, smoking status, alcohol intake, MVPA, chronotype, social isolation index, obesity, history of diabetes, hypertension, traumatic brain injury and hearing loss. Acrophase, the peak time of the rest–activity rhythm; Amplitude, the amplitude of the rest–activity rhythm; IS, interdaily stability; IV, intradaily variability; L5 onset, the onset time point of L5; L5, the average activity during the least active 5‐h period; M10 onset, the onset time point of M10; M10, the average activity during the most active 10‐h period; MESOR, the midline estimating statistic of rhythm; MVPA, moderate‐to‐vigorous physical activity; RA, relative amplitude. The cortical and subcortical regions are listed as follows: TT_vol: the volume of transverse temporal cortex; PC_vol: the volume of posterior cingulate cortex; PC_thi: the mean thickness of posterior cingulate cortex; LO_area: the area of lateral occipital cortex; IT_area: the area of inferior temporal cortex; Insula_thi: the mean thickness of insula; FP_vol: the volume of frontal pole cortex; FF_area: the area of fusiform; CAC_vol: the volume of caudal anterior cingulate cortex; CAC_area: the area of caudal anterior cingulate cortex. TP_vol: the volume of thalamus‐proper; Putamen_vol: the volume of putamen; OC_Int: the mean intensity of optic chiasm; PCC_Int: the mean intensity of the posterior corpus callosum; lh: left hemisphere; rh: right hemisphere; wb: whole brain.

The number of dementia events was sufficient according to the rule of thumb of at least 10 events per variable.^30^ Detailed information on missing data patterns of covariates is presented in Supporting Information S1: figure 4 and table 3. Multiple imputation was used to handle missing data.^31^ The overall sample and complete case sample presented similar baseline characteristics (Supporting Information S1: table 4).

**FIGURE 5:**
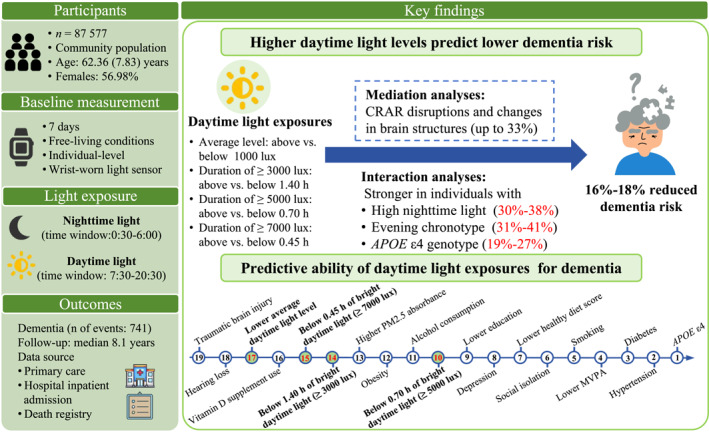
The study design and key findings on associations between wearable‐device‐measured daytime and nighttime light exposures and dementia risk. Higher daytime light levels predict lower dementia risk and have a predictive ability for dementia. APOE, apolipoprotein E; CRAR, circadian rest–activity rhythm; MVPA, moderate‐to‐vigorous physical activity.

Restricted cubic spline models were used to visualise dose–response relationships between continuous light exposures and dementia.^32^ Cox regression was used to examine the associations between binary daytime light exposures and dementia. Hazard ratios (HRs) and their 95% confidence intervals (CIs) were calculated. Model 1 was adjusted for age and sex, which represent fundamental demographic confounders. Model 2 additionally included ethnicity, Townsend deprivation index, recruitment centre, education level, season of accelerometer wear, photoperiod and PM2.5 absorbance to account for sociodemographic and environmental influences, given that these factors may affect both habitual light exposure and dementia risk. Model 3 was further adjusted for healthy diet score, vitamin D supplement use, smoking status, alcohol intake, MVPA, chronotype and social isolation index, which are health behaviours and social factors potentially influencing both light exposure and dementia risk. Model 4 was further adjusted for obesity, history of diabetes, hypertension, traumatic brain injury and hearing loss, as these clinical conditions are known dementia risk factors. Proportional hazards assumptions were satisfied, as indicated by Schoenfeld residual plots showing no significant nonzero slopes and by parallel cumulative risk curves (Supporting Information S1: figures 5 and 6). Collinearity between covariates was examined via correlation matrix analysis (Supporting Information S1: table 5), which revealed no evidence of multicollinearity.

We performed several sensitivity analyses: using Fine‒Gray subdistribution hazards model by incorporating all‐cause death as a competing risk^33^; using a complete case sample; controlling for month of accelerometer wear instead of season; excluding participants with shift work history, visual disturbances or blindness; using a sample with ≥ 6 days of light data; controlling for *APOE* ε4 carrier status, depression and daily outdoor duration; and using photoperiod ‐defined daytime light exposure.

Interaction analyses were conducted on levels of nighttime light exposure, chronotype, *APOE* ε4 carrier status, age, sex, timing of daytime light exposure and depression. The XGBoost method, an extreme gradient boosting tree algorithm, was used to construct the machine learning model.^34^ XGBoost is renowned for its strong performance and high computational efficiency, particularly when handling large‐scale samples. Based on decision tree methodology, XGBoost enables the calculation of the importance of each variable.^34^ The importance score for an individual decision tree is calculated based on the performance improvement attributed to each variable's splitting point, as measured by the Gini index. Following the construction of the tree, the model undergoes gradient boosting, which yields adjusted importance scores for each variable. To ensure optimal performance, a 10‐fold cross‐validation was employed for parameter selection. Additionally, we used the SHapley Additive exPlanations (SHAP) method^35^, ^36^ to determine the relative importance of 19 predictors for dementia risk. SHAP is a widely recognised technique for enhancing the interpretability of machine learning models, applying a game theory approach to allocate fair payouts to players based on their contributions to the total gain.^36^ In the context of model prediction, this translates to assigning quantitative importance values to variables based on their contributions to the prediction. Higher contributions result in higher SHAP scores. Therefore, the SHAP value is defined as the average marginal contribution of a variable across all possible variable coalitions.^36^ Potential mediation effects of CRARs, brain structures (with 16 855 participants) and serum vitamin D concentration were investigated. Mediation proportions were reported, with *p*‐values calculated through quasi‐Bayesian approximation using 5000 simulations.

All statistical analyses were performed using the R software V.4.2.2 (R Development Core Team, Vienna, Austria). All statistical tests were two‐sided, and a *p* value of < 0.05 was considered statistically significant. The *p* values in fully adjusted models were corrected using false discovery rate (FDR), with uncorrected results considered exploratory.^37^

## RESULTS

### Baseline characteristics

Over a median follow‐up of 8.1 years (699 815 person‐years), 741 (0.85%) participants developed dementia. Baseline characteristics of the 87 577 participants stratified by incident dementia status are shown in Supporting Information S1: table 6. Those who developed dementia were generally older, more likely to be male, less educated, had lower MVPA levels and were more likely to be smokers or to have a history of diabetes, hypertension or hearing loss. Baseline characteristics stratified by daytime light exposure are shown in Supporting Information S1: table 7.

### Daytime and nighttime light exposures and dementia risk

Supporting Information S1: figure 2A showed associations between continuous daytime light exposure and dementia risk, with a key threshold at 1000 lux. Below this level, the fully adjusted HR was above 1, indicating higher risk, whereas above 1000 lux, HR decreased to approximately 0.75, suggesting lower risk. Supporting Information S1: figure 2B–D depicted the associations of bright daytime light duration (≥ 3000, ≥ 5000 and ≥ 7000 lux) with dementia risk. Significant thresholds were identified at 1.40 h (≥ 3000 lux), 0.70 h (≥ 5000 lux) and 0.45 h (≥ 7000 lux), with HR decreasing to around 0.75 as exposure increased. Supporting Information S1: figure 2E showed no apparent association between continuous nighttime light and dementia risk.

As shown in table 1, participants exposed to an average light level above 1000 lux had a significantly lower dementia risk compared with those below this threshold (adjusted HR = 0.84, 95% CI 0.71–0.99, *p* *=* 0.039). Longer bright daytime light durations were associated with reduced dementia risk across all three intensity thresholds: above 1.40 h at ≥ 3000 lux (0.82, 95% CI 0.70–0.97, *p* = 0.024), above 0.70 h at ≥ 5000 lux (0.83, 95% CI 0.70–0.99, *p* = 0.036) and above 0.45 h at ≥ 7000 lux (0.83, 95% CI 0.70–0.99, *p* *=* 0.041). These associations remained significant after multiple testing corrections (FDR‐adjusted *p* < 0.05). Cumulative risk plots corroborated these results, depicting lower dementia risk with higher levels of daytime light exposure (Supporting Information S1: figure 6A–D). However, no significant associations were observed between quartiles of nighttime light exposure and dementia risk (table 1, Supporting Information S1: figure 6E). Therefore, subsequent analyses including sensitivity, interaction, subgroup, comparative and mediation analyses were conducted only for daytime light exposures.

**TABLE 1:** Associations of daytime light exposure measures and average nighttime light exposures with dementia risk

Light exposures	Events/*n*	Model 1 HR (95% CI); *p*	Model 2 HR (95% CI); *p*	Model 3 HR (95% CI); *p*	Model 4 HR (95% CI); *p* ^a^
Average daytime light	741/87 577				
Below 1000 lux	353/40 553	1.00 (reference)	1.00 (reference)	1.00 (reference)	1.00 (reference)
Above 1000 lux	388/47 024	0.87 (0.76–1.01); 0.062	0.79 (0.67–0.94); 0.007	0.83 (0.71–0.98); 0.032	0.84 (0.71–0.99); 0.039^a^
Duration of bright light (≥ 3000 lux)	741/87 577				
Below 1.40 h	357/40 970	1.00 (reference)	1.00 (reference)	1.00 (reference)	1.00 (reference)
Above 1.40 h	384/46 607	0.86 (0.74–0.99); 0.036	0.78 (0.66–0.92); 0.003	0.82 (0.69–0.97); 0.019	0.82 (0.70–0.97); 0.024^a^
Duration of bright light (≥ 5000 lux)	741/87 577				
Below 0.70 h	324/37 088	1.00 (reference)	1.00 (reference)	1.00 (reference)	1.00 (reference)
Above 0.70 h	417/50 489	0.87 (0.75–1.00); 0.055	0.78 (0.66–0.93); 0.005	0.83 (0.70–0.98); 0.029	0.83 (0.70–0.99); 0.036^a^
Duration of bright light (≥ 7000 lux)	741/87 577				
Below 0.45 h	391/44 820	1.00 (reference)	1.00 (reference)	1.00 (reference)	1.00 (reference)
Above 0.45 h	350/42 757	0.88 (0.76–1.02); 0.080	0.77 (0.65–0.92); 0.004	0.82 (0.69–0.98); 0.032	0.83 (0.70–0.99); 0.041^a^
Average nighttime light	741/87 577				
Q1 (median 0.89 lux)	185/21 895	1.00 (reference)	1.00 (reference)	1.00 (reference)	1.00 (reference)
Q2 (median 2.30 lux)	184/21 894	1.06 (0.86–1.30); 0.584	1.05 (0.85–1.29); 0.650	1.03 (0.84–1.26); 0.809	1.02 (0.83–1.25); 0.847
Q3 (median 12.61 lux)	187/21 894	1.06 (0.86–1.30); 0.596	1.04 (0.85–1.28); 0.665	1.03 (0.84–1.26); 0.805	1.02 (0.83–1.25); 0.863
Q4 (median 80.80 lux)	185/21 894	1.00 (0.81–1.23); 0.993	0.98 (0.80–1.21); 0.864	0.96 (0.78–1.18); 0.707	0.96 (0.78–1.18); 0.676

*Note:* The average daytime light level was dichotomised into binary categories, defined by a 1000 lux threshold and the durations of bright daytime light above 3000 lux, 5000 lux and 7000 lux were dichotomised into binary categories using thresholds of 1.40, 0.70 and 0.45 h, respectively, for analysis. This categorisation was identified from the exploratory analysis (Supporting Information S1: figure 2A–D). The average nighttime light was categorised into quartile‐based groups using the 25th, 50th and 75th percentiles to comprehensively examine its distribution and association with dementia risk. Cox proportional hazards regression was used to examine the associations. Model 1 was adjusted for age and sex. Model 2 was adjusted as in Model 1 and for ethnicity, Townsend deprivation index, recruitment centre, education level, season of accelerometer wear, photoperiod and PM2.5 absorbance. Model 3 was adjusted as in Model 2 and for healthy diet score, vitamin D supplement use, smoking status, alcohol intake, total MVPA volumes, chronotype and social isolation. Model 4 was adjusted as in Model 3 and for obesity, diabetes history, hypertension history, traumatic brain injury and hearing loss.

Abbreviations: CI, confidence interval; FDR, false discovery rate; HR, hazard ratio; MVPA, moderate‐to‐vigorous physical activity; Q, quartile.

^a^ *p* values remained significant after multiple testing with the FDR method.

### Sensitivity analyses on the association between daytime light exposures and dementia risk

Overall, the main results for daytime light exposures (table 1) were robust in sensitivity analyses (Supporting Information S1: tables 8–14), including controlling for the month of accelerometer wear, using a subsample with ≥ 6 days of accelerometer wear (Supporting Information S1: tables 8 and 9), using a competing risk regression model, using the dataset without imputation, excluding participants with shift work history, visual disturbances or blindness, additionally adjusting for *APOE* ε4 carrier status, depression and daily outdoor duration and using photoperiod‐defined daytime light, although some associations became slightly attenuated and no longer reached statistical significance in the latter analyses.

### Interaction and subgroup analyses on the association between daytime light exposures and dementia risk

Significant interactions were observed between the four daytime light exposures and levels of average nighttime light exposure, chronotype and *APOE* ε4 carrier status (Supporting Information S1: tables 15–17), whereas no significant interactions were found for age, sex, timing of daytime light and depression (Supporting Information S1: tables 18–21). Subgroup analyses revealed that the beneficial associations of higher average daytime light levels and longer bright daytime light durations with reduced dementia risk were more prominent among individuals with higher levels of average nighttime light exposure (figure 2A and Supporting Information S1: table 22), individuals with an evening chronotype (figure 2B and Supporting Information S1: table 23) and *APOE* ε4 carriers (figure 2C and Supporting Information S1: table 24). Specifically, higher daytime light exposure was associated with a 30%–38% reduction in dementia risk among those with higher nighttime light levels, a 31%–41% reduction among evening chronotypes and a 19%–27% reduction among *APOE* ε4 carriers.

### Comparative analyses on the association between daytime light exposures and dementia risk

We compared the predictive ability of four daytime light exposure measures with 15 established predictors of dementia. Among these 19 factors, below 0.70 h of bright daytime light (≥ 5000 lux) ranked 10th, positioned between lower education (9th) and alcohol consumption (11th). Below 1.40 h of bright daytime light (≥ 3000 lux) and below 0.45 h (≥ 7000 lux) ranked 14th and 15th, respectively, exceeding vitamin D supplement use, hearing loss and traumatic brain injury, whereas lower average daytime light level ranked 18th (figure 3).

### Mediation effects on the association between daytime light exposures and dementia risk

Before mediation analyses, linear regression analysis was applied to illustrate associations of binary daytime light exposures with potential mediators (figure 4A, Supporting Information S1: figure 7A–H and tables 25–26). In exploratory analyses, L5 and M10 significantly mediated 5.70%–32.99% (all *p* < 0.05) of the associations between four daytime light exposures and dementia risk and relative amplitude mediated 28.24% (*p* = 0.042) of the association between the duration above 1.40 h of bright daytime light (≥ 3000 lux) and dementia (figure 4B and Supporting Information S1: table 27), although these mediation effects became statistically nonsignificant after FDR correction. The fusiform cortex area significantly mediated the association between average daytime light above 1000 lux and dementia risk (9.20%; *p* = 0.018), and this association remained significant after FDR correction (figure 4C and Supporting Information S1: table 28). In addition, exploratory mediation effects were observed for the mean intensity of posterior corpus callosum (12.61%; *p* = 0.029) and the caudal anterior cingulate cortex area (8.40%; *p* = 0.038), although these associations did not survive FDR correction (figure 4C, D and Supporting Information S1: table 28). Notably, no significant mediation effects of serum vitamin D level were found (Supporting Information S1: table 29).

## DISCUSSION

### Main findings

In this prospective cohort study of 87 577 UK Biobank participants, higher daytime light exposure—both in average level (above 1000 lux) and bright‐light duration (e.g., ≥ 0.70 h at ≥ 5000 lux)—measured under free‐living conditions was significantly associated with a lower dementia risk. This association was particularly evident for individuals with higher nighttime light exposure, evening chronotype or *APOE* ε4 carrier status. Notably, the predictive value of < 0.70 h of bright daytime light (≥ 5000 lux) exceeded that of six traditional risk factors, including alcohol consumption, obesity, air pollution and hearing loss (figure 5). However, no significant association between nighttime light exposure and dementia risk was identified.

No prior study has investigated associations between average daytime and nighttime light exposures and dementia risk. In our study, individuals exposed to average daytime light levels exceeding 1000 lux in late middle to older age had an approximately 16% reduced risk of dementia compared with those with daytime light levels below 1000 lux. This finding is supported by previous evidence linking daytime light to factors associated with dementia.^21^, ^38^ Previous studies suggest that brighter daytime light intensity (≥ 1000 lux) can improve circadian rhythms.^38^, ^39^, ^40^ A recent large‐scale cohort study involving more than 8500 individuals demonstrated that the wearable‐device‐measured average daytime light level of approximately 1380 lux predicted lower risks of mental disorders, including major depressive disorder,^21^ a risk factor for dementia.^1^, ^3^, ^41^ In addition, a case–control study (*n* = 77) found that patients with severe dementia spent significantly less time exposed to daytime light (≥ 1000 lux) than those with mild or no dementia.^38^ Therefore, ensuring brighter daytime light exposure (e.g., ≥ 1000 lux) may be a simple means of preventing psychiatric disorders and dementia. This is particularly relevant given that people in modern society spend nearly 90% of their day indoors under artificial lighting,^14^ which typically provides insufficient brightness (∼300–500 lux) during the day.

Given the growing evidence that BLT can ameliorate circadian disruptions,^12^, ^42^ mental disorders^22^, ^23^, ^43^ and neurodegenerative diseases,^9^, ^13^ we further explored the relationship between the duration of bright daytime light and dementia risk. For example, participants with exposure to light of at least 7000 lux for more than 0.45 h had a 17% lower dementia risk than those with shorter exposure durations. These findings are broadly consistent with those of previous randomised controlled trials,^23^, ^43^, ^44^, ^45^ which demonstrated the efficacy of BLT (typically 0.5–2.0 h) in treating depression,^23^, ^43^ Parkinson's disease^44^, ^46^ and dementia.^13^, ^45^ However, a cohort study reported a J‐shaped relationship between outdoor sunlight duration and dementia risk, with a 1.2‐fold higher dementia risk when the duration of outdoor sunlight exceeded 1.5 h.^29^ Differences in light measurement may account for this discrepancy. That study relied on self‐reported outdoor time in summer and winter to estimate the duration of sunlight exposure, which may not be a reliable proxy for daytime bright light exposure. In contrast, we used objective measurements under free‐living conditions (both indoors and outdoors), continuously monitoring daylight exposure over 7 days. In addition, participants in the UK Biobank with longer outdoor exposure had lower educational levels,^28^ which may partially confound the relationship between outdoor time and dementia risk. Furthermore, the predictive value of longer bright daytime light duration for dementia risk exceeded that of well‐established predictors such as obesity, air pollution and traumatic brain injury.

The lack of a significant association between nighttime light exposure and dementia risk in our study contrasts with some prior cross‐sectional studies.^47^, ^48^ A key methodological challenge in this field is the accurate measurement of personal, retinally relevant light exposure, which may help explain the discrepant findings. First, wrist‐worn light sensors, although practical for long‐term monitoring, likely underestimate true retinal light exposure as they may miss directional sources such as handheld screens (e.g., smartphones).^21^ Second, the devices used in this study did not capture spectral composition, notably the blue‐enriched emission of modern light‐emitting diodes (LEDs), which is most potent in causing circadian disruption. Such nondifferential misclassification would attenuate any true association. Third, our light data were collected between 2014 and 2018, preceding the widespread nighttime use of personal electronic devices common today. The observed exposure may therefore not reflect current patterns of nocturnal light exposure, which are likely more intense and frequent. Future studies should incorporate ocular‐level light monitoring, spectral characterisation and more recent exposure data to clarify this relationship.

Several mechanisms may underlie the protective association between daytime light exposure and dementia. Circadian disruptions are recognised as risk factors for neurodegenerative diseases, including dementia.^9^, ^49^, ^50^ Daytime light exposure or therapy could ameliorate circadian rhythm disturbances^12^, ^13^ and increase nocturnal melatonin levels.^51^ Our exploratory mediation analyses involving CRAR data offer preliminary support for the hypothesis that improvements in circadian rhythms may have contributed to the observed protective association. Previous imaging studies have linked dementia to atrophy in multiple brain regions, including frontal,^52^, ^53^ occipital and temporal lobes,^54^ as well as reductions in volumes of putamen, thalamus and corpus callosum.^55^, ^56^, ^57^, ^58^ Interestingly, exploratory mediation analyses suggested that adequate daytime light exposure may mitigate area reductions in occipital and frontotemporal cortices, volumetric decreases in putamen and thalamus, as well as intensity reductions in posterior corpus callosum, thereby potentially offering another protective mechanism against dementia progression. However, these exploratory associations require verification in future studies. Moreover, daytime light exposure may favourably modulate the pathogenesis of dementia.^59^ Light therapy has been found to alleviate neuroinflammation,^60^, ^61^ synaptic transmission irregularities,^60^, ^62^ and mitochondrial dysfunction.^62^

Another novel finding is the more pronounced protective association among individuals with higher average nighttime light exposure, an evening chronotype or *APOE* ε4 carrier status, showing a risk reduction of up to 41%. Previous studies demonstrated adaptive circadian photoreception, whereby prior light exposure modulates the sensitivity of the circadian system to subsequent light stimuli.^26^, ^63^, ^64^ Such adaptive mechanisms may relate to the observed interaction between daytime and nighttime light exposures in relation to dementia risk. In addition, previous studies have revealed similar gene–environment interactions, showing that late‐life physical inactivity and circadian disruptions were more strongly associated with dementia risk in *APOE* ε4 carriers.^27^, ^65^ The *APOE* ε4 allele is associated with medial temporal lobe atrophy in older adults.^66^, ^67^ In our study, high daytime light levels were associated with increased surface area or volume in the medial temporal lobe, possibly contributing to the observed interaction effects. In addition, *APOE* ε4 accelerates amyloid β (Aβ) aggregation, a key pathology in dementia,^68^ whereas light therapy markedly reduces Aβ deposition in mice.^69^ Thus, we hypothesise that high daytime light levels may synergistically reduce Aβ deposition in *APOE* ε4 carriers. To date, the relationship between evening chronotype and dementia remains debated,^70^, ^71^ and the mechanisms underlying chronotype–light synergistic effects remain largely unclear, warranting further investigation.

### Limitations

First, the UK Biobank sample is healthier and less socioeconomically deprived than the general population, possibly limiting the generalisability of risk estimates, though exposure–outcome relationships remain widely applicable.^72^ Importantly, although the direction of the association is likely robust, the specific light exposure thresholds identified (e.g., ≥ 1000 lux) may not be directly transferable to populations with different health profiles or lifestyles. Future studies are needed to validate and potentially calibrate these thresholds in more diverse populations. Second, although 7‐day light monitoring is reliable for capturing weekly patterns,^21^ it may not fully reflect long‐term behaviour. Third, the levels of light exposure were measured at the wrist rather than at the ocular level. Given that light exerts its primary nonvisual health‐modulating effects via ocular pathways, the wrist‐worn proxy data provide a coarse estimate of the actual effects of light on dementia. Future studies using ocular‐level measurements are needed to establish biologically relevant thresholds. Fourth, the device cannot distinguish true darkness from being covered, potentially leading to underestimation, despite efforts to exclude biased data. Fifth, reported lux values were validated 8 years later, with high accuracy. Sixth, certain mediation effects became nonsignificant following FDR adjustment, indicating that these results should be regarded as exploratory. Seventh, some covariates were collected 5.6 years before baseline, but their stability over time^73^ suggests this does not weaken the findings. Lastly, light exposure data were collected between 2014 and 2018 before the widespread adoption of LED lighting and increased nighttime device use. Since then, both the intensity and spectral composition of typical light exposure have changed. Thus, the observed associations may not directly reflect current exposure patterns and require validation using more recent data.

### Implications

Our findings have several implications. First, they suggest the importance of higher daytime light exposure in reducing dementia risk, which appears to exceed many traditional predictors. Free‐living light exposure can also be easily measured objectively, potentially providing a promising method to identify individuals at high risk of dementia. Second, increasing daytime light exposure may be a simple and cost‐free strategy to reduce dementia risk in both clinical and community settings. Practical implementation pathways could include optimising indoor lighting at home, community‐based outdoor activity promotion programmes and workplace lighting modifications designed to increase daytime light exposure, such as ensuring adequate illumination and access to natural light. This study suggests several protective thresholds of daytime light exposures, which may facilitate the design and evaluation of future interventional studies. Third, our findings underscore a more pronounced protective association of daytime light exposure in individuals with higher average nighttime light exposure, an evening chronotype or *APOE* ε4 carrier status. In other words, these findings suggest a targeted approach to mitigate dementia risk by increasing daytime light levels for these populations. Future investigations are warranted to confirm the protective effects of daytime light on dementia risk.

## AUTHOR CONTRIBUTIONS

Jihui Zhang and Hongliang Feng had full access to all the data in the study and take responsibility for the integrity of the data and the accuracy of the data analysis. Concept and design: Jihui Zhang, Hongliang Feng, Nana Zheng and Wei Wang. Acquisition of data: Xionge Mei, Yue Liu and Jing Du. Analysis and interpretation of data: Jihui Zhang, Hongliang Feng, Nana Zheng, Wei Wang and Biao Li. Drafting of the manuscript: Nana Zheng, Wei Wang, Biao Li and Hongliang Feng. Critical revision of the manuscript for important intellectual content: Ngan Yin Chan, Joey W. Y. Chan, Xiaoman Xing, Xiao Tan, Christian Benedict and Yun Kwok Wing. Statistical analysis: Nana Zheng, Wei Wang and Biao Li. Obtained funding: Hongliang Feng, Jihui Zhang and Christian Benedict. Supervision: Jihui Zhang and Hongliang Feng. All authors read and approved the final manuscript. Jihui Zhang and Hongliang Feng are the guarantors.

## FUNDING

Hongliang Feng was funded by the National Key R&D Programme of China (2022ZD0214100), the National Natural Science Foundation of China (82571696), Guangzhou Municipal School (College)‐Enterprise Joint Funding Project (2024A03J0214) and Guangzhou Science and Technology Plan Project (2025A03J3929). Jihui Zhang was funded by the National Natural Science Foundation of China (82341240 and 82171476). Christian Benedict was funded by the Novo Nordisk Foundation (NNF23OC0081873) and the Swedish Brain Research Foundation (FO2023‐0292). This work was supported by Guangzhou Key Clinical Speciality (Clinical Medical Research Institute) and the Guangzhou Municipal Key Discipline in Medicine Project (2025–2027). This research has been conducted using the UK Biobank Resource under Application Number 58082. The UK Biobank was supported by the Wellcome Trust, Medical Research Council, Department of Health, Scottish Government and Northwest Regional Development Agency. It has also had funding from the Welsh Government, British Heart Foundation, Cancer Research UK and Diabetes UK.

## CONFLICT OF INTEREST STATEMENT

Yun Kwok Wing reports grants from Research Grant Council General Research Fund (GRF) and Collaborative Research Fund (CRF) and Health and Medical Research Fund (HMRF), which are unrelated to the submitted work. Yun Kwok Wing delivered a lecture for Hong Kong Society of Psychiatrists (unrelated to the submitted work) and received an honorarium for serving as the Chair of the 3rd Asian Narcolepsy & Hypersomnolence Society Meeting which is outside the submitted work. Joey W. Y. Chan received travel support from Lundbeck HK Limited for an overseas conference, which is unrelated to the submitted work. All other authors declare no competing interests.

## ETHICS STATEMENT

The UK Biobank received ethical approval from the North West Multicentre Research Ethics Committee to collect and distribute samples and data from the participants (Reference numbers: 16/NW/0274 and 21/NW/0157). All UK Biobank participants provided informed consent.

## CONSENT FOR PUBLICATION

Not applicable.

## Supporting information

Supporting Information S1

## Data Availability

All data supporting the UKB study were obtained from the UK Biobank. The use of UK Biobank data has been approved by the UK Biobank Research Team (Application ID: 58082). Researchers who wish to access the data must follow the official procedure for data application from the UK Biobank (https://ams.ukbiobank.ac.uk/ams/).
