# Supplementary material for: Associations between wearable‐device‐measured daytime and nighttime light exposures and dementia risk: A prospective cohort study
Source: Gen Psychiatr. 2026 Jun 24;39(3):e70039. doi: 10.1002/gps3.70039 (PMC13291554; doi:10.1002/gps3.70039)
Supplement: Supplementary file 1 — Supporting Information S1 [file GPS3-39-e70039-s001.docx]

**Supplementary material**

Associations between wearable-device-measured daytime and nighttime light exposures and dementia risk: A prospective cohort study

[Supplementary Methods 3](#_Toc32698)

[Supplemental figure 1 The 24-hour light-exposure profile 5](#_Toc7313)

[Supplemental figure 2 Association between daytime and nighttime light exposure (continuous variables) and dementia risk 6](#_Toc13398)

[Supplemental figure 3 Timeline of some covariates collection 7](#_Toc4951)

[Supplemental figure 4 The missing data pattern of the covariates 8](#_Toc28628)

[Supplemental figure 5 Scaled Schoenfeld residual plots for dementia risk 9](#_Toc26768)

[Supplemental figure 6 The cumulative risk curves for dementia based on the categories of daytime light exposures 10](#_Toc9139)

[Supplemental figure 7 Associations between daytime light exposures and cortical structures 11](#_Toc29968)

[Supplemental table 1. The information sources and codes of outcomes 12](#_Toc21053)

[Supplemental table 2. The information sources and codes of covariates 13](#_Toc14021)

[Supplemental table 3. The numbers (percentages) of participants with missing covariates 14](#_Toc29747)

[Supplemental table 4. Baseline characteristics of overall sample and complete case sample 15](#_Toc7495)

[Supplemental table 5. Pearson correlation coefficient matrix of variables included in the fully adjusted model 16](#_Toc22175)

[Supplemental table 6. Baseline characteristics of the study participants by incident dementia status 17](#_Toc17097)

[Supplemental table 7. Baseline characteristics of the study participants by average daytime light 18](#_Toc21478)

[Supplemental table 8. Sensitivity analysis on the associations between daytime light exposure and dementia risk by controlling for month of accelerometer wear 19](#_Toc21280)

[Supplemental table 9. Sensitivity analysis on the associations between daytime light exposure and dementia risk by using the subsample with ≥6 days of accelerometer wear 20](#_Toc21716)

[Supplemental table 10. Sensitivity analysis on the associations between daytime light exposure and dementia risk by using competing risk regression (Fine and Gray) 21](#_Toc6754)

[Supplemental table 11. Sensitivity analysis on the associations between daytime light exposure and dementia risk by using the dataset without imputation 22](#_Toc551)

[Supplemental table 12. Sensitivity analysis on the associations daytime light exposure and dementia risk by excluding participants with shift work history,visual disturbances or blindness 23](#_Toc6205)

[Supplemental table 13. Sensitivity analysis on the associations between daytime light exposure and dementia risk by additionally adjusting for](#_Toc18872) *[APOE](#_Toc18872)* [ε4 carrier status, depression, and daily outdoor duration 24](#_Toc18872)

[Supplemental table 14. Sensitivity analysis on the associations between photoperiod-defined daytime light exposure and dementia risk 25](#_Toc1601)

[Supplemental table 15. Interaction effects of daytime light exposure and average nighttime light on dementia risk 26](#_Toc6445)

[Supplemental table 16. Interaction effects of daytime light exposure and chronotype on dementia risk 27](#_Toc5250)

[Supplemental table 17. Interaction effects of daytime light exposure and](#_Toc7463) *[APOE](#_Toc7463)* [ε4 status on dementia risk 28](#_Toc7463)

[Supplemental table 18. Interaction effects of daytime light exposure and age categories (< 65 and ≥ 65 years) on dementia risk 29](#_Toc3706)

[Supplemental table 19. Interaction effects of daytime light exposure and sex on dementia risk 30](#_Toc11190)

[Supplemental table 20. Interaction effects of daytime light exposure and the timing of daytime light exposure on dementia risk 31](#_Toc13339)

[Supplemental table 21. Interaction effects of daytime light exposure and depression on dementia risk 32](#_Toc4914)

[Supplemental table 22. Subgroup analysis on the associations between daytime light exposures and dementia risk stratified by average nighttime light level 33](#_Toc24203)

[Supplemental table 23. Subgroup analysis on the associations between daytime light exposures and dementia risk stratified by chronotype 34](#_Toc3667)

[Supplemental table 24. Subgroup analysis on the associations between daytime light exposures and dementia risk stratified by](#_Toc9844) *[APOE](#_Toc9844)* [ε4](#_Toc9844)[status 35](#_Toc9844)

[Supplemental table 25. Associations between daytime light exposures (binary variables) and CRARs 36](#_Toc4309)

[Supplemental table 26. Associations between daytime light exposures (binary variables) and brain structure 37](#_Toc32208)

[Supplemental table 27. Mediation analysis of associations between daytime light exposures (binary variables) and CRARs 45](#_Toc9529)

[Supplemental table 28. Mediation analysis of associations between daytime light exposures (binary variables), brain structure and dementia risk 46](#_Toc19427)

[Supplemental table 29. Associations and mediation analysis of associations between daytime light exposures (binary variables) and serum vitamin D levels 48](#_Toc2406)

# **Supplementary methods**

**Light analysis**

Epochs representing non-wear time were imputed based on all wear time data at a similar time of day on different days for each participant. The accelerometer also generated physical activity intensity data (average vector magnitude in milligravity units) in 5-s epochs (field ID 90004). Participant light data (n=103 653) were analysed with Python scripts by PyCharm software (Community Edition 2023.3.4). The timestamps and light data were extracted from the .cwa files, and timestamps were converted to a standardised format with time zone adjustment. The output current of the light was converted into approximate lux based on the AX3 manual by the function: lux=10^(output current/341). A zero correction was applied to the raw lux values.

Then, the zero-corrected lux values were calibrated by the formula^1^:

$Calibrated lux=\left\{ \begin{aligned} 21.55x-4.54x^{2}, x\leq2 \\ 10.67x, x>2 \end{aligned} \right.$

The converted data calibrated in lux were sampled into 5-second epochs using the median values.

The calibrated light data were then imputed by the imputed value in the acceleration intensity time series data (filed ID 90004). Data were matched with the same eids and timestamps, and missing values were imputed in cases where the imputed flag was set to 1. Missing data points were filled using the averaged valid data from other days to ensure that imputation reflected typical values for that specific missing time interval. If data were missing across all days, the average value from the closest available time interval over a 7-day period would be used to impute.

Only the matched and imputed data were used for further analysis. Participants with a percentage of missing values >28.5% (totally over 48 hours per 7 days) were excluded. The data below 10 lux with a percentage >80% or above 6000 lux with a percentage >75% were considered consistent dark or consistent bright, which were excluded according to Burns's methods. The calibrated light data were averaged into forty-eight 30-min bins across the 24-hour cycle over seven days.

**Potential moderators**

Genotyping was performed with two closely related arrays, and quality control and imputation were conducted on all genetic data. Further information on the processing of UKB genetic data has been published elsewhere^2^. *APOE* ε4 status was defined using directly genotyped SNPs (rs429358 and rs7412). ε1/ε3 and ε2/ε4 genotypes were excluded because they were not able to be distinguished from the unphased genotype data. In analyses with *APOE* ε4 status, we restricted the sample to individuals of European ancestry and additionally adjusted for the top five principal components of ancestry^3^. Previous diagnoses of depression were obtained from the self-reported questionnaires (field ID codes: 20123/20124/20125), hospital records and death registry (ICD 10 codes: F32/33). The timing of daytime light exposure was defined as whether over 50% of total daytime light occurred between 7:30 am and 12:00 pm or between 12:00 pm and 8:30 pm. Other potential moderators included age, sex and chronotype.

**Potential mediators**

*Circadian rest-activity rhythms*

Within the entire cohort, a total of 87 577 participants were screened for the mediation analysis of CRARs. Physical activity data were segmented into 5-second episodes (UK Biobank field ID 90004). We derived seven key non-parametric parameters (by the R package 'nparACT') and three cosine-fitted parameters (by the R package 'ActCR') of circadian rest-activity rhythms (CRARs). These metrics include (1) L5, the average activity during the least active 5 hours, which reflects rest periods; (2) M10, the average activity during the most active 10 hours, which indicates the intensity of activity during waking hours; (3) Relative amplitude (RA), calculated as RA = (M10 - L5) / (M10 + L5), which quantifies the relative difference between M10 and L5 over a 24-hour period, with larger values indicating a greater contrast between the most and least active periods of the day; (4) L5 onset, which indicates the timing of the least active period and is usually associated with sleep onset; (5) M10 onset, which indicates the timing of the most active period and reflects peak activity; (6) Interdaily stability (IS), which measures the consistency of circadian rhythms across days, with higher values indicating stronger circadian rhythms; (7) Intradaily variability (IV), which assesses the degree of fragmentation of resting-activity rhythms, with higher values indicating greater fragmentation of circadian rhythms; (8) Amplitude, the amplitude of resting-activity rhythms; (9) Acrophase, the time to peak of resting-activity rhythms; and (10) MESOR, the median estimation statistic of rhythms. These measures have been thoroughly defined and reviewed in the previous literature^4,5^.

*Neuroimaging acquisition and processing*

The mediation analysis of brain structures was carried out with a total of 16 855 participants. In this study, brain MRI was conducted between 2014 and 2020, with a median interval of 3.38 years following the baseline assessment of the present study. Quality-controlled T1-weighted neuroimaging data processed with FreeSurfer were utilised^6^. Imaging protocols were designed by the UK Biobank Imaging Working Group. The dataset comprises 66 cortical regions (33 in each hemisphere), parcellated according to the Desikan-Killiany atlas (category ID = 192), and 33 subcortical regions, identified through subcortical volumetric segmentation (ASEG, category ID = 190).

*Serum vitamin D concentration*

We screened a total of 78 950 participants for the mediating analysis of serum vitamin D concentration. Vitamin D levels were indicated by serum 25(OH)D concentrations (field ID 30890). We included participants with both light data and serum 25(OH)D data in the mediation analysis. The concentration of 25(OH)D (10–240 nmol/L) did not fall outside the valid range of the assay (10–375 nmol/L)^7^.

**References**

1. Burns AC, Windred DP, Rutter MK, et al. Day and night light exposure are associated with psychiatric disorders: an objective light study in >85,000 people. *Nature Mental Health* 2023.

2. Bycroft C, Freeman C, Petkova D, et al. The UK Biobank resource with deep phenotyping and genomic data. *Nature* 2018; **562**(7726): 203–9.

3. Windred DP, Burns AC, Rutter MK, et al. Personal light exposure patterns and incidence of type 2 diabetes: analysis of 13 million hours of light sensor data and 670,000 person-years of prospective observation. *The Lancet Regional Health–Europe* 2024.

4. Lyall LM, Wyse CA, Graham N, et al. Association of disrupted circadian rhythmicity with mood disorders, subjective wellbeing, and cognitive function: a cross-sectional study of 91 105 participants from the UK Biobank. *Lancet Psychiatry* 2018; **5**(6): 507–14.

5. Blume C, Santhi N, Schabus M. 'nparACT' package for R: A free software tool for the non-parametric analysis of actigraphy data. *MethodsX* 2016; **3**: 430–5.

6. Miller KL, Alfaro-Almagro F, Bangerter NK, et al. Multimodal population brain imaging in the UK Biobank prospective epidemiological study. *Nat Neurosci* 2016; **19**(11): 1523–36.

7. Revez JA, Lin T, Qiao Z, et al. Genome-wide association study identifies 143 loci associated with 25 hydroxyvitamin D concentration. *Nat Commun* 2020; **11**(1): 1647.

# **Supplemental figure 1 The 24-hour light-exposure profile**


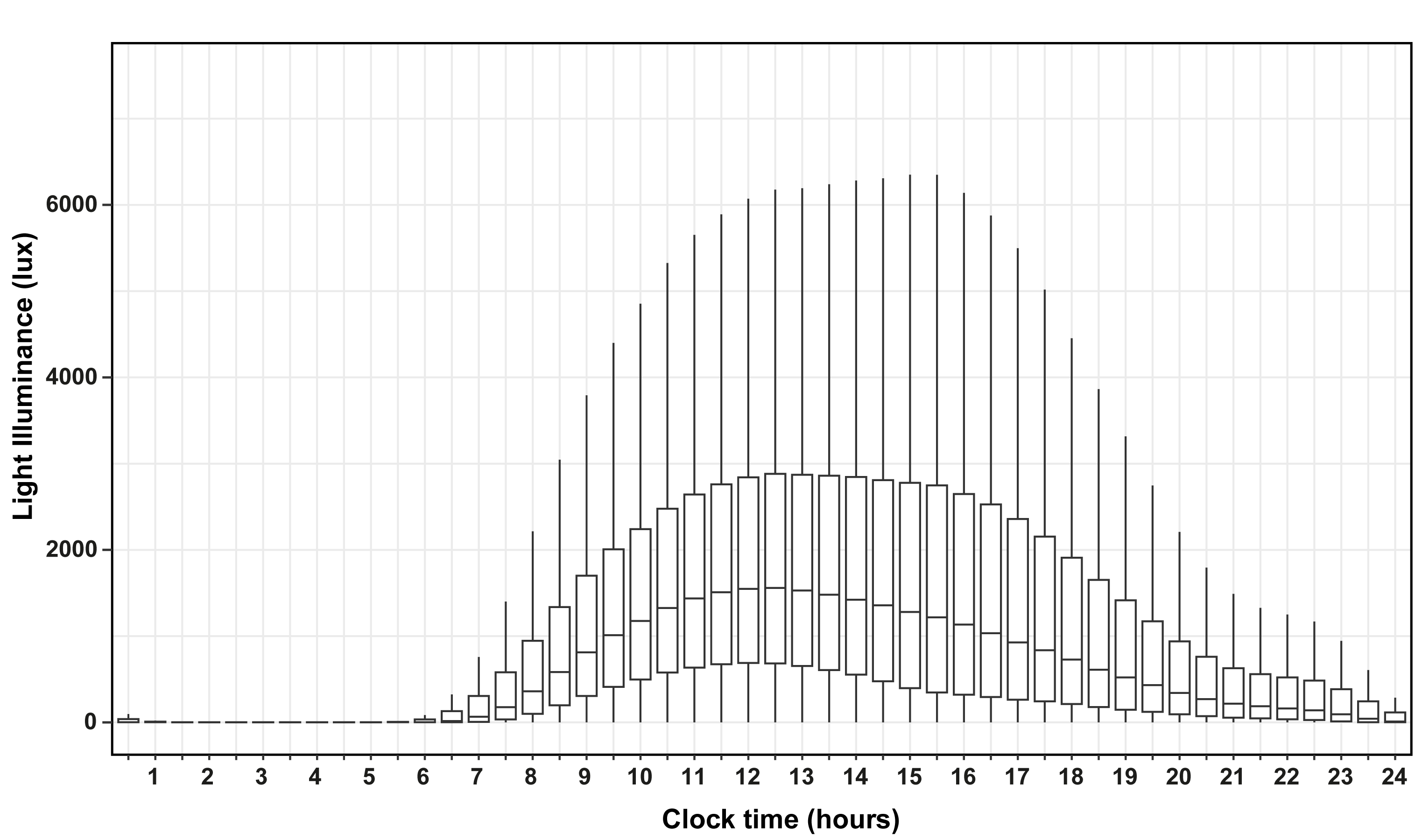
24-hour light-exposure profile in the total sample (n = 87 577). Boxplots correspond to the median and IQR range of light exposure in a given 30-minute bin. Whiskers correspond to 1*IQR.

# **Supplemental figure 2 Association between daytime and nighttime light exposure (continuous variables) and dementia risk**

The dashed line represents the minimally adjusted hazard ratios (adjusted for age and sex), and the solid line stands for the fully adjusted hazard ratios. Shaded areas represent the 95% confidence bands in fully adjusted models.

# **Supplemental figure 3 Timeline of some covariates collection**

The covariates with repeated measurements including education level, healthy diet score, vitamin D supplement use, smoking status, alcohol consumption, chronotype, obesity, diabetes history, and hypertension history were obtained from touchscreen questionnaires at the time-point closest to the accelerometery.

# **Supplemental figure 4 The missing data pattern of the covariates**

###
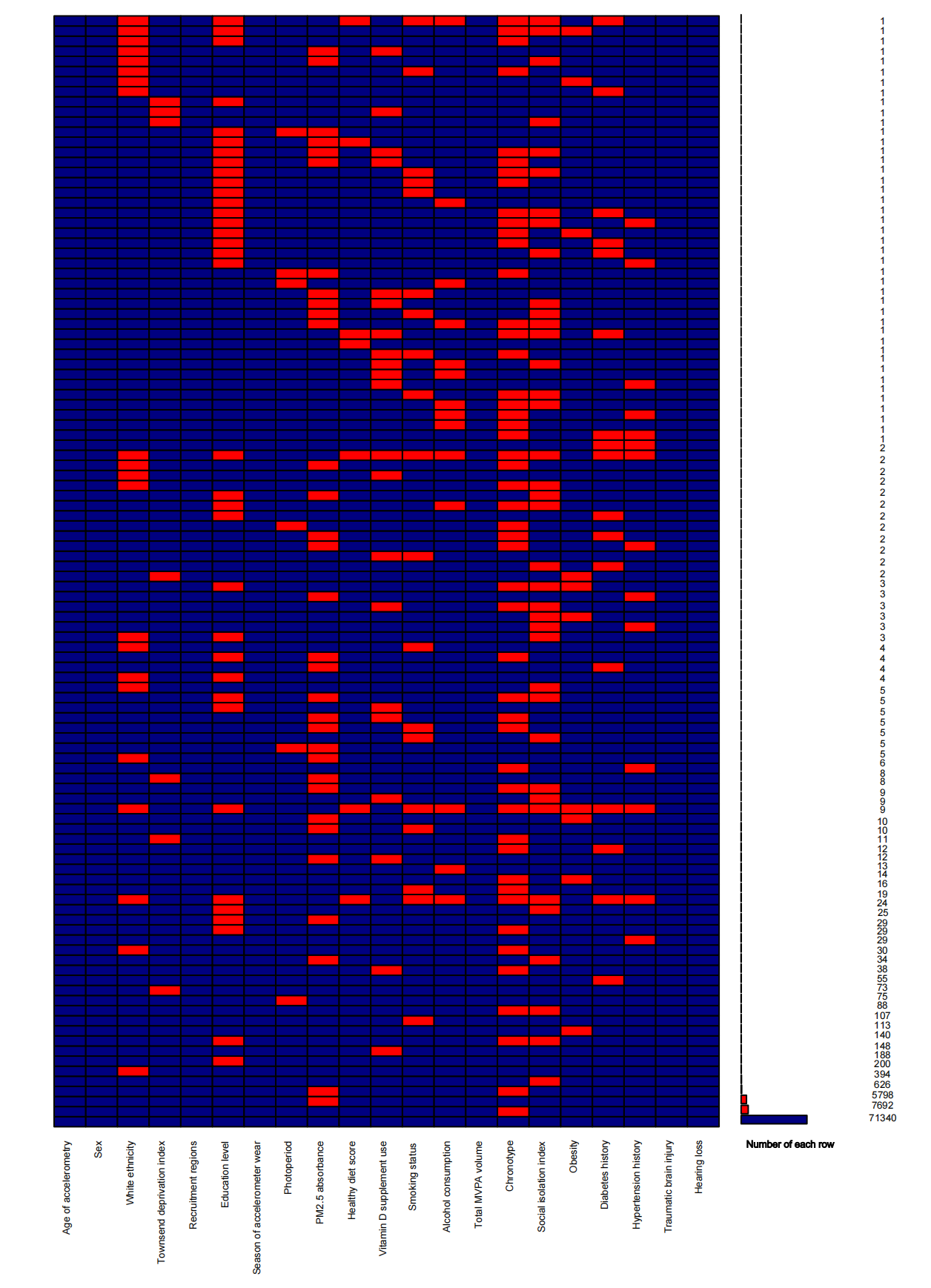


Navy blue cell: observed (or non-missing) data; Red cell: missing data. Among 87 577 participants, 71 340 (81.46%) had no missing data. Overall, the missing data of the covariates show a general pattern where missing values are dispersed all over the dataset.

# **Supplemental figure 5 Scaled Schoenfeld residual plots for dementia risk**

###
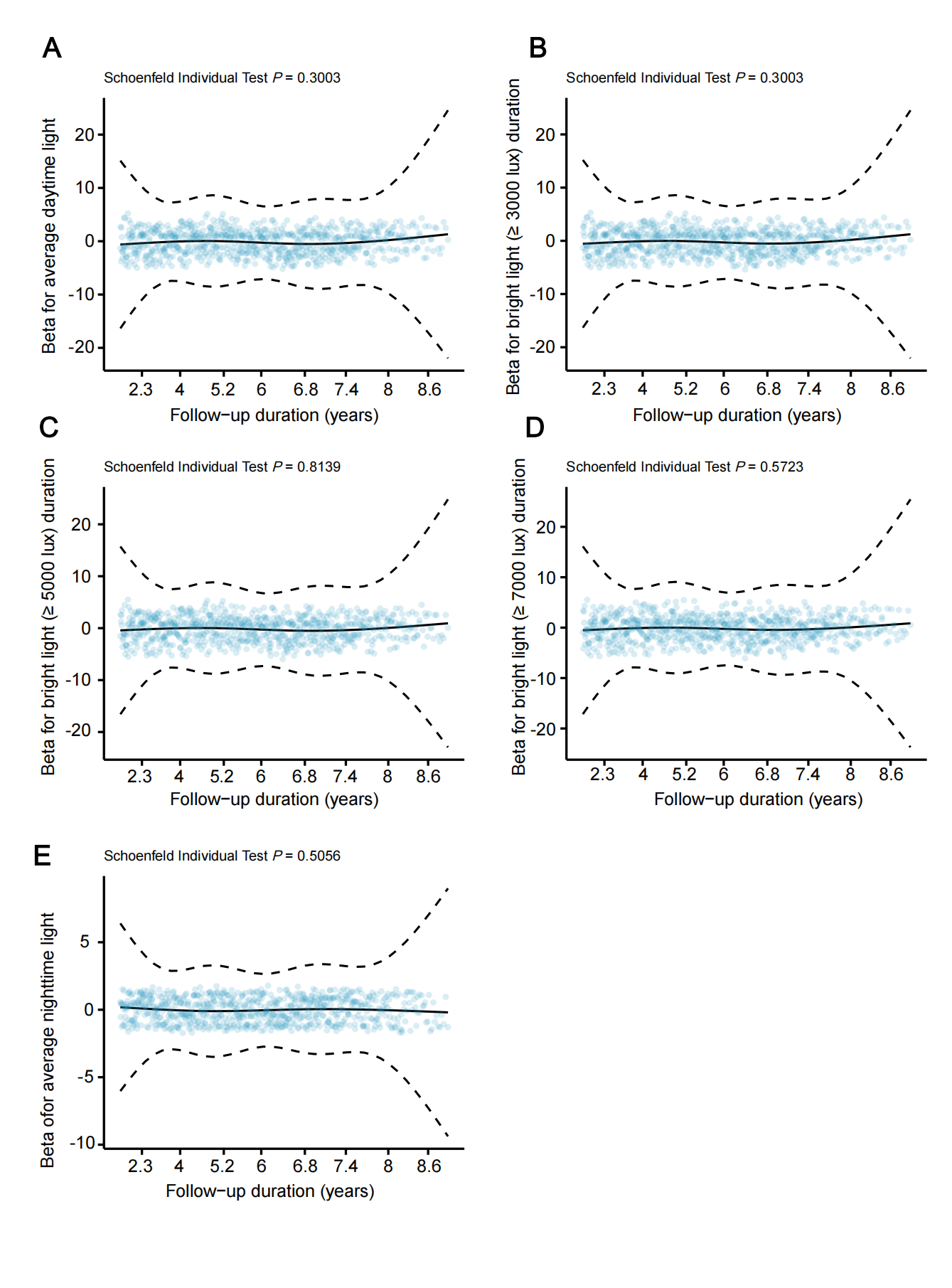


As a rule of thumb, a non-zero slope in solid line indicate a violation of the proportional hazard assumption. The dotted lines outline the 95% confidence interval.

# **Supplemental figure 6 The cumulative risk curves for dementia based on the categories of daytime light exposures**

**
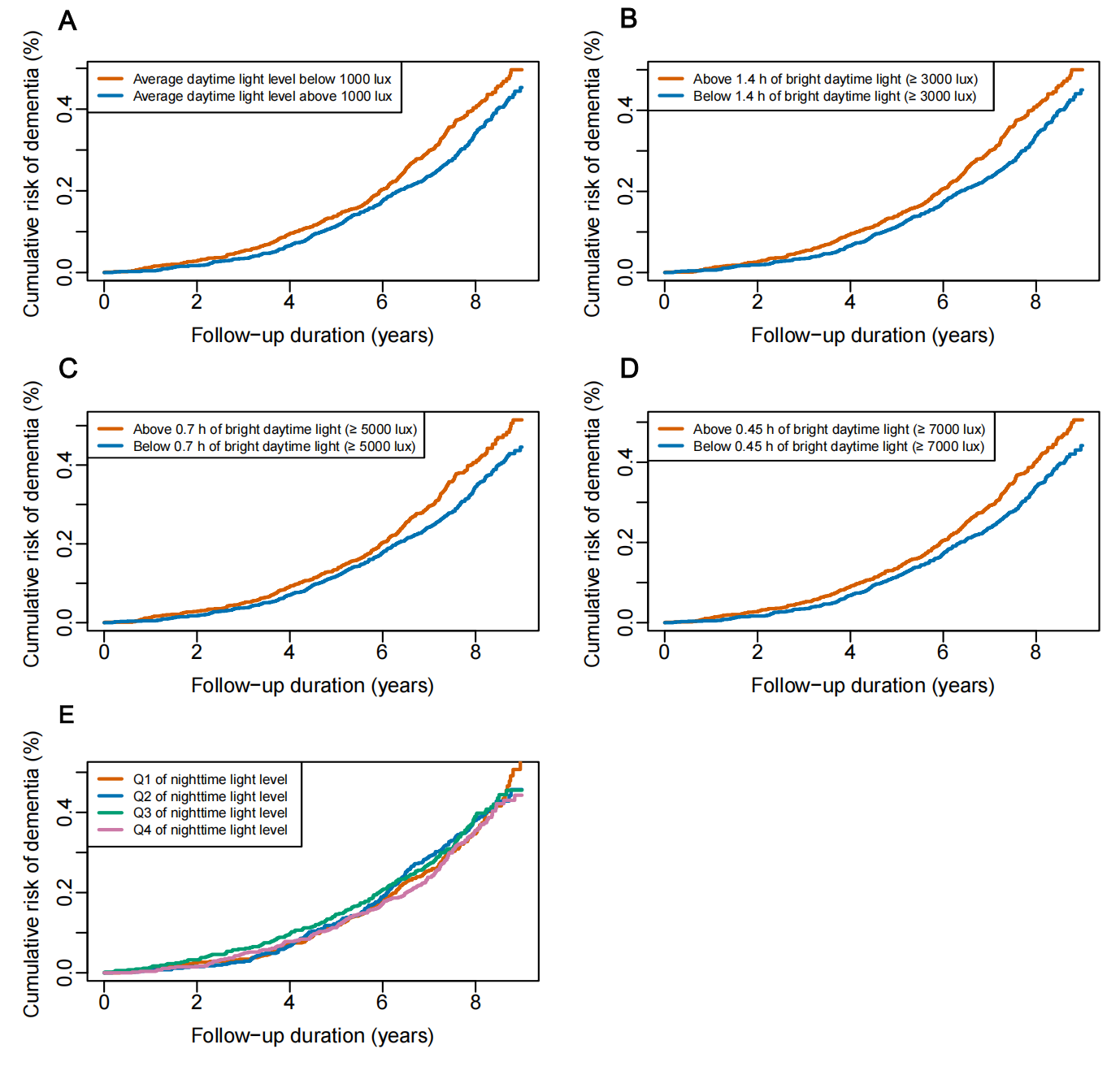
A** Cumulative risk of dementia stratified by average daytime light. **B** Cumulative risk of dementia stratified by the duration of bright light above 3000 lux. **C** Cumulative risk of dementia stratified by the duration of bright light above 5000 lux. **D** Cumulative risk of dementia stratified by the duration of bright light above 7000 lux. **E** Cumulative risk of dementia stratified by average nighttime light level. Analyses were adjusted for age, sex, ethnicity, Townsend deprivation index, recruitment centre, education level, season of accelerometer wear, photoperiod, PM2.5 absorbance, healthy diet score, vitamin D supplement use, smoking status, alcohol intake, total MVPA volumes, chronotype, social isolation, obesity, diabetes history, hypertension history, traumatic brain injury, and hearing loss (Model 4). Q, quartile.

# **Supplemental figure 7 Associations between daytime light exposures and cortical structures**


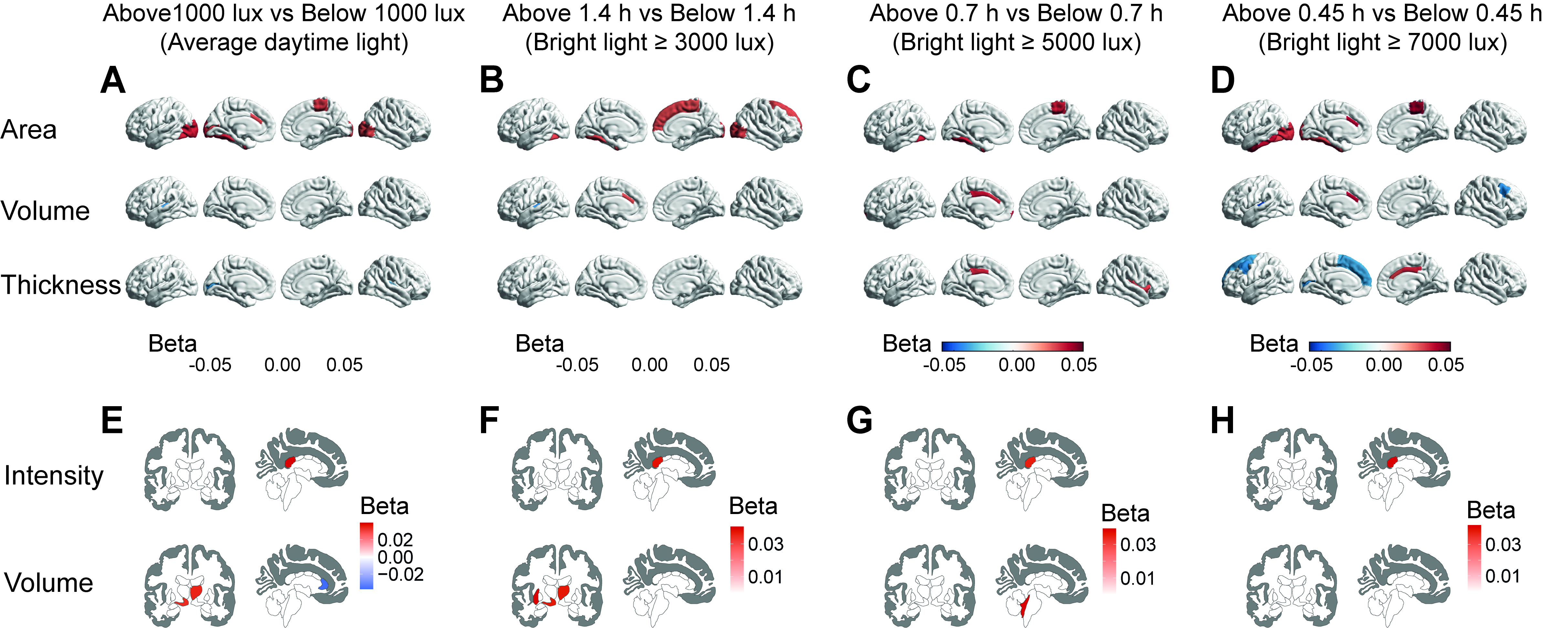


**A–D** Associations between four daytime light exposures and cortical structures, including cortical area, volume and thickness. **E–H** Associations between four daytime light exposures and subcortical structures, including intensity and volume. Analyses were adjusted for age, sex, ethnicity, Townsend deprivation index, recruitment centre, education level, season of accelerometer wear, photoperiod, PM2.5 absorbance, healthy diet score, vitamin D supplement use, smoking status, alcohol intake, MVPA, chronotype, social isolation index, obesity, history of diabetes, hypertension, traumatic brain injury and hearing loss. The colour bar indicates β values; red indicates positive associations and blue indicates negative associations.

# **Supplemental table 1.** **The information sources and codes of outcomes**

| **Outcome** | **Code sources** | **Code type** | **Codes** |
| --- | --- | --- | --- |
| **All-cause dementia** | **Hospital records** | ICD-10 | A81.0, F00, F00.0, F00.1, F00.2, F00.9, F01, F01.0, F01.1, F01.2, F01.3, F01.8, F01.9, F02, F02.0, F02.1, F02.2, F02.3, F02.4, F02.8, F03, F05.1, F10.6, G30, G30.0, G30.1, G30.8, G30.9, G31.0, G31.1, G31.8, I67.3 |
|  | **Death**  **Registry** |  |  |
|  | **Primary care data** | Read V2 | 1461, A411., A4110, E00.., E000., E001., E0010,E0011, E0012, E0013, E001z, E002., E0020, E0021,E002z, E003., E004., E0040, E0041, E0042, E0043,E004z, E012., E02y1, E041., Eu00., Eu000, Eu001,Eu002, Eu00z, Eu01., Eu010, Eu011, Eu012, Eu013,Eu01y, Eu01z, Eu02., Eu020, Eu021, Eu022, Eu023,Eu024, Eu025, Eu02y, Eu02z, Eu041, Eu106, Eu107, F110., F1100, F1101, F111., F112., F116., F118., F11x2, F11x7, F11x9, F11y2, F21y2, Fyu30, 38C13, 3AE3., 3AE4., 3AE5., 3AE6., 66h.., 6AB.., 8BM02, 8BM50, 8BM60, 8BPa., 8CMe0, 8CMG2, 8CMZ., 8CMZ0, 8CMZ1, 8CMZ2, 8CMZ3, 8CSA., 8Hla., 8IAe0, 8IAe2, 9hD.., 9hD0., 9hD1., 9Ou.., 9Ou1., 9Ou2., 9Ou3., 9Ou4., 9Ou5. |
|  |  | Read CTV3 | .1461,1461, .E11., .E111, .E112, .E113, .E114, .E115, .E116, .E11Z, .F21Z, .F371, .G78., A411., A4110,E00.., E000., E001., E0010, E0011, E0012, E0013,E001z, E002., E0020, E0021, E002z, E003., E004.,E0040, E0041, E0042, E0043, E004z, E012., E02y1,E041., Eu00., Eu000, Eu001, Eu002, Eu00z, Eu01.,Eu010, Eu011, Eu012, Eu013, Eu01y, Eu01z, Eu02.,Eu020, Eu021, Eu022, Eu023, Eu024, Eu025,Eu02y, Eu02z, Eu041, F110., F1100, F1101, F111., F112., F116., F118., F11x2, F11x7, F11y2, F21y2, Fyu30, Ub1T6, X002m, X002w, X002x, X002y, X002z, X0030, X0031, X0032, X0033, X0034, X0035, X0036, X0037, X0039, X003A, X003B, X003C, X003D, X003E, X003F, X003G, X003H, X003I, X003J, X003l, X003m, X003P, X003R, X003T, X003V, X003W, X003X, X00R2, X00Rk, Xa0lH, Xa0sC, Xa0sE, Xa1GB, Xa25J, Xa3ez, XaA1S, XabVp, XaE74, XaIKB, XaIKC, XaKyY, XaOfZ, XE17j, XE1aG, XE1Xs, XE1Xu, XE1Z6, .3AE3, .3AE4, .3AE5, .3AE6, .66h., .6AB., .9hD1, .9Ou., .9Ou1, .9Ou2, .9Ou3, .9Ou4, .9Ou5, 3AE3., 3AE4., 3AE5., 3AE6., 66h.., 6AB.., 8BM02, 8BM50, 8BPa., 8CMe0, 8CMG2, 8CMZ., 8CMZ0, 8CMZ1, 8CMZ2, 8CMZ3, 8CSA., 8IAe0, 8IAe2, 9hD1., 9Ou., 9Ou1., 9Ou2., 9Ou3., 9Ou4., 9Ou5., Xa0fZ, XaaBZ, XaaeA, XaaiW, Xabd2, Xabd3, XabEk, XabEl, XabtQ, XacIx, XacIy, XacIz, XacJ0, XacLx, Xacly, Xaclz, XacM2, Xaefu, XaJBQ, XaJBU, XaJBV, XaJBW, XaJBX, XaJPy, XaLFf, XaLFo, XaLFp, XaMFy, XaMG0, XaMGF, XaMGG, XaMGI, XaMGJ, XaMGK, XaMJC, XaYFR, XaYPX, XaZqJ, XaZWz |

# **Supplemental table 2.** **The information sources and codes of covariates**

| **Variables** | **Touchscreen questionnaire** | | **Hospital records** | **Accelerometer** |
| --- | --- | --- | --- | --- |
|  | **Initial assessment** | **Repeated assessments** |  |  |
| Age at accelerometery | Field ID 34&52 | - | - | Field ID 90010 |
| Sex | Field ID 31 | - | - | - |
| Ethnicity | Field ID 21000 | - | - | - |
| Townsend deprivation index | Field ID 22189 | - | - | - |
| Recruitment regions | Field ID 54 | - | - | - |
| Education level | Field ID 6138 | Field ID 6138 |  |  |
| Season of accelerometer wear | - | - | - | Field ID 90010 |
| Photoperiod | - | - | - | - |
| PM2.5 absorbance | Field ID 24007 | - | - | - |
| Healthy diet score | Field ID 1289/1299/1309/1319/1329/1339/1349/1369/1379/1389 | Field ID 1289/1299/1309/1319/1329/1339/1349/1369/1379/1389 | - | - |
| Vitamin D supplement use | Field ID 20084 | - | - | - |
| Smoking status | Field ID 20116 | Field ID 20116 | - | - |
| Alcohol consumption | Field ID 1558 | Field ID 1558 | - | - |
| MVPA volume | - | - | - | Field ID 90004 |
| Chronotype | Field ID 1180 | - | - | - |
| Health status |  |  |  |  |
| Obesity | Field ID 21001 | Field ID 21001 | - | - |
| Diabetes history | Field ID 2443 | Field ID 2443 | ICD E10-E14 |  |
| Hypertension history | Field ID 6150 | Field ID 6150 | ICD I10 | - |
| Traumatic brain injury | - | - | ICD S060-S0691 | - |
| Hearing loss | - | - | ICD H900-C919 | - |

# **Supplemental table 3. The numbers (percentages) of participants with missing covariates**

| **Covariates** | **n** | **%** |
| --- | --- | --- |
| Any missing covariate | 16 237 | 18.54 |
| Age at accelerometery | 0 | 0 |
| Sex | 0 | 0 |
| White ethnicity | 306 | 0.35 |
| Townsend deprivation index | 100 | 0.11 |
| Recruitment regions | 0 | 0 |
| Education level | 497 | 0.57 |
| Season of accelerometer wear | 0 | 0 |
| Photoperiod | 86 | 0.10 |
| PM2.5 absorbance | 6598 | 7.53 |
| Healthy diet score | 40 | 0.05 |
| Vitamin D supplement use | 238 | 0.27 |
| Smoking status | 198 | 0.23 |
| Alcohol consumption | 61 | 0.07 |
| Total MVPA volume | 0 | 0 |
| Chronotype | 8813 | 10.06 |
| Social isolation index | 790 | 0.90 |
| Obesity | 161 | 0.18 |
| Diabetes history | 121 | 0.14 |
| Hypertension history | 87 | 0.10 |
| Traumatic brain injury | 0 | 0 |
| Hearing loss | 0 | 0 |

# **Supplemental table 4. Baseline characteristics of overall sample and complete case sample**

| **Characteristics** | **Overall sample**  **[dataset with imputation]**  **(n = 87 577)** | **Complete case sample**  **[excluding those with any missing covariates from overall sample]**  **(n = 71 340)** |
| --- | --- | --- |
| **Age at accelerometery (years), mean (SD)** | 62.36(7.83) | 62.28 (7.83) |
| **Sex (female/male), n (%)** | 49 898/37 679 (56.98) | 41 470/29 783 (58.13) |
| **White ethnicity, n (%)** | 84 937 (96.99) | 69 158 (96.94) |
| **Townsend deprivation index, median [IQR]** | −2.47 [3.59] | −2.49 [3.54] |
| **Recruitment regions, n (%)** |  |  |
| England | 78 549 (89.69) | 68 391 (95.87) |
| Wales | 3308 (3.78) | 2905 (4.07) |
| Scotland | 5720 (6.53) | 44 (0.06) |
| **Education level, n (%)** |  |  |
| College degree or above | 38 222 (43.64) | 30 582 (42.87) |
| Other professional qualification | 42 162 (48.14) | 34 950 (48.99) |
| No formal qualification | 7193 (8.21) | 5808 (8.14) |
| **Season of accelerometer wear, n (%)** |  |  |
| Spring | 20 247 (23.12) | 16 440 (23.04) |
| Summer | 23 925 (27.32) | 19 441(27.25) |
| Autumn | 25 763 (29.42) | 21 064 (29.53) |
| Winter | 17 642 (20.14) | 14 395 (20.18) |
| **Photoperiod, mean (SD)** | 12.48 (3.16) | 12.48(3.16) |
| **PM2.5 absorbance, mean (SD)** | 1.17 (0.27) | 1.17 (0.27) |
| **Healthy diet score, mean (SD)** | 2.69 (1.17) | 2.71 (1.16) |
| **Vitamin D supplement use, n (%)** | 5917 (6.76) | 4905 (6.88) |
| **Smoking status, n (%)** |  |  |
| Never | 50 305 (57.44) | 40 672 (57.01) |
| Previous | 31 750 (36.25) | 26 154 (36.66) |
| Current | 5522 (6.31) | 4514 (6.33) |
| **Alcohol consumption, n (%)** |  |  |
| Not current | 5204 (5.94) | 4233 (5.93) |
| Two or less times a week | 40 440 (46.18) | 32 965 (46.21) |
| Three or more times a week | 41 933 (47.88) | 34 142(47.86) |
| **Total MVPA volume (min/week), median [IQR]** | 114.67 [159.50] | 113.67 [158.00] |
| **Chronotype, n (%)** |  |  |
| Morning | 21 565 (24.62) | 18 911 (26.51) |
| Morning preference | 35 912 (41.01) | 26 823 (37.60) |
| Evening preference | 22 955 (26.21) | 19 220 (26.94) |
| Evening | 7145 (8.16) | 6386 (8.95) |
| **Social isolation index, n (%)** |  |  |
| least | 41 012 (46.83) | 33 450 (46.89) |
| moderate | 35 055 (40.03) | 28 521 (39.98) |
| most | 11 510 (13.14) | 9369 (13.13) |
| **Health status, n (%)** |  |  |
| Obesity | 17 086 (19.51) | 14 035 (19.67) |
| Diabetes history | 4167 (4.76) | 3409 (4.78) |
| Hypertension history | 23 766 (27.14) | 19 552 (27.41) |
| Traumatic brain injury | 154 (0.18) | 119 (0.17) |
| Hearing loss | 598 (0.68) | 492 (0.69) |

# **Supplemental table 5. Pearson correlation coefficient matrix of variables included in the fully adjusted model**

|  | **Age** | **Sex** | **Ethn icity** | **TDI** | **Recr uitm ent**  **cente r** | **Educ ation** | **Wear seaso n** | **PM 2.5** | **Phot operi od** | **Smo king** | **Alcoh ol** | **Heal thy**  **diet score** | **Vita min D** | **MV PA** | **Chro notyp e** | **Soci al**  **isol atio n** | **Obes ity** | **Dia bete s** | **Hyp erte nsio n** | **TBI** | **HL** |
| --- | --- | --- | --- | --- | --- | --- | --- | --- | --- | --- | --- | --- | --- | --- | --- | --- | --- | --- | --- | --- | --- |
| **Age** | 1.00 |  |  |  |  |  |  |  |  |  |  |  |  |  |  |  |  |  |  |  |  |
| **Sex** | 0.07 | 1.00 |  |  |  |  |  |  |  |  |  |  |  |  |  |  |  |  |  |  |  |
| **Ethnicity** | 0.11 | 0.01 | 1.00 |  |  |  |  |  |  |  |  |  |  |  |  |  |  |  |  |  |  |
| **TDI** | −0.11 | −0.02 | −0.13 | 1.00 |  |  |  |  |  |  |  |  |  |  |  |  |  |  |  |  |  |
| **Recruitment center** | 0.01 | <0.01 | 0.04 | −0.03 | 1.00 |  |  |  |  |  |  |  |  |  |  |  |  |  |  |  |  |
| **Education** | 0.17 | −0.01 | 0.04 | 0.01 | −0.03 | 1.00 |  |  |  |  |  |  |  |  |  |  |  |  |  |  |  |
| **Wear season** | 0.02 | <0.01 | <0.01 | <0.01 | −0.02 | <0.01 | 1.00 |  |  |  |  |  |  |  |  |  |  |  |  |  |  |
| **PM 2.5** | −0.08 | −0.02 | −0.13 | 0.41 | −0.07 | −0.05 | −0.01 | 1.00 |  |  |  |  |  |  |  |  |  |  |  |  |  |
| **Photoperiod** | −0.01 | <0.01 | <0.01 | 0.02 | 0.01 | <0.01 | −0.19 | 0.01 | 1.00 |  |  |  |  |  |  |  |  |  |  |  |  |
| **Smoking** | 0.05 | 0.09 | 0.03 | 0.12 | −0.01 | 0.10 | 0.01 | 0.06 | 0.01 | 1.00 |  |  |  |  |  |  |  |  |  |  |  |
| **Alcohol** | 0.07 | 0.14 | 0.12 | −0.07 | <0.01 | −0.11 | <0.01 | −0.02 | −0.01 | 0.12 | 1.00 |  |  |  |  |  |  |  |  |  |  |
| **Healthy diet score** | 0.10 | −0.19 | −0.02 | <0.01 | −0.03 | −0.05 | <0.01 | 0.02 | <0.01 | −0.03 | −0.04 | 1.00 |  |  |  |  |  |  |  |  |  |
| **Vitamin D** | 0.05 | −0.08 | −0.01 | 0.01 | −0.01 | −0.03 | <0.01 | 0.06 | <0.01 | −0.02 | −0.02 | 0.07 | 1.00 |  |  |  |  |  |  |  |  |
| **MVPA** | −0.21 | 0.10 | <0.01 | 0.01 | 0.01 | −0.11 | −0.03 | 0.02 | 0.05 | −0.06 | 0.06 | 0.05 | −0.01 | 1.00 |  |  |  |  |  |  |  |
| **Chronotype** | −0.08 | 0.01 | −0.01 | 0.04 | 0.01 | −0.05 | <0.01 | 0.05 | <0.01 | 0.09 | 0.04 | −0.07 | <0.01 | −0.09 | 1.00 |  |  |  |  |  |  |
| **Social**  **isolation** | −0.06 | 0.03 | −0.04 | 0.14 | −0.02 | <0.01 | <0.01 | 0.07 | 0.01 | 0.05 | −0.08 | −0.03 | <0.01 | <0.01 | 0.02 | 1.00 |  |  |  |  |  |
| **Obesity** | <0.01 | 0.04 | −0.01 | 0.06 | <0.01 | 0.09 | <0.01 | 0.01 | <0.01 | 0.05 | −0.09 | −0.06 | −0.03 | −0.19 | 0.03 | 0.05 | 1.00 |  |  |  |  |
| **Diabetes** | 0.08 | 0.08 | −0.04 | 0.04 | −0.01 | 0.05 | 0.01 | 0.01 | <0.01 | 0.05 | −0.06 | −0.02 | <0.01 | −0.09 | 0.01 | 0.03 | 0.17 | 1.00 |  |  |  |
| **Hypertension** | 0.23 | 0.11 | −0.01 | 0.02 | −0.02 | 0.11 | 0.01 | <0.01 | <0.01 | 0.05 | −0.01 | −0.01 | −0.01 | −0.14 | −0.01 | 0.02 | 0.20 | 0.19 | 1.00 |  |  |
| **TBI** | <0.01 | 0.02 | <0.01 | 0.01 | <0.01 | −0.01 | 0.01 | 0.01 | <0.01 | <0.01 | <0.01 | <0.01 | <0.01 | 0.01 | <0.01 | 0.01 | −0.01 | <0.01 | 0.01 | 1.00 |  |
| **HL** | 0.04 | 0.01 | <0.01 | <0.01 | −0.02 | 0.02 | 0.01 | <0.01 | <0.01 | 0.01 | −0.01 | <0.01 | 0.01 | −0.02 | <0.01 | <0.01 | 0.01 | 0.02 | 0.03 | 0.01 | 1.00 |

Rule of thumb for interpreting the size of a correlation coefficient: 0·00 to 0·30 (0·00 to −0·30), negligible correlation; 0·30 to 0·50 (−0·30 to −0·50), low positive (negative) correlation; 0·50 to 0·70 (−0·50 to −0·70), moderate positive (negative) correlation; 0·70 to 0·90 (−0·70 to −0·90), high positive (negative) correlation; 0·9 to 1·0 (−0·9 to −1·0), very high positive (negative) correlation. **TDI**: Townsend deprivation index; **MVPA**: moderate to vigorous physical activity; **TBI:** traumatic brain injury; **HL**: Hearing loss

# **Supplemental table 6. Baseline characteristics of the study participants by incident dementia status**

| **Characteristics** | **Overall**  **(n = 87 577)** | **No incident dementia (n = 86 836)** | **Incident dementia**  **(n = 741)** |
| --- | --- | --- | --- |
| **Age at accelerometery (years), mean (SD)** | 62.36 (7.83) | 62.29 (7.82) | 70.09 (4.93) |
| **Sex (female/male), n (%)** | 49 898/37 679 (56.98) | 49 552/37 284 (57.06) | 346/395 (46.69) |
| **White ethnicity, n (%)** | 84 937 (96.99) | 84 215 (96.98) | 722 (97.44) |
| **Townsend deprivation index, median [IQR]** | −2.47 [3.59] | −2.47 [3.59] | −2.61 [3.78] |
| **Recruitment regions, n (%)** |  |  |  |
| England | 78 549 (89.69) | 77 866 (89.67) | 683 (92.17) |
| Wales | 3308 (3.78) | 3288 (3.79) | 20 (2.70) |
| Scotland | 5720 (6.53) | 5682 (6.54) | 38 (5.13) |
| **Education level, n (%)** |  |  |  |
| College degree or above | 38 222 (43.64) | 37 944 (43.70) | 278 (37.52) |
| Other professional qualification | 42 162 (48.14) | 41 828 (48.17) | 334 (45.07) |
| No formal qualification | 7193 (8.21) | 7064 (8.13) | 129 (17.41) |
| **Season of accelerometer wear, n (%)** |  |  |  |
| Spring | 20 247 (23.12) | 20 071 (23.11) | 176 (23.75) |
| Summer | 23 925 (27.32) | 23 709 (27.30) | 216 (29.15) |
| Autumn | 25 763 (29.42) | 25 551 (29.42) | 212 (28.61) |
| Winter | 17 642 (20.14) | 17 505 (20.16) | 137 (18.49) |
| **Photoperiod, mean (SD)** | 12.48 (3.16) | 12.48 (3.16) | 12.58 (3.19) |
| **PM2.5 absorbance, mean (SD)** | 1.17 (0.27) | 1.17 (0.27) | 1.17 (0.28) |
| **Healthy diet score, mean (SD)** | 2.69 (1.17) | 2.69 (1.17) | 2.80 (1.23) |
| **Vitamin D supplement use, n (%)** | 5917 (6.76) | 5871 (6.76) | 46 (6.21) |
| **Smoking status, n (%)** |  |  |  |
| Never | 50 305 (57.44) | 49 958 (57.53) | 347 (46.83) |
| Previous | 31 750 (36.25) | 31 395 (36.15) | 355 (47.91) |
| Current | 5522 (6.31) | 5483 (6.31) | 39 (5.26) |
| **Alcohol consumption, n (%)** |  |  |  |
| Not current | 5204 (5.94) | 5123 (5.90) | 81 (10.93) |
| Two or less times a week | 40 440 (46.18) | 40 139 (46.22) | 301 (40.62) |
| Three or more times a week | 41 933 (47.88) | 41 574 (47.88) | 359 (48.45) |
| **Total MVPA volume (min/week), median [IQR]** | 114.67 [159.50] | 115.33 [159.67] | 60.58 [113.97] |
| **Chronotype, n (%)** |  |  |  |
| Morning | 21 565 (24.62) | 21 355 (24.59) | 210 (28.34) |
| Morning preference | 35 912 (41.01) | 35 611 (41.01) | 301 (40.62) |
| Evening preference | 22 955 (26.21) | 22 774 (26.23) | 181 (24.43) |
| Evening | 7145 (8.16) | 7096 (8.17) | 49 (6.61) |
| **Social isolation index, n (%)** |  |  |  |
| least | 41 012 (46.83) | 40 680 (46.85) | 332 (44.80) |
| moderate | 35 055 (40.03) | 34 746 (40.01) | 309 (41.70) |
| most | 11 510 (13.14) | 11 410 (13.14) | 100 (13.50) |
| **Health status, n (%)** |  |  |  |
| Obesity | 17 086 (19.51) | 16 929 (19.50) | 158 (21.32) |
| Diabetes history | 4167 (4.76) | 4078 (4.70) | 89 (12.01) |
| Hypertension history | 23 766 (27.14) | 23 429 (26.98) | 337 (45.48) |
| Traumatic brain injury | 154 (0.18) | 153 (0.18) | 1 (0.13) |
| Hearing loss | 598 (0.68) | 585 (0.67) | 13 (1.75) |

Data are mean (standard deviation) or n (%) unless noted otherwise. IQR: interquartile range; MVPA: moderate to vigorous physical activity.

# **Supplemental table 7. Baseline characteristics of the study participants by average daytime light**

| **Characteristics** | **Overall**  **(n = 87 577)** | **Average daytime light**  **below 1000 lux (n = 40 553)** | **Average daytime light**  **above 1000 lux**  **(n = 47 024)** |
| --- | --- | --- | --- |
| **Age at accelerometry (years), mean (SD)** | 62.36 (7.83) | 62.00 (7.95) | 62.67 (7.72) |
| **Sex (female/male), n (%)** | 49 898/37 679 (56.98) | 23 330/17 223 (57.52) | 26 568/20 456 (56.50) |
| **White ethnicity, n (%)** | 84 937 (96.99) | 39 039 (96.27) | 45 898 (97.61) |
| **Townsend deprivation index, median [IQR] Recruitment regions** | −2.47 [3.59] | −2.32 [3.84] | −2.59 [3.37] |
| England | 78 549 (89.69) | 36 228 (89.33) | 42 321 (90.00) |
| Wales | 3308 (3.78) | 1425 (3.51) | 1883 (4.00) |
| Scotland | 5720 (6.53) | 2900 (7.15) | 2820 (6.00) |
| **Education level, n (%)** |  |  |  |
| College degree or above | 38 222 (43.64) | 18 132 (44.71) | 20 090 (42.72) |
| Other professional qualification | 42 162 (48.14) | 19 156 (47.24) | 23 006 (48.92) |
| No formal qualification | 7193 (8.21) | 3265 (8.05) | 3928 (8.35) |
| **Season of accelerometer wear, n (%)** |  |  |  |
| Spring | 20 247 (23.12) | 7266 (17.92) | 12 981 (27.61) |
| Summer | 23 925 (27.32) | 4265 (10.52) | 19 660 (41.81) |
| Autumn | 25 763 (29.42) | 15 238 (37.58) | 10 525 (22.38) |
| Winter | 17 642 (20.14) | 13 784 (33.99) | 3858 (8.20) |
| **Photoperiod, mean (SD)** | 12.48 (3.16) | 10.81 (2.82) | 13.93 (2.70) |
| **PM2.5 absorbance, mean (SD)** | 1.17 (0.27) | 1.18 (0.28) | 1.16 (0.26) |
| **Healthy diet score, mean (SD)** | 2.69 (1.17) | 2.66 (1.17) | 2.72 (1.17) |
| **Vitamin D supplement use Smoking status** | 5917 (6.76) | 2845 (7.02) | 3072 (6.53) |
| Never | 50 305 (57.44) | 23 571 (58.12) | 26 734 (56.85) |
| Previous | 31 750 (36.25) | 14 337 (35.35) | 17 413 (37.03) |
| Current | 5522 (6.31) | 2645 (6.52) | 2877 (6.12) |
| **Alcohol consumption, n (%)** |  |  |  |
| Not current | 5204 (5.94) | 2553 (6.30) | 2651 (5.64) |
| Two or less times a week | 40 440 (46.18) | 19 408 (47.86) | 21 032 (44.73) |
| Three or more times a week | 41 933 (47.88) | 18 592 (45.85) | 23 341 (49.64) |
| **Total MVPA volume (min/week), median [IQR] Chronotype** | 114.67 [159.50] | 104.50 [147.17] | 124.17 [170.33] |
| Morning | 21 565 (24.62) | 9533 (23.51) | 12 032 (25.59) |
| Morning preference | 35 912 (41.01) | 16 439 (40.54) | 19 473 (41.41) |
| Evening preference | 22 955 (26.21) | 10 908 (26.90) | 12 047 (25.62) |
| Evening | 7145 (8.16) | 3673 (9.06) | 3472 (7.38) |
| **Social isolation index, n (%)** |  |  |  |
| least | 41 011 (46.83) | 18 315 (45.16) | 22 697 (48.27) |
| moderate | 35 058 (40.03) | 16 462 (40.59) | 18 593 (39.54) |
| most | 11 508 (13.14) | 5776 (14.24) | 5734 (12.19) |
| **Health status, n (%)** |  |  |  |
| Obesity | 17 086 (19.51) | 8008 (19.75) | 9078 (19.31) |
| Diabetes history | 4167 (4.76) | 2026 (5.00) | 2141 (4.55) |
| Hypertension history | 23 766 (27.14) | 11 161 (27.52) | 12 605 (26.81) |
| Traumatic brain injury | 154 (0.18) | 76 (0.19) | 78 (0.17) |
| Hearing loss | 598 [0.68] | 293 [0.72] | 305 [0.65] |

The average daytime light level was dichotomised into binary categories, defined by a 1000 lux threshold for analysis. Data are mean (standard deviation) or n (%) unless noted otherwise. IQR: interquartile range; MVPA: moderate to vigorous physical activity.

# **Supplemental table 8. Sensitivity analysis on the associations between daytime light exposure** **and dementia risk by controlling for month of accelerometer wear**

| **Daytime light exposure** | **Events/n** | **Model 1**  HR (95% CI); *P* | **Model 2**  HR (95% CI); *P****^a^*** | **Model 3**  HR (95% CI); *P****^a^*** | **Model 4**  HR (95% CI); *P****^a^*** |
| --- | --- | --- | --- | --- | --- |
| **Average daytime light** | 741/87 577 |  |  |  |  |
| Below 1000 lux | 353/40 553 | 1.00 (reference) | 1.00 (reference) | 1.00 (reference) | 1.00 (reference) |
| Above 1000 lux | 388/47 024 | 0.87  (0.76–1.01);  0.062 | **0.79**  **(0.67–0.94);**  **0.007** | **0.84**  **(0.71–0.99);**  **0.037^a^** | **0.84**  **(0.71–0.99);**  **0.037^a^** |
| **Duration of bright light (≥ 3000 lux)** | 741/87 577 |  |  |  |  |
| Below 1.4 hours | 357/40 970 | 1.00 (reference) | 1.00 (reference) | 1.00 (reference) | 1.00 (reference) |
| Above 1.4 hours | 384/46 607 | **0.86**  **(0.74–0.99);**  **0.036** | **0.77**  **(0.66–0.92);**  **0.003** | **0.82**  **(0.69–0.97);**  **0.022^a^** | **0.82**  **(0.69–0.97);**  **0.022^a^** |
| **Duration of bright light (≥ 5000 lux)** | 741/87 577 |  |  |  |  |
| Below 0.7 hours | 324/37 088 | 1.00 (reference) | 1.00 (reference) | 1.00 (reference) | 1.00 (reference) |
| Above 0.7 hours | 417/50 489 | 0.87  (0.75–1.00);  0.055 | **0.78**  **(0.66–0.93);**  **0.004** | **0.83**  **(0.700–0.99);**  **0.034^a^** | **0.83**  **(0.70–0.99);**  **0.034^a^** |
| **Duration of bright light (≥ 7000 lux)** | 741/87 577 |  |  |  |  |
| Below 0.45 hours | 391/44 820 | 1.00 (reference) | 1.00 (reference) | 1.00 (reference) | 1.00 (reference) |
| Above 0.45 hours | 350/42 757 | 0.88  (0.76–1.02);  0.080 | **0.77**  **(0.64–0.92);**  **0.004** | **0.83**  **(0.69–0.99);**  **0.036^a^** | **0.83**  **(0.69–0.99);**  **0.036^a^** |

**^a^** All *P* values remained significant after multiple testing with the FDR method. Cox proportional hazard regression was used to examine the associations. **Model 1** was adjusted for age and sex. **Model 2** was adjusted as in model 1 and for ethnicity, Townsend deprivation index, recruitment centre, education level, the month of accelerometer wear, photoperiod, and PM2.5 absorbance. **Model 3** was adjusted as in model 2 and for healthy diet score, vitamin D supplement use, smoking status, alcohol intake, MVPA, chronotype, and social isolation index. **Model 4** was adjusted as in model 3 and for obesity, history of diabetes, hypertension, traumatic brain injury, and hearing loss. **HR**: hazard ratio; **MVPA**: moderate to vigorous physical activity.

# **Supplemental table 9. Sensitivity analysis on the associations between daytime light exposure and dementia risk by using the subsample with ≥6 days of accelerometer wear**

| **Daytime light exposure** | **Events/n** | **Model 1**  HR (95% CI); *P* | **Model 2**  HR (95% CI); *P* | **Model 3**  HR (95% CI); *P****^a^*** | **Model 4**  HR (95% CI); *P****^a^*** |
| --- | --- | --- | --- | --- | --- |
| **Average daytime light** | 678/79 526 |  |  |  |  |
| Below 1000 lux | 323/36 463 | 1.00 (reference) | 1.00 (reference) | 1.00 (reference) | 1.00 (reference) |
| Above 1000 lux | 355/43 063 | **0.86**  **(0.74–1.00);**  **0.049*^a^*** | **0.79**  **(0.67–0.94);**  **0.009*^a^*** | **0.83**  **(0.69–0.98);**  **0.032*^a^*** | **0.83**  **(0.70–0.99);**  **0.040^a^** |
| **Duration of bright light (≥ 3000 lux)** | 678/79 526 |  |  |  |  |
| Below 1.4 hours | 325/36 553 | 1.00 (reference) | 1.00 (reference) | 1.00 (reference) | 1.00 (reference) |
| Above 1.4 hours | 353/42 973 | **0.84**  **(0.73–0.98);**  **0.028*^a^*** | **0.77**  **(0.65–0.92);**  **0.004*^a^*** | **0.81**  **(0.68–0.97);**  **0.019*^a^*** | **0.82**  **(0.69–0.97);**  **0.024^a^** |
| **Duration of bright light (≥ 5000 lux)** | 678/79 526 |  |  |  |  |
| Below 0.7 hours | 295/32 841 | 1.00 (reference) | 1.00 (reference) | 1.00 (reference) | 1.00 (reference) |
| Above 0.7 hours | 383/46 685 | **0.84**  **(0.73–0.98);**  **0.029*^a^*** | **0.77**  **(0.64–0.92);**  **0.004*^a^*** | **0.81**  **(0.67–0.96);**  **0.019*^a^*** | **0.81**  **(0.68–0.97);**  **0.024^a^** |
| **Duration of bright light (≥ 7000 lux)** | 678/79 526 |  |  |  |  |
| Below 0.45 hours | 357/39 935 | 1.00 (reference) | 1.00 (reference) | 1.00 (reference) | 1.00 (reference) |
| Above 0.45 hours | 321/39 591 | **0.86**  **(0.74–1.00);**  **0.047*^a^*** | **0.76**  **(0.63–0.92);**  **0.004*^a^*** | **0.81**  **(0.67–0.97);**  **0.023*^a^*** | **0.82**  **(0.68–0.98);**  **0.030^a^** |

**^a^** All *P* values remained significant after multiple testing with the FDR method. Cox proportional hazard regression was used to examine the associations. **Model 1** was adjusted for age and sex. **Model 2** was adjusted as in model 1 and for ethnicity, Townsend deprivation index, recruitment centre, education level, the season of accelerometer wear, photoperiod, and PM2.5 absorbance. **Model 3** was adjusted as in model 2 and for healthy diet score, vitamin D supplement use, smoking status, alcohol intake, MVPA, chronotype, and social isolation index. **Model 4** was adjusted as in model 3 and for obesity, history of diabetes, hypertension, traumatic brain injury, and hearing loss. **HR**: hazard ratio; **MVPA**: moderate to vigorous physical activity.

# **Supplemental table 10. Sensitivity analysis on the associations between daytime light exposure and dementia risk by using competing risk regression (Fine and Gray)**

| **Daytime light exposure** | **Events/n** | **Model 1**  HR (95% CI); *P* | **Model 2**  HR (95% CI); *P* | **Model 3**  HR (95% CI); *P* | **Model 4**  HR (95% CI); *P* |
| --- | --- | --- | --- | --- | --- |
| **Average daytime light** | 741/87 577 |  |  |  |  |
| Below 1000 lux | 315/40 553 | 1.00 (reference) | 1.00 (reference) | 1.00 (reference) | 1.00 (reference) |
| Above 1000 lux | 315/40 553 | 0.88  (0.76–1.01);  0.077 | **0.80**  **(0.68–0.95);**  **0.010** | **0.84**  **(0.71–0.99);**  **0.040*^a^*** | 0.85  (0.72–1.00);  0.051 |
| **Duration of bright light (≥ 3000 lux)** | 741/87 577 |  |  |  |  |
| Below 1.4 hours | 357/40 970 | 1.00 (reference) | 1.00 (reference) | 1.00 (reference) | 1.00 (reference) |
| Above 1.4 hours | 384/46 607 | **0.87**  **(0.75–0.99);**  **0.048** | **0.79**  **(0.67–0.93);**  **0.005** | **0.83**  **(0.70–0.98);**  **0.027** | **0.84**  **(0.71–0.99);**  **0.034** |
| **Duration of bright light (≥ 5000 lux)** | 741/87 577 |  |  |  |  |
| Below 0.7 hours | 324/37 088 | 1.00 (reference) | 1.00 (reference) | 1.00 (reference) | 1.00 (reference) |
| Above 0.7 hours | 417/50 489 | 0.88  (0.76–1.01);  0.071 | **0.79**  **(0.67–0.94);**  **0.007** | **0.84**  **(0.71–0.99);**  **0.038** | **0.84**  **(0.71–0.99);**  **0.047** |
| **Duration of bright light (≥ 7000 lux)** | 741/87 577 |  |  |  |  |
| Below 0.45 hours | 391/44 820 | 1.00 (reference) | 1.00 (reference) | 1.00 (reference) | 1.00 (reference) |
| Above 0.45 hours | 350/42 757 | 0.88  (0.77–1.02);  0.095 | **0.78**  **(0.66–0.93);**  **0.006** | **0.84**  **(0.71–0.99);**  **0.042** | **0.84**  **(0.70–0.99);**  **0.048** |

Competing risk regression was used to examine the associations. **Model 1** was adjusted for age and sex. **Model 2** was adjusted as in model 1 and for ethnicity, Townsend deprivation index, recruitment centre, education level, the season of accelerometer wear, photoperiod, and PM2.5 absorbance. **Model 3** was adjusted as in model 2 and for healthy diet score, vitamin D supplement use, smoking status, alcohol intake, MVPA, chronotype, and social isolation index. **Model 4** was adjusted as in model 3 and for obesity, history of diabetes, hypertension, traumatic brain injury, and hearing loss. **HR**: hazard ratio; **MVPA**: moderate to vigorous physical activity.

# **Supplemental table 11. Sensitivity analysis on the associations between daytime light exposure and dementia risk by using the dataset without imputation**

| **Daytime light exposure** | **Events/n** | **Model 1**  HR (95% CI); *P* | **Model 2**  HR (95% CI); *P* | **Model 3**  HR (95% CI); *P* | **Model 4**  HR (95% CI); *P****^a^*** |
| --- | --- | --- | --- | --- | --- |
| **Average daytime light** | 602/71 166 |  |  |  |  |
| Below 1000 lux | 287/32 674 | 1.00 (reference) | 1.00 (reference) | 1.00 (reference) | 1.00 (reference) |
| Above 1000 lux | 315/38 492 | 0.85  (0.73–1.00);  0.053 | **0.77 (0.64–0.92);**  **0.005** | **0.81**  **(0.68–0.97);**  **0.025** | **0.81**  **(0.68–0.98);**  **0.029*^a^*** |
| **Duration of bright light (≥ 3000 lux)** | 602/71 166 |  |  |  |  |
| Below 1.4 hours | 291/33 008 | 1.00 (reference) | 1.00 (reference) | 1.00 (reference) | 1.00 (reference) |
| Above 1.4 hours | 311/38 158 | **0.83**  **(0.71–0.98);**  **0.027** | **0.75**  **(0.62–0.900);**  **0.002** | **0.79**  **(0.66–0.95);**  **0.012** | **0.79**  **(0.66–0.96);**  **0.015*^a^*** |
| **Duration of bright light (≥ 5000 lux)** | 602/71 166 |  |  |  |  |
| Below 0.7 hours | 259/29 847 | 1.00 (reference) | 1.00 (reference) | 1.00 (reference) | 1.00 (reference) |
| Above 0.7 hours | 343/41 319 | 0.88  (0.75–1.03);  0.114 | **0.79**  **(0.65–0.96);**  **0.015** | 0.84  (0.69–1.01);  0.068 | 0.84  (0.70–1.02);  0.082 |
| **Duration of bright light (≥ 7000 lux)** | 602/71 166 |  |  |  |  |
| Below 0.45 hours | 317/36 073 | 1.00 (reference) | 1.00 (reference) | 1.00 (reference) | 1.00 (reference) |
| Above 0.45 hours | 285/35 093 | 0.87 (0.74–1.02);  0.076 | **0.75**  **(0.62–0.91);**  **0.004** | **0.80**  **(0.66–0.98);**  **0.028** | **0.81**  **(0.67–0.99);**  **0.035*^a^*** |

**^a^** All *P* values remained significant after multiple testing with the FDR method. Cox proportional hazard regression was used to examine the associations. **Model 1** was adjusted for age and sex. **Model 2** was adjusted as in model 1 and for ethnicity, Townsend deprivation index, recruitment centre, education level, the season of accelerometer wear, photoperiod, and PM2.5 absorbance. **Model 3** was adjusted as in model 2 and for healthy diet score, vitamin D supplement use, smoking status, alcohol intake, MVPA, chronotype, and social isolation index. **Model 4** was adjusted as in model 3 and for obesity, history of diabetes, hypertension, traumatic brain injury, and hearing loss. **HR**: hazard ratio; **MVPA**: moderate to vigorous physical activity.

# **Supplemental table 12. Sensitivity analysis on the associations between daytime light exposure and dementia risk by excluding participants with shift work history, visual disturbances or blindness**

| **Daytime light exposure** | **Events/n** | **Model 1**  HR (95% CI); *P* | **Model 2**  HR (95% CI); *P* | **Model 3**  HR (95% CI); *P* | **Model 4**  HR (95% CI); *P* |
| --- | --- | --- | --- | --- | --- |
| **Average daytime light** | 621/67 767 |  |  |  |  |
| Below 1000 lux | 297/31 198 | 1.00 (reference) | 1.00 (reference) | 1.00 (reference) | 1.00 (reference) |
| Above 1000 lux | 324/36 569 | 0.86  (0.74–1.01);  0.063 | **0.77**  **(0.65–0.93);**  **0.007** | **0.82**  **(0.69–0.99);**  **0.035** | **0.83**  **(0.69–0.99);**  **0.039** |
| **Duration of bright light (≥ 3000 lux)** | 621/67 767 |  |  |  |  |
| Below 1.4 hours | 301/31 531 | 1.00 (reference) | 1.00 (reference) | 1.00 (reference) | 1.00 (reference) |
| Above 1.4 hours | 320/36 236 | 0.84  (0.72–0.99);  0.035 | **0.76**  **(0.63–0.91);**  **0.003** | **0.80**  **(0.67–0.97);**  **0.019** | **0.81**  **(0.67–0.97);**  **0.021** |
| **Duration of bright light (≥ 5000 lux)** | 621/67 767 |  |  |  |  |
| Below 0.7 hours | 274/28 558 | 1.00 (reference) | 1.00 (reference) | 1.00 (reference) | 1.00 (reference) |
| Above 0.7 hours | 347/39 209 | 0.85  (0.72–0.99);  0.044 | **0.76**  **(0.63–0.91);**  **0.003** | **0.81**  **(0.67–0.97);**  **0.023** | **0.81**  **(0.67–0.98);**  **0.027** |
| **Duration of bright light (≥ 7000 lux)** | 621/67 767 |  |  |  |  |
| Below 0.45 hours | 329/34 667 | 1.00 (reference) | 1.00 (reference) | 1.00 (reference) | 1.00 (reference) |
| Above 0.45 hours | 292/33 100 | 0.88  (0.75–1.03);  0.099 | **0.76**  **(0.63–0.93);**  **0.006** | **0.82**  **(0.68–0.99);**  **0.044** | 0.83  (0.68–1.00);  0.053 |

Cox proportional hazard regression was used to examine the associations. **Model 1** was adjusted for age and sex. **Model 2** was adjusted as in model 1 and for ethnicity, Townsend deprivation index, recruitment centre, education level, season of accelerometer wear, photoperiod, and PM2.5 absorbance. **Model 3** was adjusted as in model 2 and for healthy diet score, vitamin D supplement use, smoking status, alcohol intake, MVPA, chronotype, and social isolation index. **Model 4** was adjusted as in model 3 and for obesity, history of diabetes, hypertension, traumatic brain injury, and hearing loss. **HR**: hazard ratio; **MVPA**: moderate to vigorous physical activity.

# **Supplemental table 13. Sensitivity analysis on the associations between daytime light exposure and dementia risk by additionally adjusting for *APOE* ε4 carrier status, depression and daily outdoor duration**

| **Daytime light exposure** | **Events/n** | **Model 5**  HR (95% CI); *P* |
| --- | --- | --- |
| **Average daytime light** | 741/87 577 |  |
| Below 1000 lux | 353/40 553 | 1.00 (reference) |
| Above 1000 lux | 388/47 024 | 0.85 (0.72–1.00); 0.051 |
| **Duration of bright light (≥ 3000 lux)** | 741/87 577 |  |
| Below 1.4 hours | 357/40 970 | 1.00 (reference) |
| Above 1.4 hours | 384/46 607 | **0.83 (0.70–0.98); 0.028** |
| **Duration of bright light (≥ 5000 lux)** | 741/87 577 |  |
| Below 0.7 hours | 324/37 088 | 1.00 (reference) |
| Above 0.7 hours | 417/50 489 | **0.84 (0.70–0.99); 0.043** |
| **Duration of bright light (≥ 7000 lux)** | 741/87 577 |  |
| Below 0.45 hours | 391/44 820 | 1.00 (reference) |
| Above 0.45 hours | 350/42 757 | **0.83 (0.70–0.99); 0.034** |

Cox proportional hazard regression was used to examine the associations. **Model 5** was adjusted as in model 4 and for *APOE* ε4 carrier status, depression and daily outdoor duration. **HR**: hazard ratio.

# **Supplemental table 14. Sensitivity analysis on the associations between photoperiod-defined daytime light exposure and dementia risk**

| **Daytime light exposure** | **Events/n** | **Model 1**  HR (95% CI); *P* | **Model 2**  HR (95% CI); *P* | **Model 3**  HR (95% CI); *P* | **Model 4**  HR (95% CI); *P* |
| --- | --- | --- | --- | --- | --- |
| **Average daytime light** | 741/87 577 |  |  |  |  |
| Below 1000 lux | 352/40 974 | 1.00 (reference) | 1.00 (reference) | 1.00 (reference) | 1.00 (reference) |
| Above 1000 lux | 389/46 603 | 0.88  (0.76–1.01);  0.076 | **0.83**  **(0.71–0.97);**  **0.021** | 0.87  (0.74–1.02);  0.09 | 0.88  (0.75–1.03);  0.112 |
| **Duration of bright light (≥ 3000 lux)** | 741/87 577 |  |  |  |  |
| Below 1.4 hours | 379/43 517 | 1.00 (reference) | 1.00 (reference) | 1.00 (reference) | 1.00 (reference) |
| Above 1.4 hours | 362/44 060 | **0.86**  **(0.74–0.99);**  **0.040** | **0.76**  **(0.64–0.90);**  **0.002** | **0.80**  **(0.67–0.95);**  **0.012** | **0.81**  **(0.68–0.96);**  **0.016** |
| **Duration of bright light (≥ 5000 lux)** | 741/87 577 |  |  |  |  |
| Below 0.7 hours | 335/38 641 | 1.00 (reference) | 1.00 (reference) | 1.00 (reference) | 1.00 (reference) |
| Above 0.7 hours | 406/48 936 | 0.89  (0.77–1.02);  0.103 | **0.79**  **(0.66–0.94);**  **0.009** | **0.84**  **(0.70–1.00);**  **0.048** | 0.85  (0.71–1.01);  0.061 |
| **Duration of bright light (≥ 7000 lux)** | 741/87 577 |  |  |  |  |
| Below 0.45 hours | 393/45 378 | 1.00 (reference) | 1.00 (reference) | 1.00 (reference) | 1.00 (reference) |
| Above 0.45 hours | 348/42 199 | 0.89  (0.77–1.03);  0.123 | **0.79**  **(0.66–0.94);**  **0.008** | 0.84  (0.70–1.00);  0.055 | 0.85  (0.71–1.01);  0.070 |

**^a^** All *P* values remained significant after multiple testing with the FDR method. Cox proportional hazard regression was used to examine the associations. **Model 1** was adjusted for age and sex. **Model 2** was adjusted as in model 1 and for ethnicity, Townsend deprivation index, recruitment centre, education level, the month of accelerometer wear, photoperiod, and PM2.5 absorbance. **Model 3** was adjusted as in model 2 and for healthy diet score, vitamin D supplement use, smoking status, alcohol intake, MVPA, chronotype, and social isolation index. **Model 4** was adjusted as in model 3 and for obesity, history of diabetes, hypertension, traumatic brain injury, and hearing loss. **HR**: hazard ratio; **MVPA**: moderate to vigorous physical activity.

# **Supplemental table 15. Interaction effects of daytime light exposure and average nighttime light on dementia risk**

| **Daytime light exposure** | **Multiplicative interaction**  **HR (95% CI); *P*** | **Additive interaction** | | |
| --- | --- | --- | --- | --- |
|  |  | **RERI (95% CI)** | **AP (95% CI)** | **S (95% CI)** |
| **Average daytime light level** |  |  |  |  |
| Below 1000 lux & Average nighttime light above 4.24 lux | 0.76  (0.57–1.02);  0.065 | −0.32  (−0.69–0.04) | −0.27  (−0.59–0.04) | 0.36  (0.12–1.09) |
| **Duration of bright light (≥ 3000 lux)** |  |  |  |  |
| Below 1.4 hours & Average nighttime light above 4.24 lux | **0.74**  **(0.55–0.99);**  **0.040** | −0.37  (−0.74–0.01) | −0.30  (−0.62–0.01) | **0.36**  **(0.13–0.99)** |
| **Duration of bright light (≥ 5000 lux)** |  |  |  |  |
| Below 0.7 hours & Average nighttime light above 4.24 lux | **0.73**  **(0.54–0.98);**  **0.034** | −0.38  (−0.76–0.001) | −0.32  (−0.65–0.007) | 0.33  (0.11–1.02) |
| **Duration of bright light (≥ 7000 lux)** |  |  |  |  |
| Below 0.45 hours & Average nighttime light above 4.24 lux | **0.68**  **(0.51–0.91);**  **0.009** | **−0.47**  **(−-0.86–-0.50)** | **−0.38**  **(−-0.71–-0.06)** | **0.32**  **(0.12–0.83)** |

Cox proportional hazard regression was used to examine the associations of daytime light exposure and average nighttime light with dementia risk, which were adjusted for age, sex, ethnicity, Townsend deprivation index, recruitment centre, education level, the season of accelerometer wear, photoperiod, PM2.5 absorbance, healthy diet score, vitamin D supplement use, smoking status, alcohol intake, MVPA, chronotype, social isolation index, obesity, history of diabetes, hypertension, traumatic brain injury and hearing loss. Multiplicative and additive interaction analyses were performed to examine the interaction effects. **AP:** attributable proportion due to interaction; **CI:** confidence interval; **HR**: hazard ratio; **MVPA**: moderate to vigorous physical activity; **RERI:** relative excess risk due to interaction; **S**: synergy index.

# **Supplemental table 16. Interaction effects of daytime light exposure and chronotype on dementia risk**

| **Daytime light exposure** | **Multiplicative interaction**  **HR (95% CI); *P*** | **Additive interaction** | | |
| --- | --- | --- | --- | --- |
|  |  | **RERI (95% CI)** | **AP (95% CI)** | **S (95% CI)** |
| **Average daytime light level** |  |  |  |  |
| Below 1000 lux & Evening | **0.70**  **(0.51–0.95);**  **0.024** | −0.51  (−0.15–0.12) | **−0.26**  **(−0.50-)-(−0.02)** | **0.65**  **(0.50–0.85)** |
| **Duration of bright light (≥ 3000 lux)** |  |  |  |  |
| Below 1.4 hours & Evening | 0.75  (0.55–1.03);  0.072 | −0.38  (−0.94–0.18) | −0.21  −0.44–0.03 | **0.69**  **(0.52–0.92)** |
| **Duration of bright light (≥ 5000 lux)** |  |  |  |  |
| Below 0.7 hours & Evening | 0.80  (0.58–1.09);  0.159 | −0.28  (−0.78–0.23) | −0.17  −0.42–0.08 | **0.70**  **(0.50–1.00)** |
| **Duration of bright light (≥ 7000 lux)** |  |  |  |  |
| Below 0.45 hours & Evening | **0.70**  **(0.51–0.96);**  **0.026** | −0.50  (−1.13–0.12) | **−0.25**  **(−0.47)-(−0.02)** | **0.67**  **(0.52–0.86)** |

Cox proportional hazard regression was used to examine the associations of daytime light exposure and chronotype with dementia risk, which were adjusted for age, sex, ethnicity, Townsend deprivation index, recruitment centre, education level, the season of accelerometer wear, photoperiod, PM2.5 absorbance, healthy diet score, vitamin D supplement use, smoking status, alcohol intake, MVPA, social isolation index, obesity, history of diabetes, hypertension, traumatic brain injury, and hearing loss. Multiplicative and additive interaction analyses were performed to examine the interaction effects. **AP:** attributable proportion due to interaction; **CI:** confidence interval; **HR**: hazard ratio; **MVPA**: moderate to vigorous physical activity; **RERI:** relative excess risk due to interaction; **S**: synergy index.

# **Supplemental table 17. Interaction effects of daytime light exposure and *APOE* ε4 status on dementia risk**

| **Daytime light exposure** | **Multiplicative interaction**  **HR (95% CI); *P*** | **Additive interaction** | | |
| --- | --- | --- | --- | --- |
|  |  | **RERI (95% CI)** | **AP (95% CI)** | **S (95% CI)** |
| **Average daytime light level** |  |  |  |  |
| Below 1000 lux & *APOE* ε4 carrier | 0.90  (0.66–1.23);  0.504 | 0.57  (−0.14–1.29) | **0.12**  **(0.01–0.23)** | **1.17**  **(1.01–1.37)** |
| **Duration of bright light (≥ 3000 lux)** |  |  |  |  |
| Below 1.4 hours & *APOE* ε4 carrier | 0.98  (0.72–1.35);  0.923 | 0.61  (−0.09–1.30) | **0.13**  **(0.02–0.24)** | **1.20**  **(1.03–1.41)** |
| **Duration of bright light (≥ 5000 lux)** |  |  |  |  |
| Below 0.7 hours & *APOE* ε4 carrier | 0.84  (0.61–1.15);  0.269 | 0.81  (−0.01–1.64) | **0.15**  **(0.04–0.26)** | **1.22**  **(1.06–1.42)** |
| **Duration of bright light (≥ 7000 lux)** |  |  |  |  |
| Below 0.45 hours & *APOE* ε4 carrier | 0.90  (0.66–1.24);  0.531 | 0.66  (−0.12–1.45) | **0.13**  **(0.02–0.25)** | **1.20**  **(1.02–1.41)** |

Cox proportional hazard regression was used to examine the associations of daytime light exposure and *APOE* ε4 status with dementia risk, which were adjusted for age, sex, ethnicity, Townsend deprivation index, recruitment centre, education level, the season of accelerometer wear, photoperiod, PM2.5 absorbance, healthy diet score, vitamin D supplement use, smoking status, alcohol intake, MVPA, chronotype, social isolation index, obesity, history of diabetes, hypertension, traumatic brain injury and hearing loss. Multiplicative and additive interaction analyses were performed to examine the interaction effects. **AP:** attributable proportion due to interaction; **CI:** confidence interval; **HR**: hazard ratio; **MVPA**: moderate to vigorous physical activity; **RERI:** relative excess risk due to interaction; **S**: synergy index.

# **Supplemental table 18. Interaction effects of daytime light exposure and age categories (< 65 and ≥ 65 years) on dementia risk**

| **Daytime light exposure** | **Multiplicative interaction**  **HR (95% CI); *P*** | **Additive interaction** | | |
| --- | --- | --- | --- | --- |
|  |  | **RERI (95% CI)** | **AP (95% CI)** | **S (95% CI)** |
| **Average daytime light** |  |  |  |  |
| Below 1000 lux & Older age | 0.95  (0.61–1.50);  0.840 | 1.48  (−0.20–3.17) | 0.15  (−0.01–0.30) | 1.19  (0.98–1.45) |
| **Bright daytime light above 3000 lux duration** |  |  |  |  |
| Below 1.4 hours & Older age | 1.00  (0.64–1.57);  0.995 | 1.66  (−0.02–3.34) | **0.16**  **(0.01–0.31)** | 1.22  (1.00–1.49) |
| **Bright daytime light above 5000 lux duration** |  |  |  |  |
| Below 0.7 hours & Older age | 0.89  (0.57–1.39);  0.600 | 1.38  (−0.41–3.16) | 0.13  (−0.03–0.29) | 1.17  (0.96–1.43) |
| **Bright daytime light above 7000 lux duration** |  |  |  |  |
| Below 0.45 hours & Older age | 0.90  (0.57–1.41);  0.636 | 1.40  (−0.40–3.21) | 0.13  (−0.02–0.29) | 1.17  (0.96–1.44) |

Cox proportional hazard regression was used to examine the associations of daytime light exposure and age with dementia risk, which were adjusted for sex, ethnicity, Townsend deprivation index, recruitment centre, education level, the season of accelerometer wear, photoperiod, PM2.5 absorbance, healthy diet score, vitamin D supplement use, smoking status, alcohol intake, MVPA, chronotype, social isolation index, obesity, history of diabetes, hypertension, traumatic brain injury, and hearing loss. Multiplicative and additive interaction analyses were performed to examine the interaction effects. **AP:** attributable proportion due to interaction; **CI:** confidence interval; **HR**: hazard ratio; **MVPA**: moderate to vigorous physical activity; **RERI:** relative excess risk due to interaction; **S**: synergy index.

# **Supplemental table 19. Interaction effects of daytime light exposure and sex on dementia risk**

| **Daytime light exposure** | **Multiplicative interaction**  **HR (95% CI); *P*** | **Additive interaction** | | |
| --- | --- | --- | --- | --- |
|  |  | **RERI (95% CI)** | **AP (95% CI)** | **S (95% CI)** |
| **Average daytime light level** |  |  |  |  |
| Below 1000 lux & Male | 0.88  (0.66–1.18);  0.389 | −0.06  (−0.38–0.26) | −0.03  (−0.21–0.14) | 0.93  (0.66–1.31) |
| **Duration of bright light (≥ 3000 lux)** |  |  |  |  |
| Below 1.4 hours & Male | 0.92  (0.69–1.23);  0.559 | −0.01  (−0.31–0.29) | −0.01  (−0.18–0.17) | 0.99  (0.67–1.45) |
| **Duration of bright light (≥ 5000 lux)** |  |  |  |  |
| Below 0.7 hours & Male | 0.96  (0.72–1.28);  0.784 | 0.03  (−0.26–0.31) | 0.02  (−0.16–0.19) | 1.05  (0.65–1.69) |
| **Duration of bright light (≥ 7000 lux)** |  |  |  |  |
| Below 0.45 hours & Male | 0.87  (0.65–1.17);  0.356 | −0.06  (−0.38–0.26) | −0.03  (−0.20–0.13) | 0.93  (0.68–1.28) |

Cox proportional hazard regression was used to examine the associations of daytime light exposure and sex with dementia risk, which were adjusted for age, ethnicity, Townsend deprivation index, recruitment centre, education level, the season of accelerometer wear, photoperiod, PM2.5 absorbance, healthy diet score, vitamin D supplement use, smoking status, alcohol intake, MVPA, chronotype, social isolation index, obesity, history of diabetes, hypertension, traumatic brain injury, and hearing loss. **AP:** attributable proportion due to interaction; **CI:** confidence interval; **HR**: hazard ratio; **MVPA**: moderate to vigorous physical activity; **RERI:** relative excess risk due to interaction; **S**: synergy index.

# **Supplemental table 20. Interaction effects of daytime light exposure and the timing of daytime light exposure on dementia risk**

| **Daytime light exposure** | **Multiplicative interaction**  **HR (95% CI); *P*** | **Additive interaction** | | |
| --- | --- | --- | --- | --- |
|  |  | **RERI (95% CI)** | **AP (95% CI)** | **S (95% CI)** |
| **Average daytime light level** |  |  |  |  |
| Below 1000 lux & Timing of light exposure | 0.81  (0.37–1.74);  0.584 | −0.25  (−1.40–0.90) | −0.24  (−1.14–0.67) | 0.13  (0.00–12327987.26) |
| **Duration of bright light (≥ 3000 lux)** |  |  |  |  |
| Below 1.4 hours & Timing of light exposure | 0.87  (0.40–1.88);  0.721 | −0.15  (−1.08–0.77) | −0.11  (−0.70–0.48) | 0.73  (0.24–2.19) |
| **Duration of bright light (≥ 5000 lux)** |  |  |  |  |
| Below 0.7 hours & Timing of light exposure | 0.87  (0.40–1.88);  0.721 | −0.15  (−1.08–0.77) | −0.11  (−0.70–0.48) | 0.73  (0.24–2.19) |
| **Duration of bright light (≥ 7000 lux)** |  |  |  |  |
| Below 0.45 hours & Timing of light exposure | 0.87  (0.40–1.88);  0.721 | −0.15  (−1.08–0.77) | −0.11  (−0.70–0.48) | 0.73  (0.24–2.19) |

Timing of daytime light exposure was defined as over 50% of total daytime light exposure occurring either before or after noon. Cox proportional hazard regression was used to examine the associations of daytime light exposure and the timing of daytime light exposure with dementia risk, which were adjusted for age, sex, ethnicity, Townsend deprivation index, recruitment centre, education level, the season of accelerometer wear, photoperiod, PM2.5 absorbance, healthy diet score, vitamin D supplement use, smoking status, alcohol intake, MVPA, chronotype, social isolation index, obesity, history of diabetes, hypertension, traumatic brain injury and hearing loss. Multiplicative and additive interaction analyses were performed to examine the interaction effects. **AP:** attributable proportion due to interaction; **CI:** confidence interval; **HR**: hazard ratio; **MVPA**: moderate to vigorous physical activity; **RERI:** relative excess risk due to interaction; **S**: synergy index.

# **Supplemental table 21. Interaction effects of daytime light exposure and depression on dementia risk**

| **Daytime light exposure** | **Multiplicative interaction**  **HR (95% CI); *P*** | **Additive interaction** | | |
| --- | --- | --- | --- | --- |
|  |  | **RERI (95% CI)** | **AP (95% CI)** | **S (95% CI)** |
| **Average daytime light level** |  |  |  |  |
| Below 1000 lux & Depression | 0.91  (0.54–1.54);  0.733 | −0.08  (−0.71–0.55) | −0.06  (−0.49–0.38) | 0.84  (0.26–2.66) |
| **Duration of bright light (≥ 3000 lux)** |  |  |  |  |
| Below 1.4 hours & Depression | 0.82  (0.48–1.39);  0.460 | −0.22  (−0.94–0.51) | −0.14  (−0.58–0.30) | 0.71  (0.31–1.60) |
| **Duration of bright light (≥ 5000 lux)** |  |  |  |  |
| Below 0.7 hours & Depression | 0.69  (0.41–1.19);  0.186 | −0.47  (−1.38–0.44) | −0.29  (−0.80–0.21) | 0.57  (0.28–1.13) |
| **Duration of bright light (≥ 7000 lux)** |  |  |  |  |
| Below 0.45 hours & Depression | 0.83  (0.49–1.40);  0.475 | −0.20  (−0.91–0.50) | −0.13  (−0.56–0.29) | 0.72  (0.32–1.59) |

Cox proportional hazard regression was used to examine the associations of daytime light exposure and depression with dementia risk, which were adjusted for age, sex, ethnicity, Townsend deprivation index, recruitment centre, education level, the season of accelerometer wear, photoperiod, PM2.5 absorbance, healthy diet score, vitamin D supplement use, smoking status, alcohol intake, MVPA, chronotype, social isolation index, obesity, history of diabetes, hypertension, traumatic brain injury and hearing loss. Multiplicative and additive interaction analyses were performed to examine the interaction effects. **AP:** attributable proportion due to interaction; **CI:** confidence interval; **HR**: hazard ratio; **MVPA**: moderate to vigorous physical activity; **RERI:** relative excess risk due to interaction; **S**: synergy index.

# **Supplemental table 22. Subgroup analysis on the associations between daytime light exposures and dementia risk stratified by average nighttime light level**

| **Daytime light exposure** | **Average nighttime light level categories** | **Events/n** | **Model 1**  HR (95% CI);  *P****^a^*** | **Model 2**  HR (95% CI);  *P****^a^*** | **Model 3**  HR (95% CI);  *P****^a^*** | **Model 4**  HR (95% CI);  *P****^a^*** |
| --- | --- | --- | --- | --- | --- | --- |
| **Average daytime light level** |  | 741/87 577 |  |  |  |  |
| Above 1000 lux  (vs. Below 1000 lux) | Lower | 369/43 789 | 1.00 (0.81–1.22); 0.969 | 0.94  (0.74–1.18); 0.598 | 0.98  (0.78–1.24); 0.863 | 0.99  (0.78–1.24); 0.910 |
|  | Higher | 372/43 788 | **0.75 (0.61–0.92); 0.006^a^** | **0.65**  **(0.51–0.83); 5.08e-4^a^** | **0.69**  **(0.54–0.88); 0.003^a^** | **0.70**  **(0.55–0.89); 0.004^a^** |
| **Duration of bright light (≥ 3000 lux)** |  | 741/87 577 |  |  |  |  |
| Above 1.4 hours  (vs. Below 1.4  hours) | Lower | 369/43 789 | 0.99 (0.81–1.22); 0.961 | 0.94  (0.74–1.18); 0.587 | 0.98  (0.78–1.24); 0.896 | 0.99  (0.78–1.25); 0.922 |
|  | Higher | 372/43 788 | **0.73 (0.59–0.89); 0.002^a^** | **0.62 (0.49–0.79); 1.26e-4^a^** | **0.66**  **(0.52–0.85); 9.63e-4^a^** | **0.67 (0.53–0.86); 0.002^a^** |
| **Duration of bright light (≥ 5000 lux)** |  | 741/87 577 |  |  |  |  |
| Above 0.7 hours  (vs. Below 0.7  hours) | Lower | 369/43 789 | 1.01 (0.82–1.23); 0.959 | 0.95  (0.75–1.20); 0.637 | 0.99  (0.79–1.26); 0.965 | 1.00  (0.79–1.27); 0.985 |
|  | Higher | 372/43 788 | **0.73 (0.59–0.90); 0.003^a^** | **0.62**  **(0.48–0.79); 1.66e-4^a^** | **0.66**  **(0.51–0.85); 0.001^a^** | **0.67**  **(0.52–0.86); 0.002^a^** |
| **Duration of bright light (≥ 7000 lux)** |  | 741/87 577 |  |  |  |  |
| Above 0.45 hours  (vs. Below 0.45  hours) | Lower | 369/43 789 | 1.07 (0.87–1.32); 0.521 | 1.02  (0.80–1.30); 0.873 | 1.08  (0.84–1.38); 0.541 | 1.09  (0.85–1.39); 0.499 |
|  | Higher | 372/43 788 | **0.72**  **(0.59–0.88); 0.002^a^** | **0.57**  **(0.44–0.73); 1.09e-05^a^** | **0.61**  **(0.47–0.79); 1.61e-4^a^** | **0.62**  **(0.48–0.80); 2.50e-4^a^** |

**^a^** All *P* values remained significant after multiple testing with the FDR method. Cox proportional hazard regression was used to examine the associations. **Model 1** was adjusted for age and sex. **Model 2** was adjusted as in model 1 and for ethnicity, Townsend deprivation index, recruitment centre, education level, season of accelerometer wear, photoperiod, and PM2.5 absorbance. **Model 3** was adjusted as in model 2 and for healthy diet score, vitamin D supplement use, smoking status, alcohol intake, MVPA, chronotype, and social isolation index. **Model 4** was adjusted as in model 3 and for obesity, history of diabetes, hypertension, traumatic brain injury, and hearing loss. **HR**: hazard ratio; **MVPA**: moderate to vigorous physical activity.

# **Supplemental table 23. Subgroup analysis on the associations between daytime light exposures and dementia risk stratified by chronotype**

| Daytime light exposure | Chronotype categories | Events/n | Model 1  HR (95% CI); *P****^a^*** | Model 2  HR (95% CI); *P****^a^*** | Model 3  HR (95% CI); *P****^a^*** | Model 4  HR (95% CI);*P****^a^*** |
| --- | --- | --- | --- | --- | --- | --- |
| **Average daytime light level** |  | 741/87 577 |  |  |  |  |
| Above 1000 lux  (vs. Below 1000 lux) | Morning | 511/57 477 | 0.98  (0.82–1.17); 0.844 | 0.92  (0.76–1.13); 0.443 | 0.96  (0.79–1.18); 0.698 | 0.97  (0.79–1.18); 0.369 |
|  | Evening | 230/30 100 | **0.67**  **(0.51–0.87); 0.002*^a^*** | **0.57**  **(0.42–0.77); 2.3e-4*^a^*** | **0.61**  **(0.45–0.83); 0.001*^a^*** | **0.62**  **(0.46–0.83); 0.002*^a^*** |
| **Duration of bright light (≥ 3000 lux)** |  | 741/87 577 |  |  |  |  |
| Above 1.4 hours  (vs. Below 1.4  hours) | Morning | 511/57 477 | 0.95  (0.80–1.13); 0.539 | 0.88  (0.72–1.07); 0.208 | 0.92  (0.75–1.12); 0.397 | 0.92  (0.75–1.13); 0.424 |
|  | Evening | 230/30 100 | **069**  **(0.53–0.89); 0.005*^a^*** | **0.59**  **(0.44–0.80);**  **6.1e-4*^a^*** | **0.64**  **(0.48–0.87); 0.004*^a^*** | **0.65**  **(0.48–0.87); 0.005*^a^*** |
| **Duration of bright light (≥ 5000 lux)** |  | 741/87 577 |  |  |  |  |
| Above 0.7 hours  (vs. Below 0.7  hours) | Morning | 511/57 477 | 0.94  (0.79–1.12); 0.482 | 0.86  (0.70–1.06); 0.164 | 0.90  (0.73–1.11); 0.340 | 0.91  (0.74–1.12); 0.369 |
|  | Evening | 230/30 100 | **0.73**  **(0.56–0.94); 0.019*^a^*** | **0.63**  **(0.46–0.85); 0.003*^a^*** | **0.68**  **(0.50–0.93); 0.014*^a^*** | **0.69**  **(0.51–0.93); 0.017*^a^*** |
| **Duration of bright light (≥ 7000 lux)** |  | 741/87 577 |  |  |  |  |
| Above 0.45 hours  (vs. Below 0.45  hours) | Morning | 511/57 477 | 0.99  (0.83–1.18); 0.936 | 0.92  (0.74–1.13); 0.426 | 0.97  (0.78–1.20); 0.764 | 0.97  (0.79–1.20); 0.805 |
|  | Evening | 230/30 100 | **0.66**  **(0.51–0.87); 0.003*^a^*** | **0.53**  **(0.38–0.72); 7.7e-5*^a^*** | **0.58**  **(0.42–0.80); 7.9e-4*^a^*** | **0.59**  **(0.43–0.81); 0.001*^a^*** |

**^a^** All *P* values remained significant after multiple testing with the FDR method. Cox proportional hazard regression was used to examine the associations. **Model 1** was adjusted for age and sex. **Model 2** was adjusted as in model 1 and for ethnicity, Townsend deprivation index, recruitment centre, education level, the season of accelerometer wear, photoperiod, and PM2.5 absorbance. **Model 3** was adjusted as in model 2 and for healthy diet score, vitamin D supplement use, smoking status, alcohol intake, MVPA, chronotype, and social isolation index. **Model 4** was adjusted as in model 3 and for obesity, history of diabetes, hypertension, traumatic brain injury, and hearing loss. **HR**: hazard ratio; **MVPA**: moderate to vigorous physical activity.

# **Supplemental table 24. Subgroup analysis on the associations between daytime light exposures and dementia risk stratified by *APOE* ε4 status**

| **Daytime light exposure** | ***APOE* ε4 status categories** | **Events/n** | **Model 1**  HR (95% CI); *P* | **Model 2**  HR (95% CI); *P* | **Model 3**  HR (95% CI); *P* | **Model 4**  HR (95% CI); *P* |
| --- | --- | --- | --- | --- | --- | --- |
| **Average daytime light level** |  | 741/87 577 |  |  |  |  |
| Above 1000 lux  (vs. Below 1000 lux) | Non-carrier | 335/66 801 | 0.85 (0.67–1.06); 0.152 | 0.84 (0.65–1.09); 0.197 | 0.89 (0.68–1.15); 0.369 | 0.89 (0.68–1.15); 0.369 |
|  | Carrier | 406/20 776 | 0.93 (0.75–1.15); 0.497 | 0.75  (0.59–0.97); 0.027 | 0.79  (0.62–1.02); 0.069 | 0.81 (0.63–1.03); 0.089 |
| **Duration of bright light (≥ 3000 lux)** |  | 741/87 577 |  |  |  |  |
| Above 1.4 hours  (vs. Below 1.4  hours) | Non-carrier | 335/66 801 | 0.86  (0.68–1.08); 0.190 | 0.86  (0.66–1.12); 0.258 | 0.91 (0.70–1.19); 0.494 | 0.91 (0.70–1.19); 0.497 |
|  | Carrier | 406/20 776 | 0.86 (0.69–1.07); 0.176 | **0.68 (0.53–0.88); 0.003** | 0.72 (0.56–0.92); 0.009 | **0.73 (0.57–0.94); 0.013** |
| **Duration of bright light (≥ 5000 lux)** |  | 741/87 577 |  |  |  |  |
| Above 0.7 hours  (vs. Below 0.7  hours) | Non-carrier | 335/66 801 | 0.78  (0.62–0.99); 0.038 | **0.76 (0.58–0.99); 0.041** | 0.80 (0.61–1.05); 0.114 | 0.80  (0.61–1.05); 0.115 |
|  | Carrier | 406/20 776 | 0.93 (0.75–1.16); 0.522 | **0.75**  **(0.58–0.97); 0.029** | 0.79 (0.61–1.02); 0.067 | 0.80 (0.62–1.03); 0.087 |
| **Duration of bright light (≥ 7000 lux)** |  | 741/87 577 |  |  |  |  |
| Above 0.45 hours  (vs. Below 0.45  hours) | Non-carrier | 335/66 801 | 0.84 (0.67–1.06); 0.138 | 0.82  (0.62–1.08); 0.164 | 0.88 (0.67–1.17); 0.388 | 0.88 (0.67–1.17); 0.392 |
|  | Carrier | 406/20 776 | 0.93 (0.75–1.16); 0.534 | **0.72**  **(0.56–0.93); 0.013** | **0.76 (0.59–0.99); 0.040** | 0.78 (0.60–1.01); 0.058 |

Cox proportional hazard regression was used to examine the associations. **Model 1** was adjusted for age and sex. **Model 2** was adjusted as in model 1 and for ethnicity, Townsend deprivation index, recruitment centre, education level,season of accelerometer wear, photoperiod, and PM2.5 absorbance. **Model 3** was adjusted as in model 2 and for healthy diet score, vitamin D supplement use, smoking status, alcohol intake, MVPA, chronotype, and social isolation index. **Model 4** was adjusted as in model 3 and for obesity, history of diabetes, hypertension, traumatic brain injury, and hearing loss. **HR**: hazard ratio; **MVPA**: moderate to vigorous physical activity.

# **Supplemental table 25. Associations between daytime light exposures (binary variables) and CRARs**

| **CRARs** | **Average daytime light** | | **Duration of bright light (≥ 3000 lux)** | | **Duration of bright light (≥ 5000 lux)** | | **Duration of bright light (≥ 7000 lux)** | |
| --- | --- | --- | --- | --- | --- | --- | --- | --- |
|  | **Beta (se)** | *P****^a^*** | **Beta (se)** | *P****^a^*** | **Beta (se)** | *P****^a^*** | **Beta (se)** | *P****^a^*** |
| L5 | −0.078 (0.008) | <0.001***^a^*** | −0.076 (0.008) | <0.001***^a^*** | −0.069 (0.008) | <0.001***^a^*** | −0.072 (0.008) | <0.001***^a^*** |
| M10 | 0.146 (0.005) | <0.001***^a^*** | 0.158 (0.005) | <0.001***^a^*** | 0.159 (0.005) | <0.001***^a^*** | 0.169 (0.005) | <0.001***^a^*** |
| RA | 0.186 (0.007) | <0.001***^a^*** | 0.196 (0.007) | <0.001***^a^*** | 0.198 (0.007) | <0.001***^a^*** | 0.208 (0.007) | <0.001***^a^*** |
| L5 onset | −0.036 (0.007) | <0.001***^a^*** | −0.031 (0.007) | <0.001***^a^*** | −0.034 (0.008) | <0.001***^a^*** | −0.032 (0.008) | <0.001***^a^*** |
| M10 onset | −0.057 (0.007) | <0.001***^a^*** | −0.053 (0.008) | <0.001***^a^*** | −0.053 (0.008) | <0.001***^a^*** | −0.049 (0.008) | <0.001***^a^*** |
| IS | 0.102 (0.007) | <0.001***^a^*** | 0.091 (0.007) | <0.001***^a^*** | 0.058 (0.007) | <0.001***^a^*** | 0.060 (0.008) | <0.001***^a^*** |
| IV | −0.179 (0.008) | <0.001***^a^*** | −0.199 (0.008) | <0.001***^a^*** | −0.206 (0.008) | <0.001***^a^*** | −0.238 (0.008) | <0.001***^a^*** |
| Amplitude | 0.023 (0.007) | <0.001***^a^*** | 0.028 (0.007) | <0.001***^a^*** | 0.035 (0.008) | <0.001***^a^*** | 0.036 (0.008) | <0.001***^a^*** |
| Acrophase | −0.048 (0.007) | <0.001***^a^*** | −0.050 (0.007) | <0.001***^a^*** | −0.055 (0.008) | <0.001***^a^*** | −0.070 (0.008) | <0.001***^a^*** |
| MESOR | 0.017 (0.007) | 0.002***^a^*** | 0.022 (0.007) | 0.002***^a^*** | 0.029 (0.007) | <0.001***^a^*** | 0.030 (0.008) | <0.001***^a^*** |

**^a^** All P values remained significant after multiple testing with the FDR method. The linear regression model was adjusted for age, sex, ethnicity, Townsend deprivation index, recruitment centre, education level, season of accelerometer wear, photoperiod, PM2.5 absorbance, healthy diet score, vitamin D supplement use, smoking status, alcohol intake, MVPA, chronotype, social isolation index, obesity, history of diabetes, hypertension, traumatic brain injury, and hearing loss. **CRARs,** circadian rest-activity rhythms; **L5**, the average activity during the least active 5-hour period; **M10**, the average activity during the most active 10-hour period; **RA**, relative amplitude; **L5 onset**, the onset time point of L5; **M10 onset**, the onset time point of M10; **IS**, interdaily stability; **IV**, intradaily variability; **Amplitude**, the amplitude of the rest-activity rhythm; **Acrophase**, the peak time of the rest-activity rhythm; **MESOR**, the midline estimating statistic of rhythm.

# **Supplemental table 26. Associations between daytime light exposures (binary variables) and brain structure**

| **Brain morphology** | **Average daytime light** | | **Duration of bright light (≥ 3000 lux)** | | **Duration of bright light (≥ 5000 lux)** | | **Duration of bright light (≥ 7000 lux)** | |
| --- | --- | --- | --- | --- | --- | --- | --- | --- |
|  | **Beta (se)** | *P* | **Beta (se)** | *P* | **Beta (se)** | *P* | **Beta (se)** | *P* |
| ***Volumes of cortical regions*** | | | | | | | | |
| bankssts_L | −0.007 (0.018) | 0.713 | 0.002 (0.018) | 0.928 | 0.012 (0.018) | 0.495 | 0.000 (0.019) | 0.994 |
| caudalanteriorcingulate_L | 0.033 (0.018) | 0.068 | 0.035 (0.018) | 0.050 | 0.041 (0.018) | 0.028 | **0.048 (0.019)** | **0.011** |
| caudalmiddlefrontal_L | −0.021 (0.018) | 0.240 | −0.015 (0.018) | 0.388 | −0.011 (0.018) | 0.552 | −0.031 (0.019) | 0.102 |
| cuneus_L | 0.008 (0.018) | 0.661 | 0.005 (0.018) | 0.799 | 0.002 (0.018) | 0.910 | −0.006 (0.019) | 0.753 |
| entorhinal_L | −0.005 (0.018) | 0.790 | 0.004 (0.018) | 0.812 | −0.008 (0.018) | 0.648 | −0.017 (0.019) | 0.366 |
| fusiform_L | 0.021 (0.018) | 0.240 | 0.025 (0.018) | 0.152 | 0.034 (0.018) | 0.059 | 0.019 (0.019) | 0.310 |
| inferiorparietal_L | −0.005 (0.018) | 0.791 | −0.006 (0.018) | 0.734 | −0.001 (0.018) | 0.947 | 0.001 (0.019) | 0.969 |
| inferiortemporal_L | 0.005 (0.018) | 0.794 | 0.004 (0.018) | 0.815 | 0.015 (0.018) | 0.416 | 0.028 (0.019) | 0.133 |
| isthmuscingulate_L | −0.007 (0.018) | 0.707 | −0.004 (0.018) | 0.804 | −0.010 (0.018) | 0.592 | −0.003 (0.019) | 0.875 |
| lateraloccipital_L | 0.017 (0.018) | 0.329 | 0.012 (0.018) | 0.497 | 0.009 (0.018) | 0.633 | 0.011 (0.019) | 0.544 |
| lateralorbitofrontal_L | −0.004 (0.018) | 0.839 | −0.006 (0.018) | 0.735 | −0.004 (0.018) | 0.833 | −0.009 (0.019) | 0.644 |
| lingual_L | 0.004 (0.018) | 0.820 | 0.003 (0.018) | 0.859 | −0.004 (0.018) | 0.807 | 0.014 (0.019) | 0.463 |
| medialorbitofrontal_L | −0.003 (0.018) | 0.877 | 0.004 (0.018) | 0.813 | 0.007 (0.018) | 0.702 | −0.006 (0.019) | 0.728 |
| middletemporal_L | −0.015 (0.017) | 0.394 | −0.022 (0.017) | 0.208 | −0.015 (0.018) | 0.396 | −0.007 (0.018) | 0.702 |
| parahippocampal_L | −0.003 (0.018) | 0.855 | 0.010 (0.018) | 0.559 | 0.001 (0.018) | 0.944 | −0.005 (0.019) | 0.771 |
| paracentral_L | −0.008 (0.018) | 0.663 | −0.007 (0.018) | 0.705 | 0.013 (0.018) | 0.486 | −0.019 (0.019) | 0.306 |
| parsopercularis_L | −0.018 (0.018) | 0.302 | −0.019 (0.018) | 0.289 | −0.008 (0.018) | 0.643 | −0.003 (0.019) | 0.853 |
| parsorbitalis_L | 0.004 (0.018) | 0.835 | 0.007 (0.018) | 0.676 | −0.007 (0.018) | 0.679 | −0.006 (0.019) | 0.737 |
| parstriangularis_L | −0.008 (0.018) | 0.644 | 0.004 (0.018) | 0.816 | −0.007 (0.018) | 0.681 | −0.016 (0.019) | 0.394 |
| pericalcarine_L | 0.013 (0.018) | 0.472 | 0.005 (0.018) | 0.802 | −0.005 (0.018) | 0.768 | −0.013 (0.019) | 0.488 |
| postcentral_L | −0.017 (0.018) | 0.350 | 0.001 (0.018) | 0.961 | 0.000 (0.018) | 0.992 | 0.007 (0.019) | 0.715 |
| posteriorcingulate_L | 0.018 (0.018) | 0.324 | 0.021 (0.018) | 0.252 | 0.044 (0.018) | 0.016 | 0.023 (0.019) | 0.229 |
| precentral_L | −0.006 (0.017) | 0.722 | −0.003 (0.018) | 0.854 | 0.004 (0.018) | 0.816 | −0.015 (0.018) | 0.403 |
| precuneus_L | −0.009 (0.017) | 0.605 | 0.007 (0.017) | 0.680 | 0.015 (0.018) | 0.396 | 0.002 (0.018) | 0.928 |
| rostralanteriorcingulate_L | −0.004 (0.018) | 0.805 | 0.004 (0.018) | 0.838 | 0.007 (0.018) | 0.723 | 0.010 (0.019) | 0.599 |
| rostralmiddlefrontal_L | 0.003 (0.017) | 0.858 | 0.008 (0.017) | 0.629 | −0.006 (0.018) | 0.743 | −0.008 (0.018) | 0.671 |
| superiorfrontal_L | −0.006 (0.017) | 0.721 | 0.000 (0.017) | 0.987 | 0.001 (0.018) | 0.932 | −0.021 (0.018) | 0.247 |
| superiorparietal_L | −0.008 (0.018) | 0.661 | −0.005 (0.018) | 0.765 | 0.001 (0.018) | 0.967 | −0.016 (0.019) | 0.392 |
| superiortemporal_L | −0.018 (0.018) | 0.296 | −0.006 (0.018) | 0.736 | 0.005 (0.018) | 0.774 | −0.019 (0.019) | 0.301 |
| supramarginal_L | −0.015 (0.018) | 0.387 | −0.004 (0.018) | 0.837 | 0.013 (0.018) | 0.473 | −0.003 (0.019) | 0.885 |
| frontalpole_L | 0.018 (0.018) | 0.315 | 0.025 (0.018) | 0.166 | 0.044 (0.018) | 0.016 | 0.013 (0.019) | 0.499 |
| transversetemporal_L | **−0.040 (0.018)** | **0.027** | **−0.041 (0.018)** | **0.022** | −0.035 (0.018) | 0.056 | **−0.056 (0.019)** | **0.003** |
| insula_L | −0.007 (0.018) | 0.680 | 0.002 (0.018) | 0.903 | 0.007 (0.018) | 0.723 | −0.016 (0.019) | 0.405 |
| bankssts_R | −0.012 (0.018) | 0.512 | −0.016 (0.018) | 0.384 | −0.016 (0.018) | 0.378 | −0.009 (0.019) | 0.616 |
| caudalanteriorcingulate_R | 0.006 (0.018) | 0.726 | 0.001 (0.018) | 0.936 | 0.010 (0.018) | 0.603 | 0.023 (0.019) | 0.231 |
| caudalmiddlefrontal_R | −0.028 (0.018) | 0.113 | −0.021 (0.018) | 0.239 | −0.018 (0.018) | 0.331 | −0.040 (0.019) | 0.032 |
| cuneus_R | 0.019 (0.018) | 0.297 | 0.010 (0.018) | 0.581 | 0.006 (0.018) | 0.744 | −0.002 (0.019) | 0.932 |
| entorhinal_R | 0.001 (0.018) | 0.964 | 0.006 (0.018) | 0.719 | −0.009 (0.018) | 0.645 | 0.005 (0.019) | 0.811 |
| fusiform_R | 0.027 (0.017) | 0.122 | 0.029 (0.018) | 0.095 | 0.032 (0.018) | 0.078 | 0.023 (0.019) | 0.205 |
| inferiorparietal_R | −0.017 (0.017) | 0.339 | −0.019 (0.017) | 0.285 | −0.010 (0.018) | 0.580 | −0.021 (0.018) | 0.257 |
| inferiortemporal_R | 0.003 (0.018) | 0.872 | 0.002 (0.018) | 0.923 | 0.016 (0.018) | 0.378 | 0.014 (0.019) | 0.461 |
| isthmuscingulate_R | −0.031 (0.018) | 0.085 | −0.027 (0.018) | 0.133 | −0.030 (0.018) | 0.104 | −0.027 (0.019) | 0.158 |
| lateraloccipital_R | 0.017 (0.018) | 0.343 | 0.020 (0.018) | 0.257 | 0.016 (0.018) | 0.380 | 0.021 (0.019) | 0.267 |
| lateralorbitofrontal_R | −0.008 (0.018) | 0.664 | −0.005 (0.018) | 0.764 | −0.011 (0.018) | 0.561 | 0.000 (0.019) | 1.000 |
| lingual_R | 0.007 (0.018) | 0.683 | −0.008 (0.018) | 0.663 | −0.011 (0.018) | 0.534 | 0.016 (0.019) | 0.388 |
| medialorbitofrontal_R | −0.010 (0.018) | 0.582 | −0.010 (0.018) | 0.553 | 0.002 (0.018) | 0.910 | 0.005 (0.019) | 0.786 |
| middletemporal_R | −0.030 (0.017) | 0.088 | −0.028 (0.017) | 0.109 | −0.024 (0.018) | 0.170 | −0.022 (0.018) | 0.230 |
| parahippocampal_R | −0.001 (0.018) | 0.941 | −0.001 (0.018) | 0.937 | −0.016 (0.018) | 0.371 | 0.010 (0.019) | 0.593 |
| paracentral_R | 0.020 (0.018) | 0.247 | 0.018 (0.018) | 0.299 | 0.021 (0.018) | 0.242 | 0.029 (0.019) | 0.116 |
| parsopercularis_R | −0.018 (0.018) | 0.308 | −0.026 (0.018) | 0.149 | −0.030 (0.018) | 0.100 | 0.001 (0.019) | 0.965 |
| parsorbitalis_R | −0.004 (0.017) | 0.806 | 0.002 (0.018) | 0.903 | −0.011 (0.018) | 0.532 | 0.009 (0.018) | 0.609 |
| parstriangularis_R | −0.025 (0.018) | 0.159 | −0.016 (0.018) | 0.372 | −0.011 (0.018) | 0.554 | −0.014 (0.019) | 0.449 |
| pericalcarine_R | 0.010 (0.018) | 0.582 | −0.008 (0.018) | 0.652 | −0.018 (0.018) | 0.338 | −0.002 (0.019) | 0.925 |
| postcentral_R | 0.009 (0.018) | 0.598 | 0.008 (0.018) | 0.654 | 0.004 (0.018) | 0.811 | 0.009 (0.019) | 0.625 |
| posteriorcingulate_R | 0.006 (0.018) | 0.722 | 0.014 (0.018) | 0.433 | 0.029 (0.018) | 0.117 | 0.023 (0.019) | 0.227 |
| precentral_R | −0.009 (0.017) | 0.592 | −0.004 (0.018) | 0.810 | 0.014 (0.018) | 0.439 | 0.000 (0.019) | 0.995 |
| precuneus_R | −0.026 (0.017) | 0.140 | −0.016 (0.018) | 0.357 | −0.002 (0.018) | 0.906 | −0.001 (0.019) | 0.952 |
| rostralanteriorcingulate_R | 0.003 (0.018) | 0.862 | 0.007 (0.018) | 0.710 | 0.012 (0.018) | 0.507 | −0.007 (0.019) | 0.697 |
| rostralmiddlefrontal_R | 0.004 (0.017) | 0.840 | 0.001 (0.017) | 0.937 | −0.009 (0.018) | 0.627 | −0.012 (0.018) | 0.516 |
| superiorfrontal_R | 0.016 (0.017) | 0.360 | 0.017 (0.017) | 0.326 | 0.024 (0.018) | 0.172 | 0.013 (0.018) | 0.474 |
| superiorparietal_R | −0.004 (0.017) | 0.835 | 0.000 (0.018) | 0.998 | 0.015 (0.018) | 0.414 | 0.008 (0.018) | 0.664 |
| superiortemporal_R | −0.003 (0.017) | 0.864 | −0.004 (0.017) | 0.837 | 0.007 (0.018) | 0.684 | −0.007 (0.018) | 0.706 |
| supramarginal_R | −0.021 (0.018) | 0.226 | −0.022 (0.018) | 0.216 | −0.010 (0.018) | 0.597 | −0.001 (0.019) | 0.939 |
| frontalpole_R | 0.004 (0.018) | 0.826 | 0.000 (0.018) | 0.982 | 0.011 (0.018) | 0.537 | 0.023 (0.019) | 0.232 |
| transversetemporal_R | −0.022 (0.018) | 0.220 | −0.019 (0.018) | 0.289 | −0.022 (0.018) | 0.238 | −0.031 (0.019) | 0.099 |
| insula_R | 0.001 (0.018) | 0.965 | 0.014 (0.018) | 0.441 | 0.012 (0.018) | 0.506 | −0.006 (0.019) | 0.755 |
| ***Areas of cortical regions*** | | | | | | | | |
| bankssts_L | −0.005 (0.018) | 0.773 | −0.003 (0.018) | 0.883 | 0.010 (0.018) | 0.575 | −0.004 (0.019) | 0.838 |
| caudalanteriorcingulate_L | **0.036 (0.018)** | **0.044** | 0.031 (0.018) | 0.085 | 0.036 (0.018) | 0.054 | **0.049 (0.019)** | **0.011** |
| caudalmiddlefrontal_L | −0.007 (0.018) | 0.691 | −0.002 (0.018) | 0.918 | 0.000 (0.018) | 0.999 | −0.007 (0.019) | 0.711 |
| cuneus_L | 0.027 (0.018) | 0.133 | 0.024 (0.018) | 0.184 | 0.014 (0.018) | 0.456 | 0.015 (0.019) | 0.422 |
| entorhinal_L | −0.008 (0.018) | 0.641 | −0.005 (0.018) | 0.771 | −0.012 (0.018) | 0.520 | −0.013 (0.019) | 0.504 |
| fusiform_L | **0.043 (0.018)** | **0.016** | **0.037 (0.018)** | **0.036** | **0.043 (0.018)** | **0.019** | **0.038 (0.019)** | **0.043** |
| inferiorparietal_L | 0.004 (0.018) | 0.818 | −0.001 (0.018) | 0.936 | 0.001 (0.018) | 0.939 | 0.010 (0.019) | 0.606 |
| inferiortemporal_L | 0.019 (0.018) | 0.296 | 0.011 (0.018) | 0.527 | 0.016 (0.018) | 0.385 | **0.045 (0.019)** | **0.016** |
| isthmuscingulate_L | −0.004 (0.018) | 0.814 | −0.005 (0.018) | 0.786 | −0.012 (0.018) | 0.506 | 0.000 (0.019) | 0.995 |
| lateraloccipital_L | **0.043 (0.018)** | **0.015** | 0.032 (0.018) | 0.075 | 0.028 (0.018) | 0.124 | **0.041 (0.019)** | **0.030** |
| lateralorbitofrontal_L | 0.003 (0.018) | 0.855 | −0.002 (0.018) | 0.914 | −0.008 (0.018) | 0.652 | −0.001 (0.019) | 0.947 |
| lingual_L | 0.033 (0.018) | 0.063 | 0.022 (0.018) | 0.224 | 0.006 (0.018) | 0.759 | 0.035 (0.019) | 0.066 |
| medialorbitofrontal_L | 0.008 (0.018) | 0.639 | 0.011 (0.018) | 0.553 | 0.003 (0.018) | 0.891 | 0.002 (0.019) | 0.896 |
| middletemporal_L | −0.004 (0.018) | 0.844 | −0.020 (0.018) | 0.261 | −0.011 (0.018) | 0.562 | 0.008 (0.019) | 0.676 |
| parahippocampal_L | −0.015 (0.018) | 0.395 | −0.014 (0.018) | 0.436 | −0.015 (0.018) | 0.401 | −0.018 (0.019) | 0.330 |
| paracentral_L | 0.011 (0.018) | 0.525 | 0.006 (0.018) | 0.722 | 0.016 (0.018) | 0.373 | −0.001 (0.019) | 0.953 |
| parsopercularis_L | −0.010 (0.018) | 0.574 | −0.013 (0.018) | 0.478 | −0.007 (0.018) | 0.716 | −0.001 (0.019) | 0.955 |
| parsorbitalis_L | 0.000 (0.018) | 0.980 | 0.000 (0.018) | 0.986 | −0.017 (0.018) | 0.358 | 0.001 (0.019) | 0.965 |
| parstriangularis_L | 0.003 (0.018) | 0.886 | 0.003 (0.018) | 0.873 | −0.013 (0.018) | 0.485 | −0.008 (0.019) | 0.663 |
| pericalcarine_L | **0.035 (0.018)** | **0.049** | 0.023 (0.018) | 0.210 | 0.002 (0.018) | 0.906 | 0.008 (0.019) | 0.681 |
| postcentral_L | −0.008 (0.018) | 0.637 | 0.000 (0.018) | 0.999 | −0.007 (0.018) | 0.704 | 0.013 (0.019) | 0.492 |
| posteriorcingulate_L | 0.013 (0.018) | 0.460 | 0.015 (0.018) | 0.417 | 0.023 (0.018) | 0.218 | 0.015 (0.019) | 0.429 |
| precentral_L | 0.009 (0.018) | 0.601 | 0.008 (0.018) | 0.659 | 0.017 (0.018) | 0.359 | 0.015 (0.019) | 0.439 |
| precuneus_L | 0.001 (0.018) | 0.963 | 0.014 (0.018) | 0.432 | 0.019 (0.018) | 0.305 | 0.015 (0.019) | 0.423 |
| rostralanteriorcingulate_L | 0.000 (0.018) | 0.986 | 0.002 (0.018) | 0.933 | 0.001 (0.018) | 0.953 | 0.002 (0.019) | 0.909 |
| rostralmiddlefrontal_L | 0.016 (0.018) | 0.368 | 0.014 (0.018) | 0.430 | −0.004 (0.018) | 0.823 | 0.008 (0.019) | 0.684 |
| superiorfrontal_L | 0.009 (0.018) | 0.606 | 0.012 (0.018) | 0.495 | 0.009 (0.018) | 0.640 | 0.003 (0.019) | 0.859 |
| superiorparietal_L | 0.000 (0.018) | 0.995 | −0.004 (0.018) | 0.832 | 0.001 (0.018) | 0.944 | −0.005 (0.019) | 0.800 |
| superiortemporal_L | −0.020 (0.018) | 0.274 | −0.016 (0.018) | 0.375 | −0.013 (0.018) | 0.477 | −0.029 (0.019) | 0.120 |
| supramarginal_L | −0.008 (0.018) | 0.636 | 0.002 (0.018) | 0.904 | 0.016 (0.018) | 0.374 | 0.005 (0.019) | 0.802 |
| frontalpole_L | 0.009 (0.018) | 0.628 | 0.010 (0.018) | 0.559 | 0.019 (0.018) | 0.288 | 0.011 (0.019) | 0.570 |
| transversetemporal_L | −0.022 (0.018) | 0.229 | −0.021 (0.018) | 0.235 | −0.021 (0.018) | 0.262 | −0.033 (0.019) | 0.081 |
| insula_L | −0.008 (0.018) | 0.648 | −0.006 (0.018) | 0.725 | 0.005 (0.018) | 0.795 | −0.011 (0.019) | 0.551 |
| bankssts_R | −0.011 (0.018) | 0.547 | −0.019 (0.018) | 0.292 | −0.019 (0.018) | 0.307 | −0.016 (0.019) | 0.406 |
| caudalanteriorcingulate_R | 0.002 (0.018) | 0.901 | −0.004 (0.018) | 0.833 | −0.001 (0.018) | 0.971 | 0.005 (0.019) | 0.792 |
| caudalmiddlefrontal_R | −0.013 (0.018) | 0.467 | −0.008 (0.018) | 0.670 | −0.007 (0.018) | 0.698 | −0.026 (0.019) | 0.165 |
| cuneus_R | 0.035 (0.018) | 0.052 | 0.030 (0.018) | 0.094 | 0.014 (0.018) | 0.433 | 0.016 (0.019) | 0.385 |
| entorhinal_R | −0.008 (0.018) | 0.646 | −0.003 (0.018) | 0.884 | −0.019 (0.018) | 0.294 | 0.003 (0.019) | 0.887 |
| fusiform_R | 0.029 (0.018) | 0.103 | 0.028 (0.018) | 0.110 | 0.033 (0.018) | 0.067 | 0.031 (0.019) | 0.098 |
| inferiorparietal_R | −0.011 (0.018) | 0.547 | −0.011 (0.018) | 0.523 | 0.000 (0.018) | 0.992 | −0.014 (0.019) | 0.470 |
| inferiortemporal_R | 0.009 (0.018) | 0.599 | 0.007 (0.018) | 0.679 | 0.025 (0.018) | 0.163 | 0.021 (0.019) | 0.268 |
| isthmuscingulate_R | −0.026 (0.018) | 0.148 | −0.023 (0.018) | 0.203 | −0.035 (0.018) | 0.056 | −0.020 (0.019) | 0.292 |
| lateraloccipital_R | **0.037 (0.018)** | **0.039** | **0.038 (0.018)** | **0.034** | 0.034 (0.018) | 0.063 | 0.032 (0.019) | 0.089 |
| lateralorbitofrontal_R | −0.007 (0.018) | 0.678 | −0.003 (0.018) | 0.850 | −0.011 (0.018) | 0.561 | 0.009 (0.019) | 0.630 |
| lingual_R | 0.017 (0.018) | 0.336 | 0.001 (0.018) | 0.937 | −0.010 (0.018) | 0.588 | 0.029 (0.019) | 0.133 |
| medialorbitofrontal_R | 0.004 (0.018) | 0.837 | −0.011 (0.018) | 0.536 | 0.003 (0.018) | 0.880 | 0.021 (0.019) | 0.274 |
| middletemporal_R | −0.026 (0.018) | 0.137 | −0.025 (0.018) | 0.155 | −0.023 (0.018) | 0.203 | −0.021 (0.019) | 0.270 |
| parahippocampal_R | 0.002 (0.018) | 0.916 | −0.007 (0.018) | 0.713 | −0.016 (0.018) | 0.382 | 0.000 (0.019) | 0.986 |
| paracentral_R | 0.038 (0.018) | 0.034 | 0.036 (0.018) | 0.049 | 0.043 (0.018) | 0.020 | 0.052 (0.019) | 0.006 |
| parsopercularis_R | −0.017 (0.018) | 0.349 | −0.024 (0.018) | 0.178 | −0.027 (0.018) | 0.150 | 0.001 (0.019) | 0.968 |
| parsorbitalis_R | 0.004 (0.018) | 0.834 | 0.015 (0.018) | 0.404 | −0.006 (0.018) | 0.759 | 0.019 (0.019) | 0.313 |
| parstriangularis_R | −0.019 (0.018) | 0.284 | −0.011 (0.018) | 0.536 | −0.017 (0.018) | 0.357 | −0.010 (0.019) | 0.599 |
| pericalcarine_R | 0.021 (0.018) | 0.249 | 0.010 (0.018) | 0.595 | −0.010 (0.018) | 0.598 | 0.003 (0.019) | 0.866 |
| postcentral_R | 0.002 (0.018) | 0.900 | 0.003 (0.018) | 0.850 | −0.007 (0.018) | 0.714 | −0.001 (0.019) | 0.977 |
| posteriorcingulate_R | −0.007 (0.018) | 0.679 | 0.000 (0.018) | 0.982 | 0.005 (0.018) | 0.773 | −0.001 (0.019) | 0.961 |
| precentral_R | 0.016 (0.018) | 0.387 | 0.019 (0.018) | 0.289 | 0.032 (0.018) | 0.085 | 0.027 (0.019) | 0.155 |
| precuneus_R | −0.011 (0.018) | 0.534 | −0.001 (0.018) | 0.974 | 0.011 (0.018) | 0.545 | 0.011 (0.019) | 0.551 |
| rostralanteriorcingulate_R | 0.006 (0.018) | 0.728 | 0.010 (0.018) | 0.571 | 0.012 (0.018) | 0.533 | −0.010 (0.019) | 0.609 |
| rostralmiddlefrontal_R | 0.021 (0.018) | 0.243 | 0.019 (0.018) | 0.299 | −0.001 (0.018) | 0.942 | 0.000 (0.019) | 0.992 |
| superiorfrontal_R | 0.033 (0.018) | 0.068 | 0.036 (0.018) | 0.043 | 0.030 (0.018) | 0.103 | 0.028 (0.019) | 0.147 |
| superiorparietal_R | 0.011 (0.018) | 0.526 | 0.016 (0.018) | 0.377 | 0.024 (0.018) | 0.191 | 0.019 (0.019) | 0.327 |
| superiortemporal_R | −0.006 (0.018) | 0.745 | −0.008 (0.018) | 0.656 | −0.004 (0.018) | 0.822 | −0.018 (0.019) | 0.354 |
| supramarginal_R | −0.010 (0.018) | 0.566 | −0.012 (0.018) | 0.493 | −0.004 (0.018) | 0.823 | 0.007 (0.019) | 0.696 |
| frontalpole_R | 0.014 (0.018) | 0.421 | 0.015 (0.018) | 0.399 | 0.022 (0.018) | 0.237 | 0.028 (0.019) | 0.142 |
| transversetemporal_R | 0.007 (0.018) | 0.691 | 0.010 (0.018) | 0.568 | −0.005 (0.018) | 0.776 | −0.022 (0.019) | 0.242 |
| insula_R | −0.011 (0.018) | 0.532 | −0.004 (0.018) | 0.829 | −0.011 (0.018) | 0.540 | −0.020 (0.019) | 0.295 |
| ***Mean thicknesses of cortical regions*** | | | | | | | | |
| bankssts_L | 0.002 (0.018) | 0.915 | 0.014 (0.018) | 0.435 | 0.016 (0.018) | 0.377 | 0.013 (0.019) | 0.485 |
| caudalanteriorcingulate_L | 0.000 (0.018) | 0.981 | 0.016 (0.018) | 0.381 | 0.014 (0.018) | 0.439 | 0.012 (0.019) | 0.531 |
| caudalmiddlefrontal_L | −0.021 (0.017) | 0.210 | −0.019 (0.017) | 0.261 | −0.020 (0.018) | 0.257 | −0.040 (0.018) | 0.027 |
| cuneus_L | −0.019 (0.018) | 0.294 | −0.019 (0.018) | 0.299 | −0.008 (0.018) | 0.679 | −0.022 (0.019) | 0.237 |
| entorhinal_L | 0.003 (0.018) | 0.879 | 0.014 (0.018) | 0.427 | −0.003 (0.018) | 0.887 | −0.016 (0.019) | 0.401 |
| fusiform_L | −0.028 (0.017) | 0.115 | −0.013 (0.018) | 0.451 | −0.008 (0.018) | 0.640 | −0.014 (0.019) | 0.435 |
| inferiorparietal_L | −0.011 (0.017) | 0.536 | −0.002 (0.017) | 0.907 | −0.004 (0.017) | 0.831 | −0.005 (0.018) | 0.774 |
| inferiortemporal_L | −0.024 (0.018) | 0.184 | −0.013 (0.018) | 0.475 | −0.001 (0.018) | 0.955 | −0.020 (0.019) | 0.279 |
| isthmuscingulate_L | 0.011 (0.018) | 0.518 | 0.013 (0.018) | 0.480 | 0.011 (0.018) | 0.565 | 0.014 (0.019) | 0.463 |
| lateraloccipital_L | −0.033 (0.018) | 0.060 | −0.027 (0.018) | 0.131 | −0.024 (0.018) | 0.190 | −0.034 (0.019) | 0.071 |
| lateralorbitofrontal_L | 0.006 (0.018) | 0.749 | 0.009 (0.018) | 0.630 | 0.016 (0.018) | 0.392 | −0.005 (0.019) | 0.773 |
| lingual_L | −0.028 (0.018) | 0.118 | −0.016 (0.018) | 0.376 | −0.009 (0.018) | 0.627 | −0.009 (0.019) | 0.641 |
| medialorbitofrontal_L | −0.002 (0.018) | 0.915 | 0.003 (0.018) | 0.878 | 0.009 (0.018) | 0.632 | −0.007 (0.019) | 0.708 |
| middletemporal_L | −0.009 (0.018) | 0.592 | 0.001 (0.018) | 0.971 | −0.002 (0.018) | 0.923 | −0.015 (0.019) | 0.412 |
| parahippocampal_L | 0.011 (0.018) | 0.545 | 0.027 (0.018) | 0.129 | 0.021 (0.018) | 0.256 | 0.014 (0.019) | 0.461 |
| paracentral_L | −0.025 (0.017) | 0.154 | −0.019 (0.017) | 0.272 | 0.000 (0.018) | 1.000 | −0.021 (0.018) | 0.252 |
| parsopercularis_L | −0.014 (0.017) | 0.409 | −0.011 (0.017) | 0.520 | −0.006 (0.018) | 0.730 | −0.006 (0.018) | 0.725 |
| parsorbitalis_L | 0.011 (0.018) | 0.522 | 0.016 (0.018) | 0.351 | 0.017 (0.018) | 0.358 | −0.002 (0.019) | 0.908 |
| parstriangularis_L | −0.023 (0.017) | 0.190 | −0.002 (0.017) | 0.896 | 0.005 (0.018) | 0.779 | −0.025 (0.018) | 0.172 |
| pericalcarine_L | **−0.037 (0.018)** | **0.037** | −0.030 (0.018) | 0.093 | −0.013 (0.018) | 0.484 | −0.039 (0.019) | 0.038 |
| postcentral_L | −0.015 (0.017) | 0.398 | 0.000 (0.017) | 0.981 | 0.005 (0.018) | 0.757 | −0.007 (0.018) | 0.717 |
| posteriorcingulate_L | 0.014 (0.018) | 0.419 | 0.023 (0.018) | 0.194 | 0.041 (0.018) | 0.024 | 0.016 (0.019) | 0.390 |
| precentral_L | −0.018 (0.017) | 0.287 | −0.011 (0.017) | 0.538 | −0.011 (0.017) | 0.523 | −0.031 (0.018) | 0.082 |
| precuneus_L | −0.011 (0.017) | 0.500 | −0.004 (0.017) | 0.796 | −0.001 (0.017) | 0.954 | −0.010 (0.018) | 0.568 |
| rostralanteriorcingulate_L | −0.008 (0.018) | 0.661 | 0.007 (0.018) | 0.691 | 0.016 (0.018) | 0.373 | 0.002 (0.019) | 0.927 |
| rostralmiddlefrontal_L | −0.018 (0.017) | 0.293 | −0.009 (0.017) | 0.603 | −0.005 (0.017) | 0.782 | −0.023 (0.018) | 0.201 |
| superiorfrontal_L | −0.027 (0.016) | 0.096 | −0.020 (0.016) | 0.218 | −0.018 (0.017) | 0.276 | −0.035 (0.017) | 0.042 |
| superiorparietal_L | −0.008 (0.017) | 0.657 | 0.000 (0.017) | 0.990 | 0.002 (0.018) | 0.894 | −0.013 (0.018) | 0.491 |
| superiortemporal_L | −0.002 (0.017) | 0.927 | 0.013 (0.017) | 0.449 | 0.024 (0.017) | 0.165 | 0.004 (0.018) | 0.829 |
| supramarginal_L | −0.007 (0.017) | 0.684 | −0.004 (0.017) | 0.795 | −0.007 (0.017) | 0.693 | −0.007 (0.018) | 0.699 |
| frontalpole_L | −0.001 (0.018) | 0.942 | 0.004 (0.018) | 0.805 | 0.012 (0.018) | 0.509 | −0.011 (0.019) | 0.550 |
| transversetemporal_L | −0.030 (0.018) | 0.092 | −0.029 (0.018) | 0.108 | −0.013 (0.018) | 0.478 | −0.030 (0.019) | 0.119 |
| insula_L | 0.008 (0.018) | 0.645 | 0.014 (0.018) | 0.431 | 0.009 (0.018) | 0.626 | −0.011 (0.019) | 0.550 |
| bankssts_R | 0.001 (0.018) | 0.951 | 0.004 (0.018) | 0.843 | −0.001 (0.018) | 0.977 | −0.003 (0.019) | 0.888 |
| caudalanteriorcingulate_R | 0.012 (0.018) | 0.519 | 0.008 (0.018) | 0.648 | 0.016 (0.018) | 0.395 | 0.038 (0.019) | 0.045 |
| caudalmiddlefrontal_R | −0.023 (0.017) | 0.171 | −0.020 (0.017) | 0.242 | −0.019 (0.018) | 0.275 | −0.027 (0.018) | 0.131 |
| cuneus_R | −0.004 (0.018) | 0.833 | −0.011 (0.018) | 0.529 | −0.001 (0.018) | 0.944 | −0.013 (0.019) | 0.489 |
| entorhinal_R | 0.012 (0.018) | 0.511 | 0.017 (0.018) | 0.334 | 0.020 (0.018) | 0.276 | 0.007 (0.019) | 0.719 |
| fusiform_R | 0.003 (0.017) | 0.846 | 0.007 (0.017) | 0.699 | 0.008 (0.018) | 0.639 | 0.005 (0.018) | 0.765 |
| inferiorparietal_R | −0.007 (0.017) | 0.690 | −0.007 (0.017) | 0.662 | −0.015 (0.017) | 0.396 | −0.007 (0.018) | 0.699 |
| inferiortemporal_R | −0.002 (0.018) | 0.896 | 0.001 (0.018) | 0.967 | −0.005 (0.018) | 0.765 | −0.004 (0.019) | 0.848 |
| isthmuscingulate_R | 0.013 (0.018) | 0.474 | 0.012 (0.018) | 0.505 | 0.022 (0.018) | 0.229 | 0.010 (0.019) | 0.611 |
| lateraloccipital_R | −0.018 (0.018) | 0.297 | −0.016 (0.018) | 0.366 | −0.015 (0.018) | 0.418 | −0.004 (0.019) | 0.834 |
| lateralorbitofrontal_R | 0.014 (0.018) | 0.430 | 0.013 (0.018) | 0.464 | 0.008 (0.018) | 0.676 | 0.001 (0.019) | 0.946 |
| lingual_R | −0.006 (0.018) | 0.721 | −0.012 (0.018) | 0.516 | −0.007 (0.018) | 0.686 | −0.004 (0.019) | 0.843 |
| medialorbitofrontal_R | −0.021 (0.018) | 0.246 | −0.004 (0.018) | 0.841 | −0.011 (0.018) | 0.550 | −0.023 (0.019) | 0.220 |
| middletemporal_R | −0.005 (0.017) | 0.775 | −0.002 (0.018) | 0.914 | −0.006 (0.018) | 0.749 | −0.003 (0.018) | 0.865 |
| parahippocampal_R | 0.000 (0.018) | 0.998 | 0.008 (0.018) | 0.634 | 0.000 (0.018) | 0.997 | 0.016 (0.019) | 0.397 |
| paracentral_R | −0.017 (0.017) | 0.333 | −0.013 (0.017) | 0.469 | −0.013 (0.018) | 0.457 | −0.018 (0.018) | 0.315 |
| parsopercularis_R | −0.004 (0.017) | 0.822 | −0.002 (0.017) | 0.894 | −0.002 (0.018) | 0.894 | 0.000 (0.018) | 0.991 |
| parsorbitalis_R | −0.012 (0.017) | 0.498 | −0.010 (0.017) | 0.555 | −0.012 (0.018) | 0.515 | −0.027 (0.018) | 0.143 |
| parstriangularis_R | −0.022 (0.017) | 0.206 | −0.016 (0.017) | 0.344 | −0.002 (0.018) | 0.906 | −0.013 (0.018) | 0.487 |
| pericalcarine_R | −0.015 (0.018) | 0.411 | −0.029 (0.018) | 0.111 | −0.017 (0.018) | 0.368 | −0.008 (0.019) | 0.675 |
| postcentral_R | 0.011 (0.017) | 0.525 | 0.009 (0.017) | 0.586 | 0.014 (0.018) | 0.422 | 0.018 (0.018) | 0.319 |
| posteriorcingulate_R | 0.023 (0.018) | 0.196 | 0.019 (0.018) | 0.292 | 0.032 (0.018) | 0.081 | 0.042 (0.019) | 0.026 |
| precentral_R | −0.022 (0.017) | 0.193 | −0.019 (0.017) | 0.273 | −0.016 (0.017) | 0.350 | −0.024 (0.018) | 0.190 |
| precuneus_R | −0.012 (0.017) | 0.483 | −0.011 (0.017) | 0.509 | −0.006 (0.018) | 0.721 | −0.008 (0.018) | 0.660 |
| rostralanteriorcingulate_R | −0.005 (0.018) | 0.770 | −0.007 (0.018) | 0.705 | 0.004 (0.018) | 0.812 | 0.012 (0.019) | 0.522 |
| rostralmiddlefrontal_R | −0.029 (0.017) | 0.094 | −0.025 (0.017) | 0.137 | −0.020 (0.017) | 0.265 | −0.020 (0.018) | 0.271 |
| superiorfrontal_R | −0.023 (0.016) | 0.157 | −0.026 (0.017) | 0.112 | −0.012 (0.017) | 0.486 | −0.020 (0.017) | 0.259 |
| superiorparietal_R | −0.013 (0.017) | 0.430 | −0.016 (0.017) | 0.363 | −0.010 (0.018) | 0.561 | −0.009 (0.018) | 0.610 |
| superiortemporal_R | 0.005 (0.017) | 0.751 | 0.005 (0.017) | 0.782 | 0.014 (0.017) | 0.408 | 0.009 (0.018) | 0.598 |
| supramarginal_R | −0.009 (0.017) | 0.578 | −0.007 (0.017) | 0.686 | −0.006 (0.017) | 0.727 | −0.006 (0.018) | 0.748 |
| frontalpole_R | −0.004 (0.018) | 0.826 | −0.007 (0.018) | 0.684 | −0.002 (0.018) | 0.929 | 0.000 (0.019) | 0.983 |
| transversetemporal_R | −0.035 (0.018) | 0.050 | −0.031 (0.018) | 0.084 | −0.024 (0.018) | 0.186 | −0.012 (0.019) | 0.523 |
| insula_R | 0.033 (0.018) | 0.067 | 0.034 (0.018) | 0.054 | 0.039 (0.018) | 0.032 | 0.030 (0.019) | 0.108 |
| ***Mean intensities of subcortical regions*** | | | | | | | | |
| 3rd-ventricle | 0.002 (0.015) | 0.913 | 0.001 (0.015) | 0.935 | −0.010 (0.016) | 0.525 | −0.007 (0.016) | 0.664 |
| 4th-ventricle | −0.018 (0.018) | 0.296 | −0.016 (0.018) | 0.383 | −0.023 (0.018) | 0.210 | −0.026 (0.019) | 0.167 |
| Brain-Stem | 0.006 (0.016) | 0.718 | 0.008 (0.016) | 0.618 | −0.008 (0.016) | 0.642 | 0.002 (0.017) | 0.912 |
| Optic-Chiasm | 0.038 (0.018) | 0.035 | 0.036 (0.018) | 0.046 | 0.029 (0.018) | 0.122 | 0.013 (0.019) | 0.492 |
| CC_Posterior | **0.040 (0.017)** | **0.021** | **0.038 (0.017)** | **0.028** | **0.035 (0.018)** | **0.048** | **0.042 (0.018)** | **0.021** |
| CC_Mid_Posterior | 0.004 (0.017) | 0.790 | 0.003 (0.017) | 0.879 | −0.016 (0.017) | 0.351 | −0.001 (0.018) | 0.968 |
| CC_Central | 0.010 (0.018) | 0.585 | 0.024 (0.018) | 0.167 | 0.008 (0.018) | 0.664 | 0.026 (0.019) | 0.159 |
| CC_Mid_Anterior | 0.011 (0.018) | 0.540 | 0.008 (0.018) | 0.658 | 0.011 (0.018) | 0.552 | 0.029 (0.019) | 0.122 |
| CC_Anterior | 0.033 (0.017) | 0.055 | 0.029 (0.017) | 0.095 | 0.017 (0.018) | 0.333 | 0.031 (0.018) | 0.093 |
| Lateral-Ventricle_L | 0.006 (0.017) | 0.721 | 0.008 (0.017) | 0.640 | −0.006 (0.017) | 0.712 | −0.012 (0.018) | 0.515 |
| Inf-Lat-Vent_L | 0.010 (0.017) | 0.544 | 0.011 (0.017) | 0.525 | 0.004 (0.017) | 0.835 | 0.012 (0.018) | 0.492 |
| Cerebellum-White-Matter_L | 0.002 (0.018) | 0.894 | 0.000 (0.018) | 0.991 | 0.004 (0.018) | 0.846 | −0.003 (0.019) | 0.878 |
| Cerebellum-Cortex_L | 0.015 (0.018) | 0.394 | 0.013 (0.018) | 0.479 | 0.013 (0.018) | 0.491 | 0.001 (0.019) | 0.937 |
| Thalamus-Proper_L | −0.005 (0.016) | 0.767 | 0.001 (0.017) | 0.930 | −0.019 (0.017) | 0.259 | −0.014 (0.017) | 0.431 |
| Caudate_L | 0.007 (0.018) | 0.697 | 0.008 (0.018) | 0.648 | −0.003 (0.018) | 0.857 | 0.002 (0.019) | 0.935 |
| Putamen_L | 0.003 (0.018) | 0.845 | 0.007 (0.018) | 0.684 | 0.003 (0.018) | 0.891 | 0.004 (0.019) | 0.848 |
| Pallidum_L | 0.021 (0.018) | 0.233 | 0.015 (0.018) | 0.388 | 0.000 (0.018) | 0.986 | 0.008 (0.019) | 0.656 |
| Hippocampus_L | 0.004 (0.018) | 0.829 | 0.007 (0.018) | 0.702 | −0.012 (0.018) | 0.494 | −0.002 (0.019) | 0.902 |
| Amygdala_L | −0.025 (0.018) | 0.167 | −0.019 (0.018) | 0.292 | −0.020 (0.018) | 0.264 | −0.014 (0.019) | 0.463 |
| Accumbens-area_L | −0.007 (0.017) | 0.674 | −0.011 (0.017) | 0.530 | −0.018 (0.017) | 0.289 | −0.007 (0.018) | 0.678 |
| VentralDC_L | 0.000 (0.017) | 0.984 | 0.000 (0.017) | 0.996 | −0.013 (0.017) | 0.465 | −0.004 (0.018) | 0.844 |
| Lateral-Ventricle_R | 0.006 (0.017) | 0.714 | 0.011 (0.017) | 0.532 | 0.004 (0.017) | 0.837 | −0.004 (0.018) | 0.826 |
| Inf-Lat-Vent_R | 0.000 (0.017) | 0.977 | −0.002 (0.017) | 0.896 | −0.002 (0.018) | 0.893 | 0.004 (0.018) | 0.834 |
| Cerebellum-White-Matter_R | −0.002 (0.018) | 0.925 | −0.006 (0.018) | 0.740 | 0.006 (0.018) | 0.734 | 0.009 (0.019) | 0.640 |
| Cerebellum-Cortex_R | 0.018 (0.018) | 0.297 | 0.012 (0.018) | 0.498 | 0.013 (0.018) | 0.476 | 0.004 (0.019) | 0.820 |
| Thalamus-Proper_R | 0.013 (0.016) | 0.429 | 0.019 (0.016) | 0.245 | 0.004 (0.017) | 0.831 | 0.002 (0.017) | 0.926 |
| Caudate_R | −0.010 (0.018) | 0.590 | −0.008 (0.018) | 0.642 | −0.012 (0.018) | 0.527 | −0.004 (0.019) | 0.849 |
| Putamen_R | −0.012 (0.018) | 0.509 | −0.012 (0.018) | 0.501 | −0.014 (0.018) | 0.435 | −0.008 (0.019) | 0.681 |
| Pallidum_R | −0.006 (0.018) | 0.753 | −0.013 (0.018) | 0.449 | −0.035 (0.018) | 0.052 | −0.013 (0.019) | 0.506 |
| Hippocampus_R | −0.010 (0.018) | 0.564 | −0.007 (0.018) | 0.699 | −0.023 (0.018) | 0.206 | −0.019 (0.019) | 0.315 |
| Amygdala_R | −0.008 (0.017) | 0.662 | −0.014 (0.018) | 0.424 | −0.017 (0.018) | 0.353 | −0.012 (0.019) | 0.501 |
| Accumbens-area_R | −0.003 (0.016) | 0.857 | −0.011 (0.016) | 0.501 | −0.014 (0.017) | 0.416 | −0.002 (0.017) | 0.925 |
| VentralDC_R | −0.013 (0.017) | 0.441 | −0.015 (0.017) | 0.396 | −0.026 (0.018) | 0.147 | −0.016 (0.018) | 0.369 |
| ***Volumes of subcortical regions*** | | | | | | | | |
| 3rd-ventricle | −0.016 (0.015) | 0.305 | −0.014 (0.015) | 0.373 | −0.015 (0.016) | 0.330 | −0.017 (0.016) | 0.290 |
| 4th-ventricle | 0.032 (0.018) | 0.073 | 0.033 (0.018) | 0.066 | 0.039 (0.018) | 0.029 | 0.024 (0.019) | 0.208 |
| Brain-Stem | 0.014 (0.018) | 0.415 | 0.025 (0.018) | 0.163 | 0.009 (0.018) | 0.602 | −0.005 (0.019) | 0.778 |
| Optic-Chiasm | 0.041 (0.017) | 0.017 | 0.030 (0.017) | 0.079 | 0.028 (0.018) | 0.107 | −0.003 (0.018) | 0.873 |
| CC_Posterior | 0.006 (0.018) | 0.724 | 0.006 (0.018) | 0.757 | 0.001 (0.018) | 0.970 | −0.008 (0.019) | 0.663 |
| CC_Mid_Posterior | 0.019 (0.017) | 0.286 | 0.024 (0.018) | 0.178 | −0.001 (0.018) | 0.967 | −0.006 (0.019) | 0.727 |
| CC_Central | 0.005 (0.017) | 0.777 | 0.010 (0.017) | 0.565 | −0.008 (0.018) | 0.652 | −0.010 (0.018) | 0.569 |
| CC_Mid_Anterior | 0.020 (0.017) | 0.237 | 0.033 (0.017) | 0.051 | 0.016 (0.017) | 0.348 | 0.008 (0.018) | 0.643 |
| CC_Anterior | **−0.037 (0.018)** | **0.035** | −0.023 (0.018) | 0.199 | −0.035 (0.018) | 0.054 | −0.030 (0.019) | 0.111 |
| Lateral-Ventricle_L | −0.008 (0.016) | 0.623 | −0.005 (0.016) | 0.761 | 0.006 (0.016) | 0.710 | −0.005 (0.017) | 0.763 |
| Inf-Lat-Vent_L | −0.017 (0.016) | 0.275 | −0.021 (0.016) | 0.187 | −0.011 (0.016) | 0.491 | −0.019 (0.017) | 0.265 |
| Cerebellum-White-Matter_L | 0.009 (0.017) | 0.593 | 0.020 (0.017) | 0.240 | 0.002 (0.018) | 0.924 | 0.010 (0.018) | 0.595 |
| Cerebellum-Cortex_L | −0.001 (0.017) | 0.946 | 0.016 (0.018) | 0.370 | 0.002 (0.018) | 0.892 | −0.005 (0.019) | 0.787 |
| Thalamus-Proper_L | 0.021 (0.016) | 0.198 | 0.023 (0.017) | 0.160 | 0.010 (0.017) | 0.566 | 0.001 (0.017) | 0.940 |
| Caudate_L | −0.010 (0.018) | 0.577 | −0.006 (0.018) | 0.746 | −0.017 (0.018) | 0.356 | −0.024 (0.019) | 0.205 |
| Putamen_L | 0.033 (0.017) | 0.055 | **0.041 (0.017)** | **0.019** | 0.031 (0.018) | 0.078 | 0.017 (0.018) | 0.368 |
| Pallidum_L | 0.009 (0.018) | 0.625 | 0.008 (0.018) | 0.662 | 0.018 (0.018) | 0.330 | −0.006 (0.019) | 0.741 |
| Hippocampus_L | 0.003 (0.017) | 0.874 | 0.009 (0.017) | 0.581 | −0.006 (0.017) | 0.721 | −0.017 (0.018) | 0.321 |
| Amygdala_L | 0.007 (0.016) | 0.657 | 0.018 (0.017) | 0.290 | 0.004 (0.017) | 0.798 | 0.008 (0.017) | 0.634 |
| Accumbens-area_L | 0.018 (0.016) | 0.262 | 0.025 (0.016) | 0.118 | 0.014 (0.016) | 0.389 | 0.009 (0.017) | 0.591 |
| VentralDC_L | 0.033 (0.017) | 0.048 | 0.037 (0.017) | 0.030 | 0.019 (0.017) | 0.277 | 0.002 (0.018) | 0.924 |
| Lateral-Ventricle_R | −0.017 (0.016) | 0.295 | −0.017 (0.016) | 0.285 | −0.013 (0.016) | 0.413 | −0.018 (0.017) | 0.280 |
| Inf-Lat-Vent_R | −0.017 (0.016) | 0.304 | −0.021 (0.016) | 0.204 | −0.010 (0.017) | 0.554 | −0.014 (0.017) | 0.427 |
| Cerebellum-White-Matter_R | 0.007 (0.017) | 0.675 | 0.012 (0.017) | 0.476 | −0.012 (0.018) | 0.490 | −0.005 (0.018) | 0.794 |
| Cerebellum-Cortex_R | −0.005 (0.017) | 0.783 | 0.013 (0.017) | 0.454 | 0.001 (0.018) | 0.947 | −0.010 (0.018) | 0.578 |
| Thalamus-Proper_R | **0.035 (0.017)** | **0.040** | **0.037 (0.017)** | **0.028** | 0.029 (0.017) | 0.090 | 0.012 (0.018) | 0.493 |
| Caudate_R | 0.000 (0.018) | 0.988 | 0.002 (0.018) | 0.891 | −0.010 (0.018) | 0.597 | −0.014 (0.019) | 0.467 |
| Putamen_R | 0.025 (0.017) | 0.144 | 0.031 (0.017) | 0.075 | 0.018 (0.018) | 0.319 | 0.012 (0.018) | 0.507 |
| Pallidum_R | 0.013 (0.018) | 0.481 | 0.013 (0.018) | 0.476 | 0.022 (0.018) | 0.239 | −0.003 (0.019) | 0.888 |
| Hippocampus_R | 0.005 (0.017) | 0.751 | 0.015 (0.017) | 0.355 | 0.004 (0.017) | 0.794 | −0.014 (0.018) | 0.421 |
| Amygdala_R | −0.013 (0.017) | 0.443 | −0.008 (0.017) | 0.631 | −0.007 (0.018) | 0.680 | −0.011 (0.018) | 0.557 |
| Accumbens-area_R | 0.017 (0.017) | 0.301 | 0.026 (0.017) | 0.123 | 0.015 (0.017) | 0.377 | 0.006 (0.018) | 0.730 |
| VentralDC_R | 0.018 (0.017) | 0.295 | 0.020 (0.017) | 0.228 | 0.008 (0.017) | 0.648 | −0.007 (0.018) | 0.706 |

The linear regression model was adjusted for age, sex, ethnicity, Townsend deprivation index, recruitment centre, education level, season of accelerometer wear, photoperiod, PM2.5 absorbance, healthy diet score, vitamin D supplement use, smoking status, alcohol intake, MVPA, chronotype, social isolation index, obesity, history of diabetes, hypertension, traumatic brain injury, and hearing loss. **CC**, corpus callosum; **_L**, left hemisphere; **_R**, right hemisphere. All FDR adjusted *P* values are not significant.

# **Supplemental table 27. Mediation analysis of associations between daytime light exposures (binary variables) and CRARs**

| **CRARs** | **ACME** | *P***_ACME** | | **ADE** | *P***_ADE** | | **Mediated** | | *P***_Mediated** |
| --- | --- | --- | --- | --- | --- | --- | --- | --- | --- |
| **Average daytime light level** | | | | | | | | | |
| **L5** | **358.060** | | **0.000** | **4838.444** | | **0.053** | | **0.067** | **0.036** |
| **M10** | **1575.997** | | **0.000** | **3518.321** | | **0.148** | | **0.302** | **0.032** |
| RA | 1362.355 | | 0.000 | 3114.412 | | 0.188 | | 0.294 | 0.062 |
| L5_onset | −145.340 | | 0.000 | 5174.509 | | 0.034 | | −0.028 | 0.039 |
| M10_onset | 14.149 | | 0.854 | 5053.021 | | 0.041 | | 0.003 | 0.855 |
| IS | −102.033 | | 0.447 | 5161.630 | | 0.035 | | −0.019 | 0.473 |
| IV | 421.381 | | 0.037 | 4583.370 | | 0.064 | | 0.081 | 0.079 |
| Amplitude | −13.997 | | 0.703 | 5097.220 | | 0.035 | | −0.002 | 0.719 |
| Acrophase | 55.158 | | 0.340 | 4997.600 | | 0.043 | | 0.010 | 0.369 |
| MESOR | −3.888 | | 0.893 | 5076.430 | | 0.035 | | 0.000 | 0.904 |
| **Duration of bright light (≥ 3000 lux)** | | | | | | | | | |
| **L5** | **344.003** | | **0.000** | **5207.951** | | **0.043** | | **0.060** | **0.029** |
| **M10** | **1690.095** | | **0.000** | **3852.987** | | **0.136** | | **0.299** | **0.024** |
| **RA** | **1420.192** | | **0.000** | **3467.468** | | **0.158** | | **0.282** | **0.042** |
| L5_onset | −124.119 | | 0.000 | 5654.669 | | 0.021 | | −0.022 | 0.024 |
| M10_onset | 14.217 | | 0.840 | 5483.330 | | 0.022 | | 0.003 | 0.842 |
| IS | −89.839 | | 0.440 | 5721.269 | | 0.017 | | −0.015 | 0.456 |
| IV | 448.726 | | 0.063 | 5058.440 | | 0.042 | | 0.080 | 0.086 |
| Amplitude | −18.071 | | 0.682 | 5524.774 | | 0.028 | | −0.003 | 0.693 |
| Acrophase | 59.373 | | 0.309 | 5470.522 | | 0.028 | | 0.010 | 0.328 |
| MESOR | −5.454 | | 0.880 | 5578.207 | | 0.022 | | −0.001 | 0.881 |
| **Duration of bright light (≥ 5000 lux)** | | | | | | | | | |
| **L5** | **315.704** | | **0.000** | **5059.886** | | **0.049** | | **0.057** | **0.037** |
| **M10** | **1693.704** | | **0.000** | **3530.260** | | **0.158** | | **0.316** | **0.036** |
| RA | 1445.407 | | 0.000 | 3270.597 | | 0.192 | | 0.296 | 0.056 |
| L5_onset | −138.215 | | 0.000 | 5424.936 | | 0.032 | | −0.025 | 0.039 |
| M10_onset | 13.444 | | 0.842 | 5240.290 | | 0.038 | | 0.002 | 0.848 |
| IS | −51.850 | | 0.495 | 5389.034 | | 0.034 | | −0.009 | 0.512 |
| IV | 472.161 | | 0.056 | 4701.690 | | 0.068 | | 0.088 | 0.098 |
| Amplitude | −22.377 | | 0.680 | 5339.418 | | 0.034 | | −0.004 | 0.694 |
| Acrophase | 63.116 | | 0.328 | 5229.960 | | 0.039 | | 0.011 | 0.349 |
| MESOR | −7.551 | | 0.884 | 5235.260 | | 0.036 | | −0.001 | 0.886 |
| **Duration of bright light (≥ 7000 lux)** | | | | | | | | | |
| **M10** | **1825.730** | | **0.000** | **3510.229** | | **0.185** | | **0.330** | **0.041** |
| RA | 1522.530 | | 0.000 | 3095.364 | | 0.232 | | 0.315 | 0.067 |
| L5_onset | −126.559 | | 0.000 | 5362.995 | | 0.038 | | −0.023 | 0.043 |
| M10_onset | 14.362 | | 0.837 | 5323.759 | | 0.042 | | 0.003 | 0.847 |
| IS | −55.255 | | 0.472 | 5420.910 | | 0.037 | | −0.010 | 0.494 |
| IV | 535.977 | | 0.064 | 4785.298 | | 0.073 | | 0.097 | 0.100 |
| Amplitude | −23.707 | | 0.685 | 5468.063 | | 0.036 | | −0.004 | 0.700 |
| Acrophase | 79.134 | | 0.351 | 5356.432 | | 0.044 | | 0.014 | 0.374 |
| MESOR | −8.444 | | 0.885 | 5420.007 | | 0.044 | | −0.001 | 0.883 |

The mediation analysis was adjusted for age, sex, ethnicity, Townsend deprivation index, recruitment centre, education level, season of accelerometer wear, photoperiod, PM2.5 absorbance, healthy diet score, vitamin D supplement use, smoking status, alcohol intake, MVPA, chronotype, social isolation index, obesity, history of diabetes, hypertension, traumatic brain injury, and hearing loss. **CRARs,** circadian rest-activity rhythms; **L5**, the average activity during the least active 5-hour period; **M10**, the average activity during the most active 10-hour period; **RA**, relative amplitude; **L5 onset**, the onset time point of L5; **M10 onset**, the onset time point of M10; **IS**, interdaily stability; **IV**, intradaily variability; **Amplitude**, the amplitude of the rest-activity rhythm; **Acrophase**, the peak time of the rest-activity rhythm; **MESOR**, the midline estimating statistic of rhythm. **ACME**, the average causal mediated effect; ***P*_ACME**, the *P* value of the significance of the average causal mediated effect; **ADE**, the average direct effect; ***P*_ADE**, the *P* value of the significance of the average direct effect; **Mediated**, the proportion of the mediation; ***P*_Mediated**, the *P* value of the significance of the mediation.

# **Supplemental table 28. Mediation analysis of associations between daytime light exposures (binary variables), brain structure and dementia risk**

| **Description** | **ACME** | *P***_ACME** | **ADE** | *P***_ADE** | **Mediated** | *P****^a^*_Mediated** |
| --- | --- | --- | --- | --- | --- | --- |
| **Average daytime light level** |  |  |  |  |  |  |
| Volume of transversetemporal (left hemisphere) | 135462298.400 | 0.023 | 77175397702.000 | <0.001 | 0.002 | **0.023*^a^*** |
| Area of caudalanteriorcingulate (left hemisphere) | 176.184 | 0.038 | 1906.866 | <0.001 | 0.084 | **0.038** |
| Area of fusiform (left hemisphere) | 191.426 | 0.018 | 1894.361 | <0.001 | 0.092 | **0.018*^a^*** |
| Area of pericalcarine (left hemisphere) | 174.928 | 0.051 | 1881.453 | <0.001 | 0.084 | 0.051 |
| Mean intensity of Optic-Chiasm (whole brain) | 50.113 | 0.040 | 2042.440 | <0.001 | 0.024 | **0.040** |
| Mean intensity of CC-Posterior (whole brain) | 256.949 | 0.029 | 1778.909 | <0.001 | 0.126 | **0.029** |
| Volume of VentralDC (left hemisphere) | −69.448 | 0.052 | 8922.518 | <0.001 | −0.008 | 0.052 |
| Volume of Thalamus-Proper (right hemisphere) | 18.086 | 0.043 | 2023.620 | <0.001 | 0.009 | **0.043** |
| **Duration of bright light (≥ 3000 lux)** |  |  |  |  |  |  |
| Area of lateraloccipital (right hemisphere) | 108.209 | 0.037 | 6869.401 | <0.001 | 0.015 | **0.037** |
| Area of paracentral (right hemisphere) | −164.582 | 0.054 | 7278.294 | <0.001 | −0.023 | 0.054 |
| Mean intensity of Optic-Chiasm (whole brain) | 48.090 | 0.051 | 7059.910 | <0.001 | 0.007 | 0.051 |
| Mean intensity of CC-Posterior (whole brain) | 239.974 | 0.035 | 6769.295 | <0.001 | 0.035 | **0.035*^a^*** |
| Volume of Putamen (left hemisphere) | 2.95e+22 | 0.0216 | 4.7e+24 | <0.001 | 0.006 | **0.022*^a^*** |
| Volume of VentralDC (left hemisphere) | −44.260 | 0.0308 | 7242.868 | <0.001 | −0.006 | **0.031*^a^*** |
| Volume of Thalamus-Proper (right hemisphere) | 16.853 | 0.0232 | 7039.323 | <0.001 | 0.002 | **0.023*^a^*** |
| **Duration of bright light (≥ 5000 lux)** |  |  |  |  |  |  |
| Volume of caudalanteriorcingulate (left hemisphere) | 215.938 | 0.024 | 8832.284 | <0.001 | 0.024 | **0.024*^a^*** |
| Volume of posteriorcingulate (left hemisphere) | 124.190 | 0.012 | 8899.788 | <0.001 | 0.014 | **0.012*^a^*** |
| Volume of frontalpole (left hemisphere) | 204.086 | 0.012 | 8901.493 | <0.001 | 0.022 | **0.012*^a^*** |
| Area of fusiform (left hemisphere) | 185.910 | 0.022 | 8870.955 | <0.001 | 0.020 | **0.022*^a^*** |
| Area of paracentral (right hemisphere) | −207.113 | 0.020 | 9295.255 | <0.001 | −0.023 | **0.020*^a^*** |
| Mean thickness of posteriorcingulate (left hemisphere) | 170.604 | 0.030 | 8880.212 | <0.001 | 0.019 | **0.030*^a^*** |
| Mean thickness of insula (right hemisphere) | 25.738 | 0.024 | 9089.618 | <0.001 | 0.003 | **0.024*^a^*** |
| Mean intensity of CC-Posterior (whole brain) | 217.780 | 0.059 | 8596.490 | <0.001 | 0.025 | 0.059 |
| Volume of 4th-Ventricle (whole brain) | −90.417 | 0.033 | 9134.707 | <0.001 | −0.010 | **0.033** |
| **Duration of bright light (≥ 7000 lux)** |  |  |  |  |  |  |
| Volume of transversetemporal (left hemisphere) | −188.266 | 0.004 | 6971.171 | <0.001 | −0.028 | **0.004*^a^*** |
| Volume of caudalmiddlefrontal (right hemisphere) | −313.120 | 0.030 | 7232.679 | <0.001 | −0.045 | **0.030*^a^*** |
| Area of caudalanteriorcingulate (left hemisphere) | 233.918 | 0.009 | 6641.372 | <0.001 | 0.034 | **0.009*^a^*** |
| Area of inferiortemporal (left hemisphere) | 32.318 | 0.020 | 6959.376 | <0.001 | 0.005 | **0.020*^a^*** |
| Area of lateraloccipital (left hemisphere) | 101.589 | 0.033 | 6775.049 | <0.001 | 0.015 | **0.033*^a^*** |
| Area of paracentral (right hemisphere) | −251.789 | 0.007 | 7159.654 | <0.001 | −0.037 | **0.007*^a^*** |
| Mean thickness of caudalmiddlefrontal (left hemisphere) | −232.920 | 0.024 | 7034.014 | <0.001 | −0.034 | **0.024*^a^*** |
| Mean thickness of pericalcarine (left hemisphere) | −199.672 | 0.030 | 6757.054 | <0.001 | −0.030 | **0.030*^a^*** |
| Mean thickness of posteriorcingulate (right hemisphere) | 194.981 | 0.024 | 6780.996 | <0.001 | 0.028 | **0.024*^a^*** |
| Mean intensity of CC-Posterior (whole brain) | 273.659 | 0.026 | 6670.035 | <0.001 | 0.039 | **0.026*^a^*** |

**^a^**All *P* values remained significant after multiple testing with the FDR method. The mediation analysis was adjusted for age, sex, ethnicity, Townsend deprivation index, recruitment centre, education level, the season of accelerometer wear, photoperiod, PM2.5 absorbance, healthy diet score, vitamin D supplement use, smoking status, alcohol intake, MVPA, chronotype, social isolation index, obesity, history of diabetes, hypertension, traumatic brain injury, and hearing loss. **ACME**, the average causal mediated effect; ***P*_ACME**, the *P* value of the significance of the average causal mediated effect; **ADE**, the average direct effect; ***P*_ADE**, the *P* value of the significance of the average direct effect; **Mediated**, the proportion of the mediation; ***P*_Mediated**, the *P* value of the significance of the mediation.

# **Supplemental table 29. Associations and mediation analysis of associations between daytime light exposures (binary variables) and serum vitamin D levels**

|  | **Average daytime light** | **Duration of bright light (≥ 3000 lux)** | **Duration of bright light (≥ 5000 lux)** | **Duration of bright light (≥ 7000 lux)** |
| --- | --- | --- | --- | --- |
| **Associations** |  |  |  |  |
| Beta (se) | 1.917 (0.163) | 2.070 (0.164) | 2.139 (0.169) | 2.502 (0.174) |
| *P* | <0.001 | <0.001 | <0.001 | <0.001 |
| **Mediation** |  |  |  |  |
| ACME | −112.139 | −127.445 | −126.081 | −154.127 |
| ***P*_ACME** | 0.334 | 0.302 | 0.314 | 0.306 |
| ADE | 4650.589 | 5646.309 | 4876.156 | 4705.310 |
| ***P*_ADE** | 0.095 | 0.040 | 0.084 | 0.104 |
| Mediated | −0.022 | −0.022 | −0.024 | −0.030 |
| ***P*_Mediated** | 0.401 | 0.328 | 0.375 | 0.392 |

The linear regression model and mediation analysis were both adjusted for age, sex, ethnicity, Townsend deprivation index, recruitment centre, education level, the season of accelerometer wear, photoperiod, PM2.5 absorbance, healthy diet score, vitamin D supplement use, smoking status, alcohol intake, MVPA, chronotype, social isolation index, obesity, history of diabetes, hypertension, traumatic brain injury, and hearing loss. **ACME**, the average causal mediated effect; ***P*_ACME**, the *P* value of the significance of the average causal mediated effect; **ADE**, the average direct effect; ***P*_ADE**, the *P* value of the significance of the average direct effect; **Mediated**, the proportion of the mediation; ***P*_Mediated**, the *P* value of the significance of the mediation.
